# Living off the land: Terrestrial-based diet and dairying in the farming communities of the Neolithic Balkans

**DOI:** 10.1371/journal.pone.0237608

**Published:** 2020-08-20

**Authors:** Darko Stojanovski, Ivana Živaljević, Vesna Dimitrijević, Julie Dunne, Richard P. Evershed, Marie Balasse, Adam Dowle, Jessica Hendy, Krista McGrath, Roman Fischer, Camilla Speller, Jelena Jovanović, Emmanuelle Casanova, Timothy Knowles, Lidija Balj, Goce Naumov, Anđelka Putica, Andrej Starović, Sofija Stefanović

**Affiliations:** 1 BioSense Institute, University of Novi Sad, Novi Sad, Serbia; 2 Laboratory for Bioarchaeology, Faculty of Philosophy, University of Belgrade, Beograd, Serbia; 3 Organic Geochemistry Unit, School of Chemistry, University of Bristol, Bristol, United Kingdom; 4 Archéozoologie, archéobotanique: Sociétés, Pratiques Environnements (AASPE), CNRS - Muséum national d’Histoire Naturelle, Paris, France; 5 Department of Biology, Bioscience Technology Facility, University of York, York, United Kingdom; 6 BioArch, Department of Archaeology, University of York, York, United Kingdom; 7 Target Discovery Institute, Nuffield Department of Medicine, University of Oxford, Oxford, United Kingdom; 8 Department of Anthropology, University of British Columbia, Vancouver, Canada; 9 BRAMS Facility, School of Chemistry, University of Bristol, Bristol, United Kingdom; 10 Museum of Vojvodina, Novi Sad, Serbia; 11 Museum of Macedonia, Skopje, Macedonia; 12 The Town Museum of Sombor, Sombor, Serbia; 13 National Museum in Belgrade, Belgrade, Serbia; University at Buffalo - The State University of New York, UNITED STATES

## Abstract

The application of biomolecular techniques to archaeological materials from the Balkans is providing valuable new information on the prehistory of the region. This is especially relevant for the study of the neolithisation process in SE Europe, which gradually affected the rest of the continent. Here, to answer questions regarding diet and subsistence practices in early farming societies in the central Balkans, we combine organic residue analyses of archaeological pottery, taxonomic and isotopic study of domestic animal remains and biomolecular analyses of human dental calculus. The results from the analyses of the lipid residues from pottery suggest that milk was processed in ceramic vessels. Dairy products were shown to be part of the subsistence strategies of the earliest Neolithic communities in the region but were of varying importance in different areas of the Balkan. Conversely, milk proteins were not detected within the dental calculus. The molecular and isotopic identification of meat, dairy, plants and beeswax in the pottery lipids also provided insights into the diversity of diet in these early Neolithic communities, mainly based on terrestrial resources. We also present the first compound-specific radiocarbon dates for the region, obtained directly from absorbed organic residues extracted from pottery, identified as dairy lipids.

## 1. Introduction

The earliest attempts in the domestication of wild plants such as barley, lentil, einkorn and emmer wheat, and animal species (cattle, sheep and goat), were identified in Southeast Anatolia and parts of the Levant, Syria, Iraq and Iran around 11 000 years ago [1–4]. This marked the beginnings of farming practices and the early stages of the Neolithic in the region. From here, this new socio-economic system spread westward over land and sea, reaching Cyprus and Central Anatolia, and by 6500 calBC, the coastal areas of Western Turkey, Thessaly and Macedonia. Between c. 6500 and 6000 calBC, it spread throughout the Balkan Peninsula and the southern parts of the Pannonian Plain [5–9]. Farming was introduced to the Balkans as a complete economic concept and the entire peninsula was occupied by farmers within five or six centuries, through a complex process of migration and interaction [10,11]. Genetic evidence, as well as the fact that the wild progenitors of most of the domesticates are not native to Europe, suggests that the early farmer-migrants from the East brought with them not only their agricultural knowledge, but also domesticated species of plants and animals [3,4,12–15].

The Balkan Peninsula, however, is a landmass with highly diverse topography, climate and environment. Here, for the first time, the early farmers would have met climatic challenges forcing them to adapt and modify their practices and the suite of domesticates they used [16,17]. This must have affected the subsistence patterns as agricultural products were the main source of food. The introduction of modern genetic, chemical and isotopic approaches in archaeology provided the means for gaining unprecedented insights into the human-environment relationship and, among other things, ancient human diet [10,18–22]. There is a growing body of evidence that the intensification of dairying in the Balkans followed a latitudinal direction [16–18,20] and the increased consumption of dairy products in sub-Continental and Continental environments of the north-central Balkans has been suggested as an attempt to supplement the losses suffered by adapting agricultural practices [16]. This specific part of the peninsula, however, remains underrepresented in bioarchaeological studies, in comparison to the Aegean coasts and Anatolia on one side, and the central and western Europe on the other.

**Fig 1:**
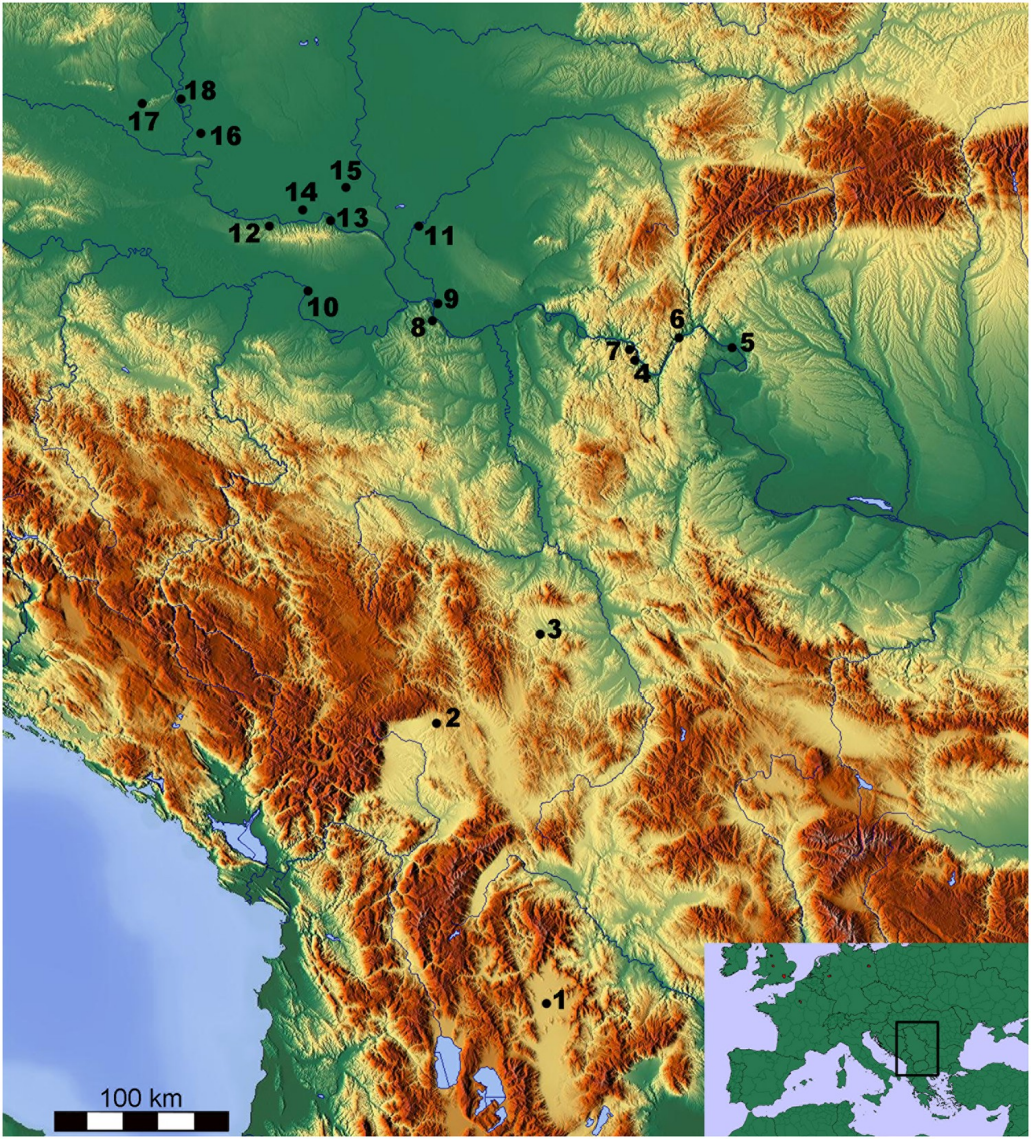
Partial map of the Balkan Peninsula. Sites mentioned in the text: 1-Vrbjanska Čuka, 2-Rudnik Kosovski, 3-Pločnik, 4-Lepenski Vir and Vlasac, 5-Ajmana, 6-Hajdučka Vodenica, 7-Padina, 8-Vinča, 9-Starčevo-Grad, 10-Gomolava, 11-Rutonjina Greda, 12-Golokut Vizić, 13-Sremski Karlovci, 14-Sajlovo, 15-Gospođinci, 16-Magareći Mlin, 17-Popova Zemlja, 18-Bački Monoštor (base map credit: © OpenStreetMap contributors, available under the Open Database Licence).

For this reason, we investigate the diet of the first farmers from a wider selection of sites from central and northern Balkans (Fig 1). More specifically, we consider the importance of milk and dairying practices and the diversity of the subsistence pattern. We combine data from molecular and stable carbon isotope analyses of organic residues from pottery, archaeozoological analyses and cattle mortality profiles, stable isotope analysis, i.e. nitrogen (Δ^15^N) and carbon (Δ^13^C), of cattle dentine collagen and metaproteomic analyses of human dental calculus. The δ^13^C isotopic analyses of lipids preserved in the pottery matrix provide direct insights into the cooking practices and the different foodstuffs processed and consumed by Neolithic people [23–26]. The relative importance of meat vs. dairy on one side, and ruminant vs. non-ruminant meat-bearing animals on the other, can thus be established in ancient diet. Taxonomic composition of studied faunal assemblages and mortality profiles of cattle, ubiquitous in the Central Balkans and probably the most important domestic animals due to their size and body mass [16,27], provide insights into animal husbandry strategies (i.e. meat exploitation and/or dairy production). Measuring cattle intra-tooth variation in nitrogen (δ^15^N) isotope ratios has proven to be a successful method to examine calf weaning patterns, with important implications for the better understanding of the availability of cattle milk for human consumption [28–30]. Biomolecular analyses of dental calculus are increasingly being applied to reconstruct the diet of past peoples. In particular, dental calculus represents a novel reservoir for detecting the consumption of whole milk or whey or products made thereof, through the detection of the milk protein ß-lactoglobulin (BLG) [31,32]. Furthermore, we applied a novel protocol for compound-specific radiocarbon analyses (CSRA) of fatty acids preserved in archaeological pottery vessels. This method, in addition to providing chronological information on both the typology of ceramic vessels, and thus associated sites/contexts, allows the direct dating of the occurrence of specific foodstuffs, such as carcass or dairy products [33–35]. In addition to the conventional radiocarbon dates obtained from some of the sites (shown S5.1 Fig in S5 File), here, we dated two potsherds containing dairy products, providing, for the first time, a direct date for milk use in the Balkans.

## 2. Results

### 2.1. Organic residue analyses of pottery

**Fig 2:**
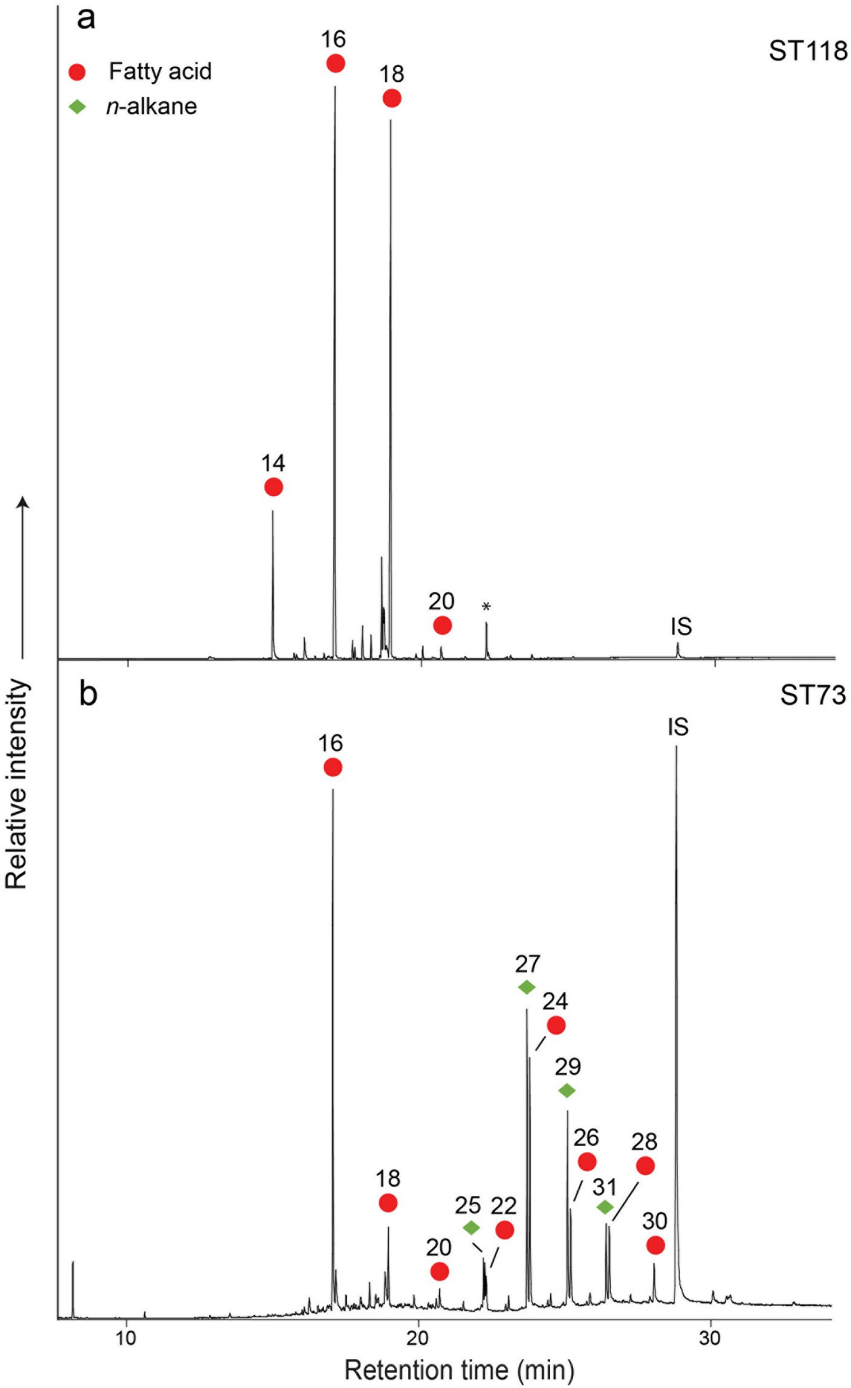
Partial gas chromatograms of trimethylsilylated Fatty Acid Methyl Esters (FAMEs) from Starčevo-Grad. a: potsherd ST118, showing a typical degraded animal fat profile [23,39], dominated by palmitic (C_16:0_) and stearic (C_18:0_) fatty acids (shown as red circles). b: potsherd ST73, displaying a series of long-chain fatty acids (red circles) and *n*-alkanes (green diamond), likely indicative of plant processing. IS indicates the internal standard.

Lipid analysis and interpretations were performed using established protocols described in detail in earlier publications [36,37] (see also S5 File). Interpretable lipids (in concentrations > 5 μg/g of sherd) were recovered from 55 (26%) of the 213 samples, with the recovery rate from each site being 32%, 21%, 26% and 18% from the sites of Starčevo-Grad, Magareći Mlin, Vrbjanska Čuka and Rutonjina Greda, respectively. This is comparable to recent studies in the region, for example, the overall recovery rate from sites in Neolithic Greece was 23% although rates varied between the Early, Middle and Late Neolithic [20]. Furthermore, the rate was 22% overall at five Early Neolithic sites in the Iron Gates region of the Lower Danube [22] and was 22% overall in 7 sites from the Hungarian plain and the Balkans area [16,20,22,25,38]. The majority of the lipid profiles (*n* = 47) comprise the free fatty acids, palmitic (C_16_) and stearic (C_18_), typical of a degraded animal fat, as the most abundant components (Fig 2a) [23,39]. Other lipid classes were detected, comprising aliphatic lipids including *n*-alkanes and *n*-alcohols, which will be discussed further below.

**Fig 3:**
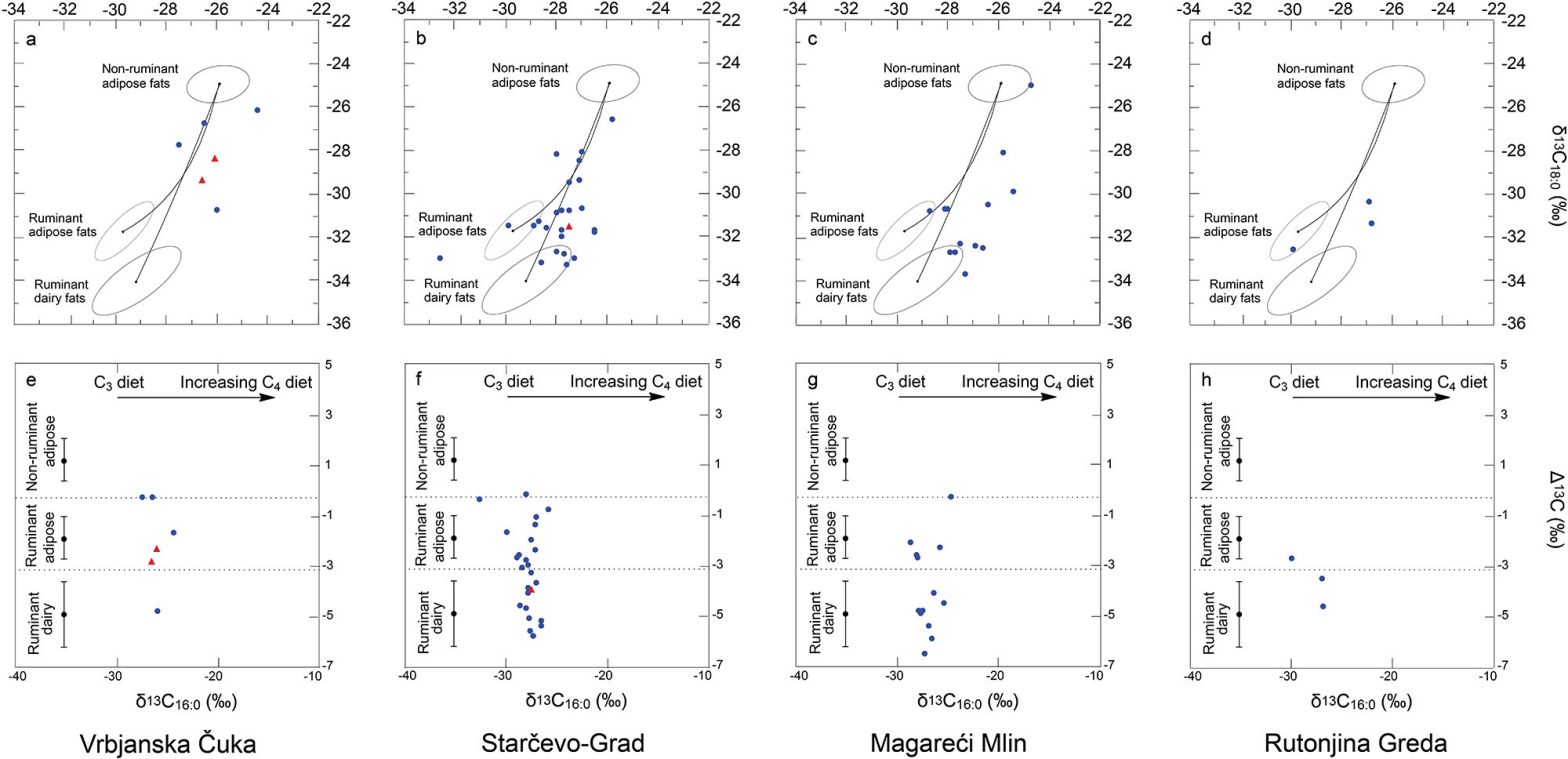
Graphs showing: **a, b, c, d**, δ^13^C values for the C_16:0_ and C_18:0_ fatty acids for archaeological fats extracted from Vrbjanska Čuka, Starčevo-Grad, Magareći Mlin and Rutonjina Greda vessels, respectively. The three fields correspond to the P = 0.684 confidence ellipses for animals raised on a strict C_3_ diet in Britain [41]. Each data point represents either: blue circle—terrestrial animal product; red triangle—plant/animal product mixture; Plots **e, f, g, h**, show the Δ^13^C (δ^13^C18:0 –δ^13^C_16:0_) values from the same potsherds. The ranges shown here represent the mean ± 1 s.d. of the Δ^13^C values for a global database comprising modern reference animal fats from Africa [42], UK (animals raised on a pure C_3_ diet) [36] Kazakhstan [43], Switzerland [44] and the Near East [45], published elsewhere.

GC-C-IRMS analyses were carried out on these 47 absorbed lipid residues to determine the δ^13^C values of the major fatty acids, C_16:0_ and C_18:0_, and ascertain the source of the lipids extracted [24,40]. The δ^13^C values obtained for modern reference animal fats, from animals raised on a pure C_3_ diet are grouped within confidence ellipses (±1s), onto which the values from the archaeological pottery have been plotted (Fig 3a–3d).

Ruminant dairy fats are differentiated from ruminant adipose fats when they display Δ^13^C values of less than -3.1 ‰, known as the universal proxy [42,46]. Evidence for the processing of secondary products, such as milk, butter and/or cheese, is found at all sites (total, *n* = 23), albeit in varying amounts, i.e. at Vrbjanska Čuka (*n* = 1, 17% of lipid bearing sherds at the site), Starčevo-Grad (*n* = 12, 48% of lipid bearing sherds at the site), Magareći Mlin (*n* = 8, 62% of lipid bearing sherds at the site) and Rutonjina Greda (*n* = 2, 67% of lipid bearing sherds at the site). A further 18 sherds plotted in the ruminant carcass region, Vrbjanska Čuka (*n* = 3, 50% of lipid bearing sherds at the site), Starčevo-Grad (*n* = 10, 40% of lipid bearing sherds at the site), Magareći Mlin (*n* = 4, 31% of lipid bearing sherds at the site) and Rutonjina Greda (*n* = 1, 33% of lipid bearing sherds at the site). No vessels plot solely within the non-ruminant (pig) region although a number of vessels from three sites, i.e. Vrbjanska Čuka (*n* = 2, 33% of lipid bearing sherds at the site), Starčevo-Grad (*n* = 3, 12% of lipid bearing sherds at the site) and Magareći Mlin (*n* = 1, 8% of lipid bearing sherds at the site), plot between the ruminant and non-ruminant region, suggesting some mixing of these products (Fig 3e–3h).

#### Aquatic/freshwater resource processing

All fatty acid methyl esters (FAMEs) were analysed by GC-MS in SIM mode to check for the presence of aquatic or freshwater biomarkers, namely ω-(*o*-alkyl phenyl) alkanoic acids (APAAs) and vicinal dihydroxy acid (DHYAs) which originate from the degradation of poly- and monounsaturated fatty acids found in marine or freshwater fats and oils. These are routinely used to detect marine/freshwater product processing [47–51]. Only one potsherd, Vessel ST35, contained the C_18_ and C_20_ APAAs, but DHYAs were not identified in any sherds. Hence, although freshwater aquatic products may have been mixed with terrestrial products in this vessel, albeit in low abundances, there is no evidence for sustained processing of freshwater resources at these sites.

#### Plant processing

Interestingly, a number of lipid profiles from Starčevo-Grad (*n* = 2, ST73, ST83) and Vrbjanska Čuka (n = 3, VC02, VC09, VC10) differ from those typical of animal fats, comprising sequences of even-numbered long-chain fatty acids (LCFAs), containing C_20_ to C_30_ carbon atoms, generally dominated by the C_24_ (Fig 2b). These LCFAs are strongly indicative either of an origin in leaf or stem epicuticular waxes [52–55] or, possibly, suberin [56–59], an aliphatic polyester found in all plants. Although primarily found on the surface of plant leaves, sheaths, stems and fruits, epicuticular waxes are also found associated with other plant organs, i.e. seed oils and coats, flowers, bark and husks [54]. However, these LCFAs are not diagnostic to families of plants and so cannot be used as anything other than a general indicator for plant processing.

Also present in the profiles is a series of odd-over-even long-chain *n*-alkanes, ranging from C_25_ to C_33_, generally dominated by the C_29_ n-alkanes (Fig 2b), albeit in low concentrations. Alkanes are also common components of waxes, usually occurring in low concentrations [60], although occasionally they are the dominant lipid, e.g. the leaf wax of *Cotyledon orbicularis* is almost entirely comprised of alkanes [61]. Long-chain *n*-alkane distributions occur in the range C_25_ to C_35_ [62], with an odd-over-even predominance [61]. The dominant chain lengths vary across plant taxonomic groups but the C_27_, C_29_, C_31_ and C_33_ homologues usually predominate [63]. Significantly, an analysis of leaf wax alkanes extracted from 93 species belonging to five subfamilies, *Bambusoideae*, *Pooideae*, *Arundinoideae*, *Chloridoideae* and *Panicoideae*, of the *Gramineae* (grass family), showed that the C_29_ and C_31_ *n*-alkanes dominated [64]. This combination of LCFAs and *n*-alkanes strongly suggests the processing of plant material, likely leafy plants and/or wild grasses, within these vessels. Interestingly, two sherds from the Vrbjanska Čuka pots (VC09, VC10) comprising plant lipid biomarkers, plot in the ruminant adipose region (Fig 3e, red triangles), suggesting mixing of leafy plants and carcass fats from cattle, sheep or goat, possibly in the form of a stew. In contrast, one vessel from Starčevo-Grad (ST83), which included LCFAs and *n*-alkanes, plotted within the dairy region (Fig 3f, red triangle).

#### Beeswax

**Fig 4:**
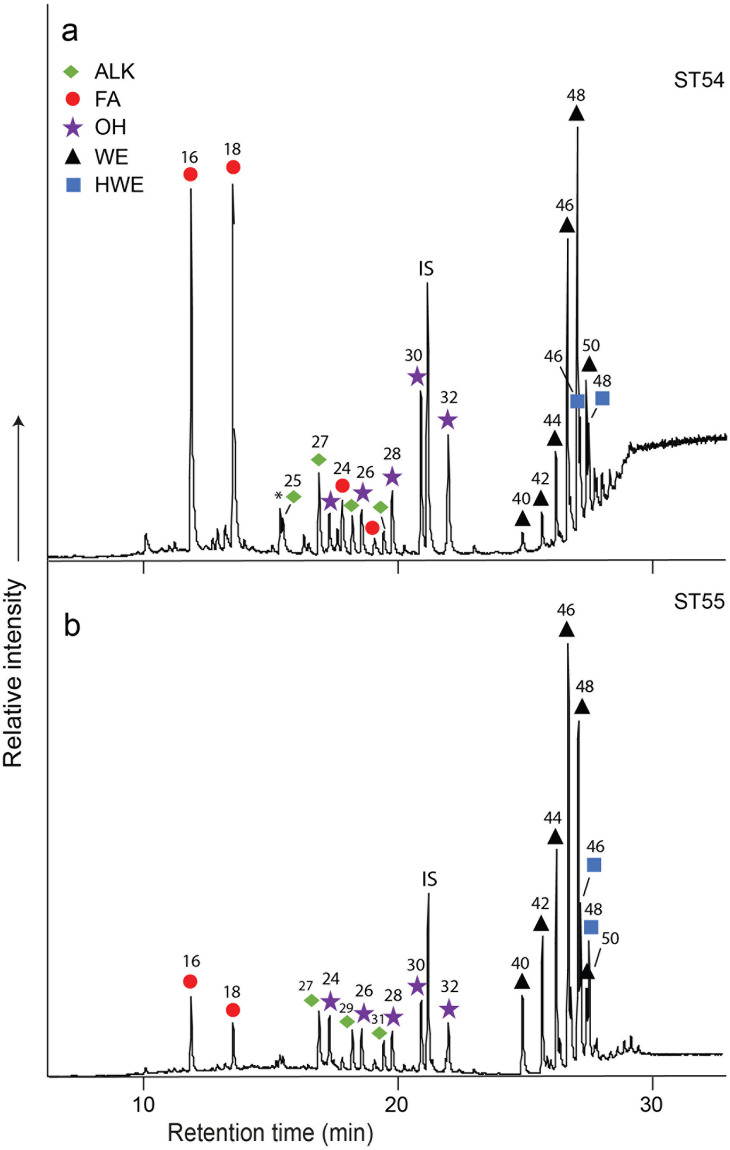
Partial high-temperature gas chromatograms of trimethylsilylated TLEs from the pottery extracts ST54 and ST55 containing beeswax. Red circles, *n*-alkanoic acids (fatty acids, FA); green rhombus, *n*-alkanes (ALK); purple star, *n*-alkanols (OH); black triangles, fatty acyl monoesters (WE); blue squares, hydroxyl fatty acyl monoesters (HWE) and IS, internal standard, C34 n-tetratriacontane. High abundances of C_16_ and C_18_ fatty acids, particularly potsherd ST54, are interpreted as originating from mammalian animal fats.

A number of lipid profiles (*n* = 7) comprised series of long-chain even-numbered *n*-alkanoic acids (C_20_ to C_26_), *n*-alkanols (C_24_ to C_32_), and *n*-alkanes (C_25_ to C_31_). These lipid profiles are generally indicative of the presence of beeswax and thus these vessels (four from Starčevo-Grad, ST54, ST55, ST82 and ST90 and three from Magareći Mlin, MM133, MM142 and MM149) were selected for further analysis by solvent extraction [36] to identify higher molecular weight compounds, wax esters, which would confirm the presence of beeswax in the sherds (see S5 File). Of these, the complex mixture of compounds seen across the profiles of ST54, ST55, ST82, ST90, MM142 and MM149 (Fig 4a and 4b) comprises the following homologous series, C_25_ to C_31_ carbon number *n*-alkanes displaying a unimodal distribution possessing a strong odd-over-even predominance and a series of C_24_–C_26_ long-chain alcohols in which *n*-tricontanol (C_30_) and *n*-dotricontanol (C_32_) were the major components. Eluting at longer retention times were a series of C_40_–C_54_ carbon number palmitic acid wax esters, confirming the presence of beeswax. A series of hydroxy palmitic acid wax esters, eluting at somewhat longer retention times than the wax esters, in which the C_46_ and C_48_ homologues were the most abundant components, are also present in samples ST54, ST55, ST82 and ST90. Sample MM133 did not contain any wax esters, simply a series of odd-numbered *n*-alkanes, and therefore cannot be unambiguously confirmed to contain beeswax. In summary, four potsherds were unambiguously confirmed to have contained beeswax, and two others highly probable.

Fresh beeswax comprises a complex mixture of aliphatic compounds consisting of series of homologues differing in chain-length by two methylene groups. Medium-chain *n*-alkanes range from C_23_ to C_31_ (with C_27_ dominating in *A*. *mellifera*), and *n*-alkanoic acids from C_20_ to C_36_ (usually dominated by lignoceric acid (C_24_)). Monoesters comprise predominantly alkyl palmitates (C_38_ to C_52_), with characteristic hydroxy monoesters comprising long-chain alcohols (C_24_ to C_38_) esterified mainly to hydroxypalmitic acid, ranging between C_40_ and C_54_ [65]. Although it is relatively resistant to degradation, the chromatographic profile of ancient beeswax often presents significant differences to that of contemporary beeswax [66,67]. For example, the free *n*-alkanols do not occur in fresh beeswax but are found in aged wax, due to hydrolysis of the wax esters. Furthermore, a preferential loss of lower carbon number *n*-alkanes may induce a modification of the *n*-alkane profile through time [68].

Finally, vessel ST54, displaying a typical beeswax profile, also contained high abundances of the major fatty acids, C_16:0_ and C_18:0_, typical of animal product processing (Fig 4a). This suggests that beeswax/honey may have been mixed with animal products in this vessel. These were also present in low amounts in vessels ST55 (Fig 4b) and ST82.

#### Direct dating of dairy residues

Two potsherds from the sites of Starčevo-Grad (ST118) and Vrbjanska Čuka (VC26) containing absorbed lipid residues in high concentrations were radiocarbon dated using a compound-specific approach (CSRA) [33,34]. The method employed is based on the extraction of C_16:0_ and C_18:0_ fatty acids residues from the clay matrix of potsherds and their isolation from exogenous contamination in the pots. In this case, the direct dating of dairy lipids from the potsherds, identified through their δ^13^C values, allows the date of use to be obtained.

The two radiocarbon dated vessels passed the quality assurance criteria on the identicality of the two FAs [33,34] which are combined to 6860 ± 46 BP (ST118, BRAMS-2837) and 6839 ± 47 BP (VC26, BRAMS-2838). These two radiocarbon ages are statistically identical supporting the contemporaneity of the two dated vessels. Vessel ST118 was in use in 5845–5650 calBC (95% probability), probably in 5793–5706 calBC (64% probability) or 5686–5676 calBC (4% probability). Vessel VC26 was in use between 5835–5825 calBC (2% probability) or 5813–5640 calBC (94% probability), probably in 5753–5666 calBC (68% probability). The calibrated ages plot firmly within the first half of the 6^th^ Millennium BC, confirming the antiquity of the dairy residues (see S5.1 Fig in S5 File). These dates agree with pre-existing chronologies obtained using conventional materials (an exhaustive list of ^14^C dates with references for SE Europe can be found in Reingruber and Thissen [69]).

The results provide the first CSRA on dairy residues from the Balkan area and suggests that dairy products were in use contemporaneously at Vrbjanska Čuka and Starčevo-Grad.

### 2.2. Taxonomic composition of faunal assemblages and cattle mortality profiles

In the three faunal assemblages studied, Magareći Mlin, Starčevo-Grad and Vrbjanska Čuka, the vast majority of remains originated from domestic ruminants—cattle, sheep, and goat (Table 1). Cattle remains were most numerous in the samples from Starčevo-Grad (44.0% of all mammal remains identified to the species/genus level) and Magareći Mlin (60.1% of all mammal remains identified to the species/genus level). This corresponds to the pattern observed on other Early Neolithic sites in the North-Central Balkans and southern fringes of the Pannonian Plain, indicative of a mixed herding strategy which involved ovicaprids and high proportions of cattle [16,27,70–73]. Considering the size and body mass of cattle, these animals most likely provided the bulk of protein and were the main suppliers of meat. On the other hand, analysed faunal assemblages from Macedonia [74–76 and references therein] indicate that sheep and goat husbandry was more prevalent in the Southern Balkans. Nevertheless, albeit small, the sample from Vrbjanska Čuka suggests that its inhabitants were herding both cattle (28.7% of all mammal remains identified to the species/genus level) and ovicaprids (50.5% of all mammal remains identified to the species/genus level).

**Table 1:** The taxonomic composition of faunal assemblages from the sites of Vrbjanska Čuka and Magareći Mlin (analysed within this study) and Starčevo-Grad (analysed by Clason 1980), expressed in NISP (number of identified specimens) frequencies.

TAXON		VRBJANSKA ČUKA	STARČEVO GRAD	MAGAREĆI MLIN
**Mammalia**	*Castor fiber*	**/**	**3** (0.2%)	**/**
	*Lepus europaeus*	**/**	**/**	**2** (0.3%)
	*Canis lupus*	**/**	**1** (0.1%)	**/**
	*Canis familiaris*	**2** (0.3%)	**7** (0.5%)	**/**
	*Vulpes vulpes*	**4** (0.6%)	**/**	**1** (0.2%)
	*Ursus arctos*	**/**	**1** (0.1%)	**/**
	*Meles meles*	**/**	**1** (0.1%)	**/**
	*Lutra lutra*	**/**	**2** (0.1%)	**/**
	*Felis silvestris*	**/**	**1** (0.1%)	**/**
	*Equus przewalskii*	**/**	**5** (0.3%)	**/**
	*Equus* sp.	**/**	**/**	**1** (0.2%)
	*Sus scrofa*	**/**	**125** (8.6%)	**3** (0.5%)
	*Sus domesticus*	**48** (7.4%)	**40** (2.8%)	**2** (0.3%)
	*Sus* sp.	**3** (0.5%)	**48** (3.3%)	**/**
	*Cervus elaphus*	**4** (0.6%)	**140** (9.7%)	**2** (0.3%)
	*Capreolus capreolus*	**1** (0.2%)	**10** (0.7%)	**7** (1.2%)
	*Bos primigenius*	**/**	**47** (3.2%)	**2** (0.3%)
	*Bos taurus*	**88** (13.6%)	**636** (43.9%)	**113** (18.7%)
	*Bos* sp.	**2** (0.3%)	**87** (6.0%)	**6** (1.0%)
	*Ovis aries*	**48** (7.4%)	**4** (0.3%)	**8** (1.3%)
	*Capra hircus*	**9** (1.4%)	**/**	**1** (0.2%)
	*Ovis/Capra*	**98** (15.1%)	**289** (19.9%)	**40** (6.6%)
	Ruminantia indet.	**4** (0.6%)	**3** (0.2%)	**22** (3.6%)
	Mammalia indet.	**338** (52.1%)	**/**	**393** (65.2%)
	**TOTAL**	**649**	**1450**	**603**
**Micromammalia**	*Talpa europaea*	**1**	**/**	**/**
	Insectivora indet.	**4**	**/**	**/**
	*Spalax leucodon*	**1**	**/**	**/**
	*Arvicola terrestris*	**1**	**/**	**/**
	Microtinae indet.	**12**	**/**	**/**
	*Mus musculus*	**3**	**/**	**/**
	Muridae indet.	**5**	**/**	**/**
	Rodentia indet.	**3**	**/**	**1**
**Aves**	*Anas* cf. *clypeata*	**/**	**1**	**/**
	*Anser anser*	**/**	**2**	**/**
	*Anser* cf. *anser fabalis*	**/**	**3**	**/**
	*Cygnus* cf. *olor*	**/**	**2**	**/**
	*Cygnus* cf. *cygnus*	**/**	**1**	**/**
	*Aquila* sp.	**/**	**1**	**/**
	*Circus* sp.	**/**	**1**	**/**
	*Grus grus*	**/**	**2**	**/**
	*Otis tarda*	**/**	**5**	**/**
	*Numenius arquata*	**/**	**1**	**/**
	Aves indet.	**20**	**/**	**/**
**Herpetofauna**	*Anura* sp.	**15**	**/**	**/**
	*Lacerta viridis*	**3**	**/**	**/**
	Testudines indet.	**/**	**/**	**4**
	Herpetofauna indet.	**77**	**/**	**/**
**Pisces**	*Cyprinus carpio*	**/**	**1**	**/**
	Cyprinidae indet.	**34**	**/**	**/**
	Salmonidae indet.	**9**	**/**	**/**
	*Silurus glanis*	**/**	**17**	**1**
	Pisces indet.	**12**	**/**	**/**
**Mollusca**	*Viviparus viviparus*	**/**	**1**	**/**
	*Viviparus* sp.	**/**	**/**	**36**
	*Succinea oblonga*	**/**	**/**	**2**
	*Unio crassus*	**177**	**3**	**3**
	*Unio pictorum*	**/**	**/**	**2**
	*Unio tumidus*	**/**	**/**	**1**
	*Unio* sp.	**23**	**/**	**9**
	*Anodonta* sp.	**3**	**/**	**/**

The contribution of domestic ruminants (cattle, sheep and goat) are shaded.

The paucity of the three studied assemblages hinders our understanding of cattle slaughter patterns. At Magareći Mlin and Starčevo, this could be related to the techniques used to recover animal bones (see S5 File). However, in case of Vrbjanska Čuka, both hand-collection and flotation were employed, so the low MNIs could be related to deposition practices, i.e. cleaning of building floors prior to abandonment [77]. Nevertheless, some trends may be discerned. Profiles are constructed for Starčevo-Grad (MNI = 14) and Magareći Mlin (MNI = 15), whereas the minimal number of individuals based on dental elements from Vrbjanska Čuka was insufficient for profile construction (MNI = 4). Nevertheless, given the evidence of milk exploitation and processing at the first two sites by means of lipid residue analysis, it is worth cross-referencing this data with cattle mortality profiles, and, ultimately, completing the assessment using the results obtained by stable isotope analysis.

**Fig 5:**
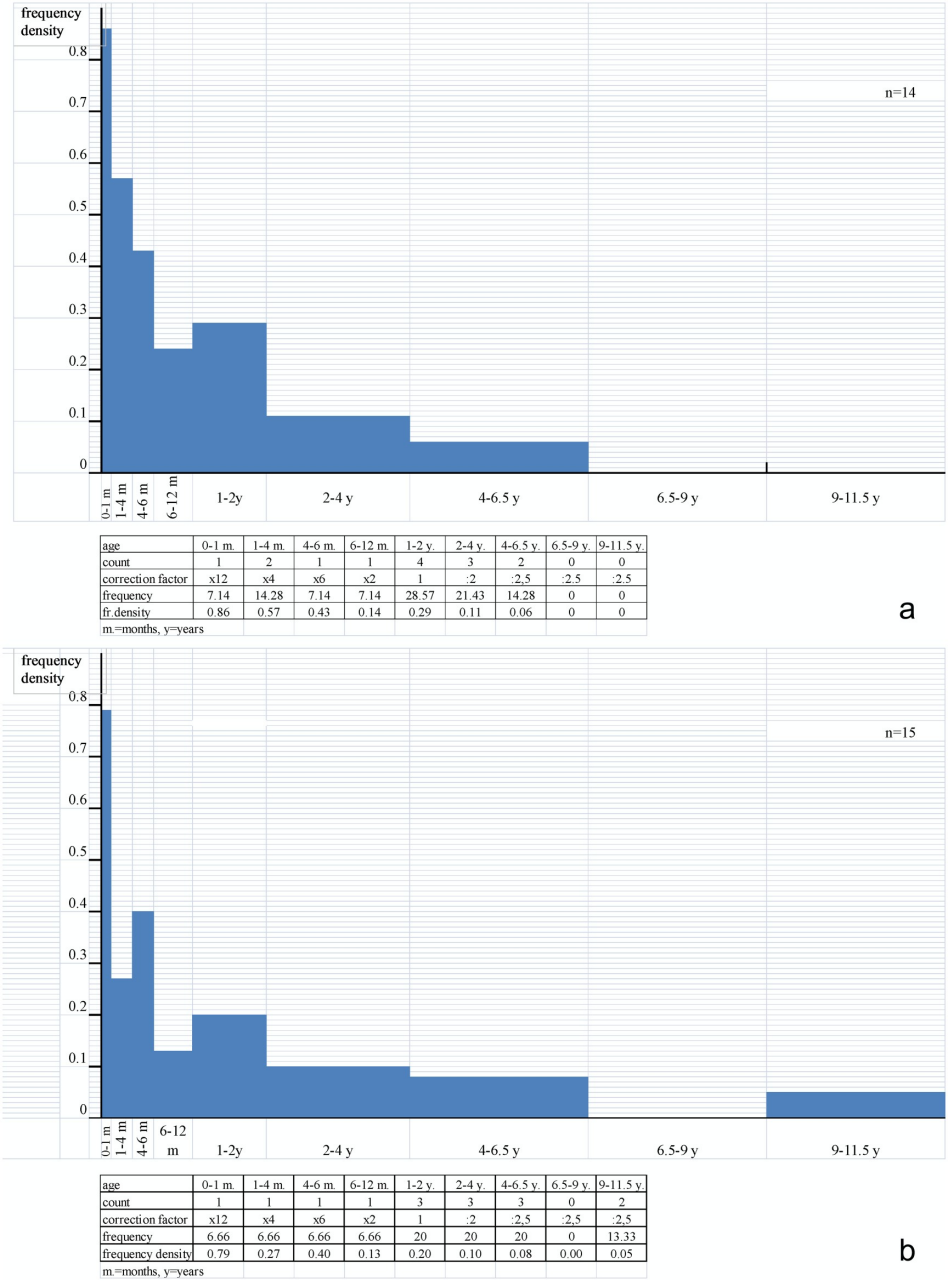
Cattle mortality profiles. a. Starčevo-Grad, b. Magareći Mlin.

The cattle from Starčevo-Grad consisted of individuals <6.5 years old (Fig 5a). The number of individuals who had not reached sexual maturity (five infant and six juvenile animals) significantly exceeds the number of adults (three animals). There were five animals aged <1 year, distributed into four different age classes, possibly related to perinatal mortality and both pre- and post-lactation slaughter. Their presence indicates culling before reaching optimal weight for meat exploitation. The largest number of animals (four), belonging to the age class of 1–2 years, produces a rather significant peak in frequency density and is presumably related to meat exploitation. Among adults, three animals were 2–4 years old, two were 4–6.5 years old, whereas mature and senile animals were completely absent. Given the evidence of ruminant adipose and dairy products in pottery fragments from Starčevo-Grad, the culling profile might be best explained by mixed meat and milk exploitation.

In the cattle assemblage from Magareći Mlin, all age stages except 6.5–9 years are present (Fig 5b). The number of individuals who had not reached sexual maturity (seven infant and juvenile individuals) is close to the number of adults and old adults (eight). There were four animals aged <1 year, each in a different age class, therefore no conclusion could be drawn regarding pre- or post-lactation slaughter. Nevertheless, their presence indicates culling before reaching optimal weight for meat exploitation. Similar to the Starčevo-Grad mortality profile, a significant peak is visible in the age of 1–2 years, presumably related to meat exploitation. The distribution of adults and old adults, probably mainly fertile and lactating females, indicates that stock breeding was not solely oriented towards beef production.

### 2.3. Cattle weaning patterns

Cattle teeth selected for determination of Δ^15^N and ẟ^13^C values of dentine collagen originated from individuals aged 4–6 months (LBMM002 and LBSG060) and 12–15 months (LBMM022).

**Fig 6:**
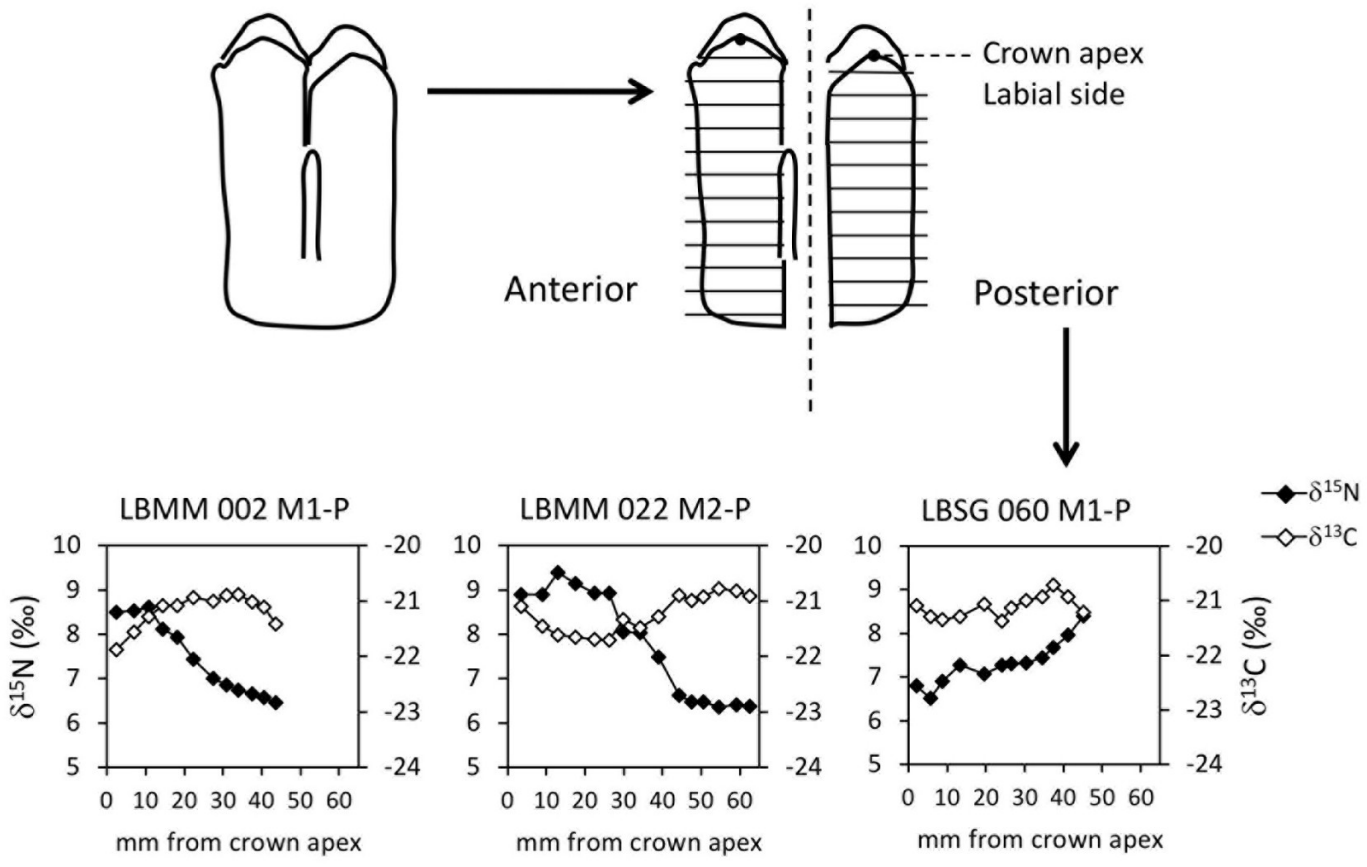
Results from sequential analysis of dentine collagen stable nitrogen (δ^15^N) and carbon (δ^13^C) isotope ratios in cattle first (m1) and second molars (m2) at Magareći Mlin and Starčevo-Grad.

The results from the sequential analysis of δ^15^N and δ^13^C values in dentine collagen are shown in S3 File and on Fig 6. Most collagen extracts meet the criterion for good quality preservation (C and N contents, C/N atomic ratio). The sampling performed on the anterior and posterior lobes of each molar shows great consistency in δ^15^N and δ^13^C values (SI 3), suggesting a good preservation of the original stable isotope ratios. The two cattle teeth from Magareći Mlin (LBMM) show decreasing δ^15^N values from the crown apex (earliest formed) towards the root (latest formed). In LBMM022-m2, δ^15^N values around 9 ‰ over the first 25 mm of crown decrease along tooth crown and stabilize around 6.4 ‰ (Fig 6).

The amplitude of the shift in δ^15^N values (3.1 ‰) is similar to the ^15^N-enrichment between trophic levels (+3–4 ‰, [78,79] and the +3.2–3.6‰ ^15^N-enrichment between cow’s milk and diet [80,81]), likely reflecting the weaning process. The δ^15^N values stabilize in the latest 15 mm dentine formed, suggesting that weaning was completed. In LBMM002-m1, δ^15^N values around 8.5 ‰ over the first 10 mm of the molar crown decrease and reach 6.5 ‰ in the dentine latest formed (Fig 6). Although the δ^15^N values have not stabilized and despite lower intra-tooth amplitude of variation (2 ‰), the sharp decrease in δ^15^N values also suggests a well-advanced weaning process. It may be concluded that both these animals were culled after lactation had substantially decreased or ceased. The cattle tooth from Starčevo-Grad (LBSG060-m1) yielded a different pattern with δ^15^N values increasing from 6.4 ‰ in the crown apex to 8.4 ‰ in the latest formed dentine, a value similar to the highest values measured in LBMM002-m1 and LBMM022-m2 (Fig 6). The weaning process is not recorded in this molar, meaning that this calf died or was culled before its mother’s lactation ended. In a context where milk exploitation was demonstrated by the presence of milk residues in ceramic potsherds, this result is not in favour of the hypothesis of calves being maintained alive until lactation ends in order to stimulate milk let down [30,82,83]. However, this isolated find prevents any conclusion on this matter.

The pattern of increasing δ^15^N values observed in LBSG060-m1 might reflect the shift from *in utero* to sucking diet. In modern cattle, the first molar crown is already partly formed at birth [84]. Yet, although a prenatal signal is expected in the m1 crown, it was not detected in previous studies in modern and archaeological cattle aged 12 months or more [28,29]. This would confirm the hypothesis of the earliest stable isotope ratios in dentine collagen being partly to completely overwritten by signals acquired during subsequent tooth development stages [29]. An important consequence would be that the age at death is an additional factor to the initial timing of tooth formation—and potentially the major factor to be considered—when interpreting stable isotope ratios in dentine collagen in relation to age.

The pronounced difference in the pattern of variation of δ^15^N values between LBMM002-m1 and LBSG060-m1, assigned to the same age class, is enigmatic. It could refer to a radically different developmental story either in the timing of tooth growth or in suckling behaviour. The limited number of teeth sequentially sampled in the present study, and in all previous studies [28–30] prevents a clear understanding of intra-tooth variations in dentine collagen δ^15^N values.

The dentine collagen δ^13^C values vary between -22 ‰ and -20.7 ‰ (Fig 6). These are related to diet and metabolic processes involved in digestion. In prenatal life, the young calf receives carbon derived from its mother’s ruminant digestion. When the calf is born, its own digestive system is similar to a non-ruminant digestive system during the first weeks of life, until the consumption of dry food stimulates the rumen development. These transitions between ruminant/non-ruminant/ruminant digestive systems occur as the first molar develops and are suspected to impact δ^13^C values in enamel bioapatite [85]. However, there is little chance that they would impact collagen δ^13^C values, as a survey of large ruminant versus non-ruminant wild herbivores has shown that differences in digestive strategy have no influence on the response of collagen δ^13^C values to diet [86]. Consequently, the variations observed in δ^13^C values along the crown of these cattle m1 and m2 are likely to directly reflect changes in the δ^13^C values of diet. Considering a 5 ‰^13^C-enrichment between the protein fraction of diet and collagen [87], they refer to plant values around -27 ‰ to -25.7 ‰, typical for C_3_ plants in an open environment. Seasonal variations of 1 ‰ to 2 ‰ in the δ^13^C values of C_3_ plants have been reported, with the highest values occurring during the summer and the lowest during the winter [88]. Herbivores feeding on these plants inherit these seasonal variations, which are commonly observed in sequential δ^13^C values measured in enamel (see for example [89]). The changes in δ^13^C values in the dentine collagen of the Magareći Mlin and Starčevo-Grad cattle might well reflect seasonal variations. If this assumption is correct, all three cattle recorded a late summer/autumn signal in the last dentine formed, meaning that they died at or shortly after this time of the year.

### 2.4. Dental calculus proteins

**Fig 7:**
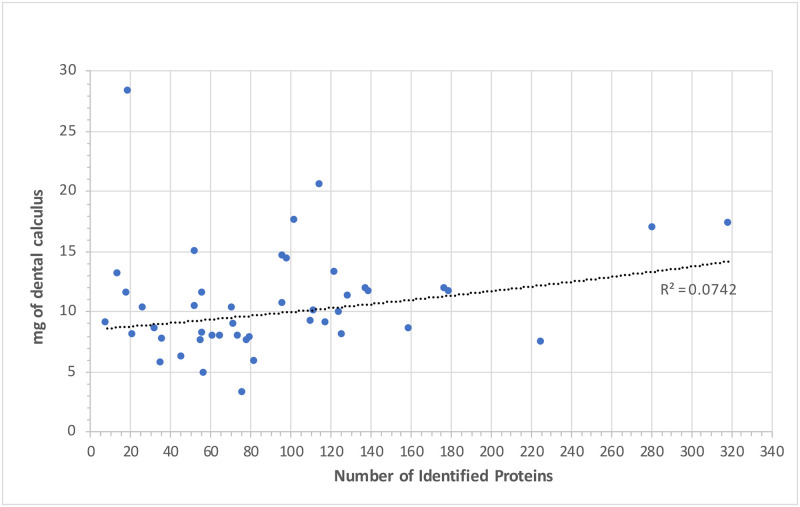
Relationship between quantity (mg) of starting material and number of identified proteins.

Proteins were recovered from all dental calculus samples, although total protein identifications varied substantially across the dataset, ranging from a minimum of 8 to a maximum of 319 (mean = 93.5 median = 78, considering only ‘supernatant & pellet’ extractions) (see S4.2 Table in S4 and S5 Files). The overall protein recovery in this study was low, when compared to previous dental calculus studies applying a similar protein extraction technique. For example, Hendy *et al*. [32] identified an average of 400 proteins from the dental calculus of post-medieval skeletons from Britain, while Mays *et al*. [90] identified an average of 140 proteins from Middle Neolithic and Middle Bronze age skeletons from the site of Stonehenge. The relatively low quantity of recovered proteins is likely not related to the amount of starting material used for analysis, as we detected no correlations between the quantity (mg) of dental calculus analysed and the total number of identified proteins within that sample (Fig 7), consistent with observations by Mackie *et al*. [91] and Hendy *et al*. [32].

**Fig 8:**
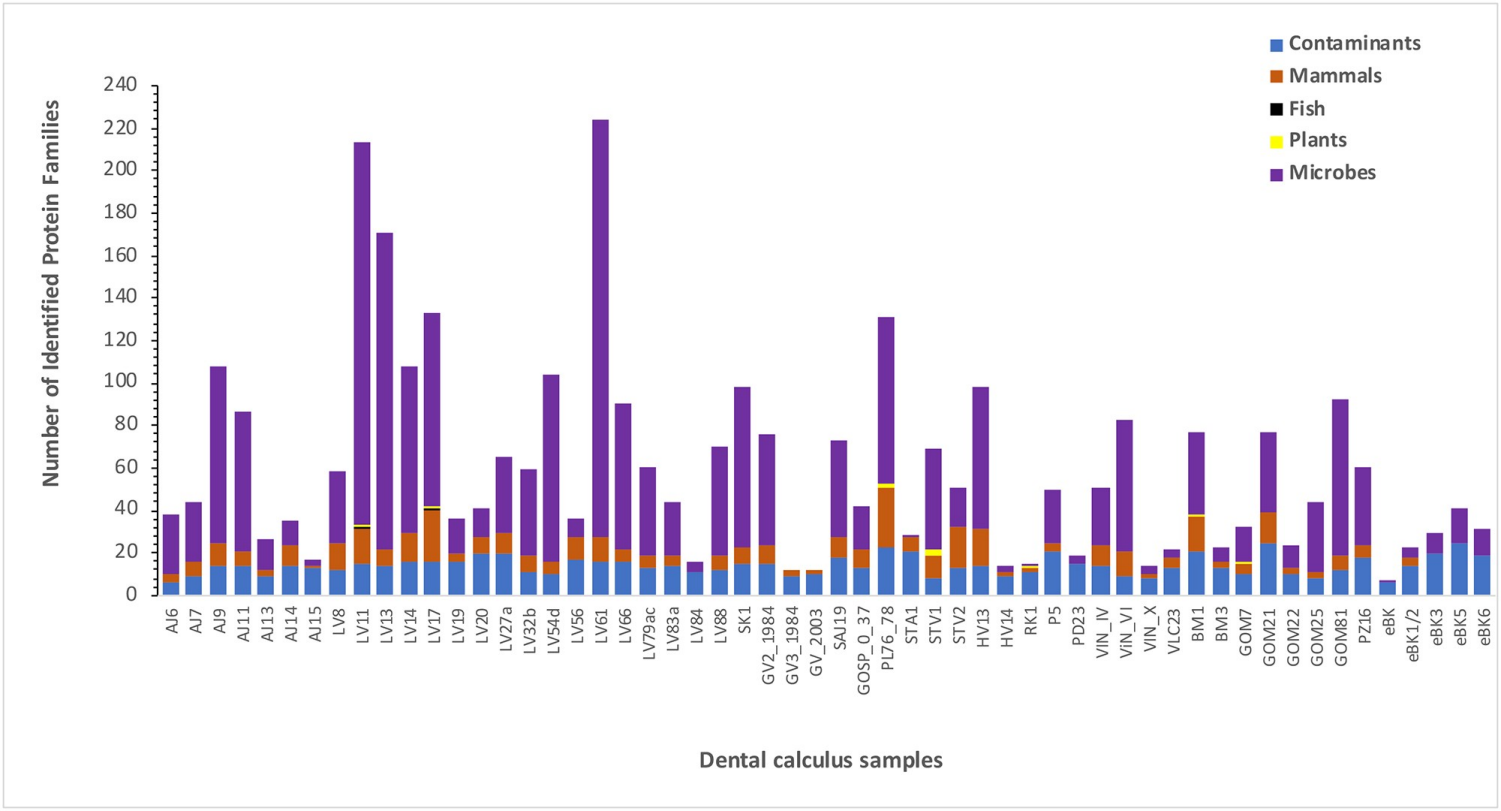
Proportion of contaminant, mammalian, plant, fish, and microbial protein families identified in the dental calculus samples. Many samples are marked by a high proportion of contaminants (predominantly keratins), which could be due to sample handling or general low abundance of endogenous proteins.

Microbial proteins accounted for the majority of the identified protein families, ranging from 0–88% of the identified protein families (mean 56%) (Fig 8), consistent with the fact that dental calculus is a microbial biofilm. Mammalian proteins, the majority of which were assigned to the human host, accounted for 5–28% of the identified protein families, similar to the proportion of mammalian proteins observed in Hendy *et al*. [32].

**Fig 9:**
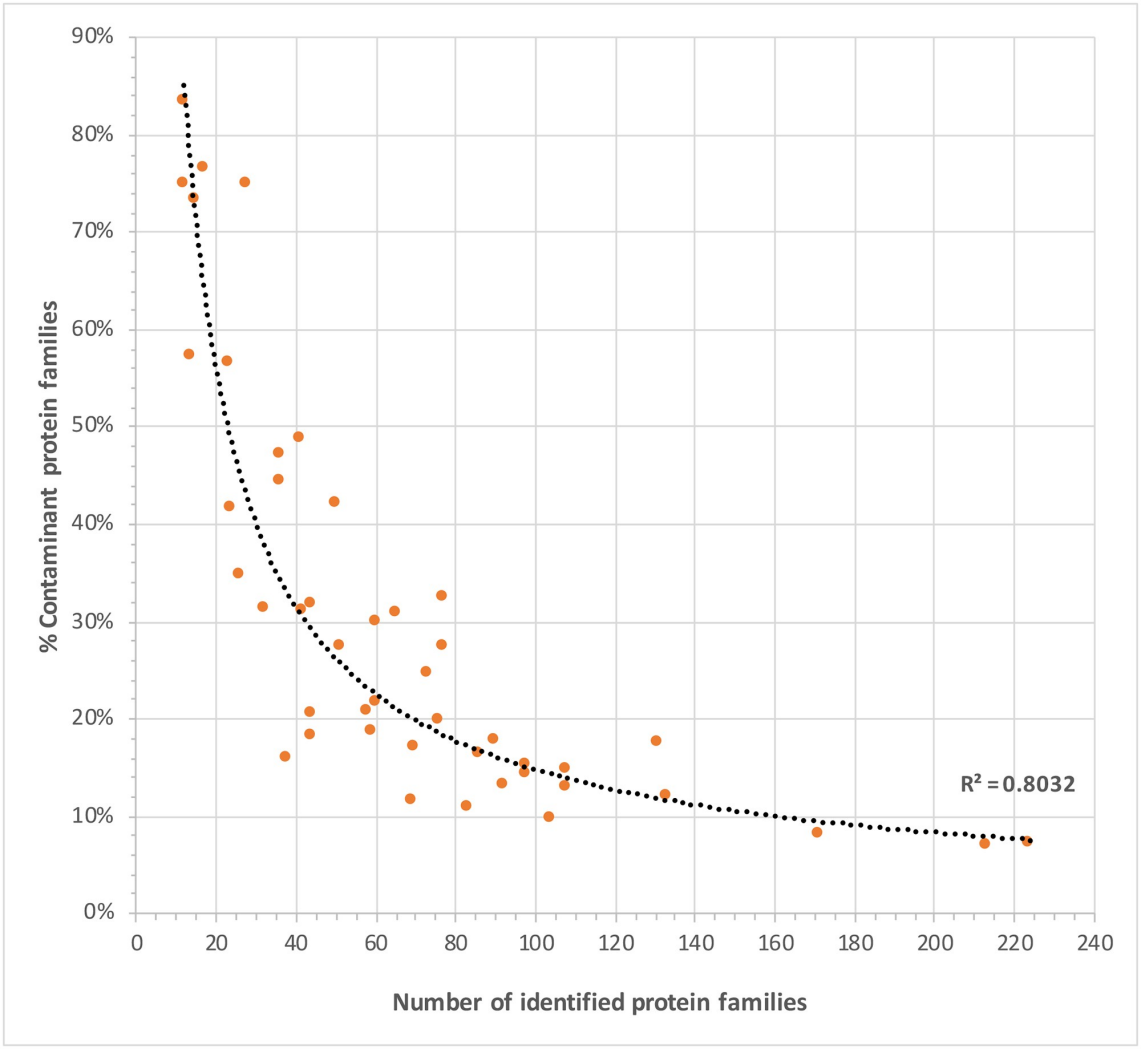
Scatterplot displaying the correlation between the total number of identified protein families and the proportion of putative contaminant proteins. Samples with relatively low numbers of proteins overall are dominated by contaminants (predominantly keratins), which could be due to sample handling or general low abundance of endogenous proteins.

Any proteins reported as contaminants in previous dental calculus studies [32,92] and/or detected in any extraction or instrument blanks were considered potential laboratory contaminants. Overall, the calculus proteomes in this study demonstrated an unexpectedly high proportion of contaminant proteins, representing 7–83% of the identified protein families (mean 30%). The proportion of putative contaminants was particularly high in samples with low numbers of identified proteins families overall (i.e., samples with fewer than 80–100 protein families) (Fig 9). Proteins derived from human skin (e.g., keratin, collagen, hornerin) were particularly abundant across the dataset, potentially resulting from previous handling of the skeletal remains, and/or a lack of preserved endogenous ancient proteins within the calculus. Although 8 individuals displayed evidence of putative plant and fish dietary proteins (S4.3 Table in S4 File), none of the individuals displayed confident proteomic evidence for milk consumption.

## 3. Discussion

### 3.1. The importance of milk and dairying in the northern Balkans

**Fig 10:**
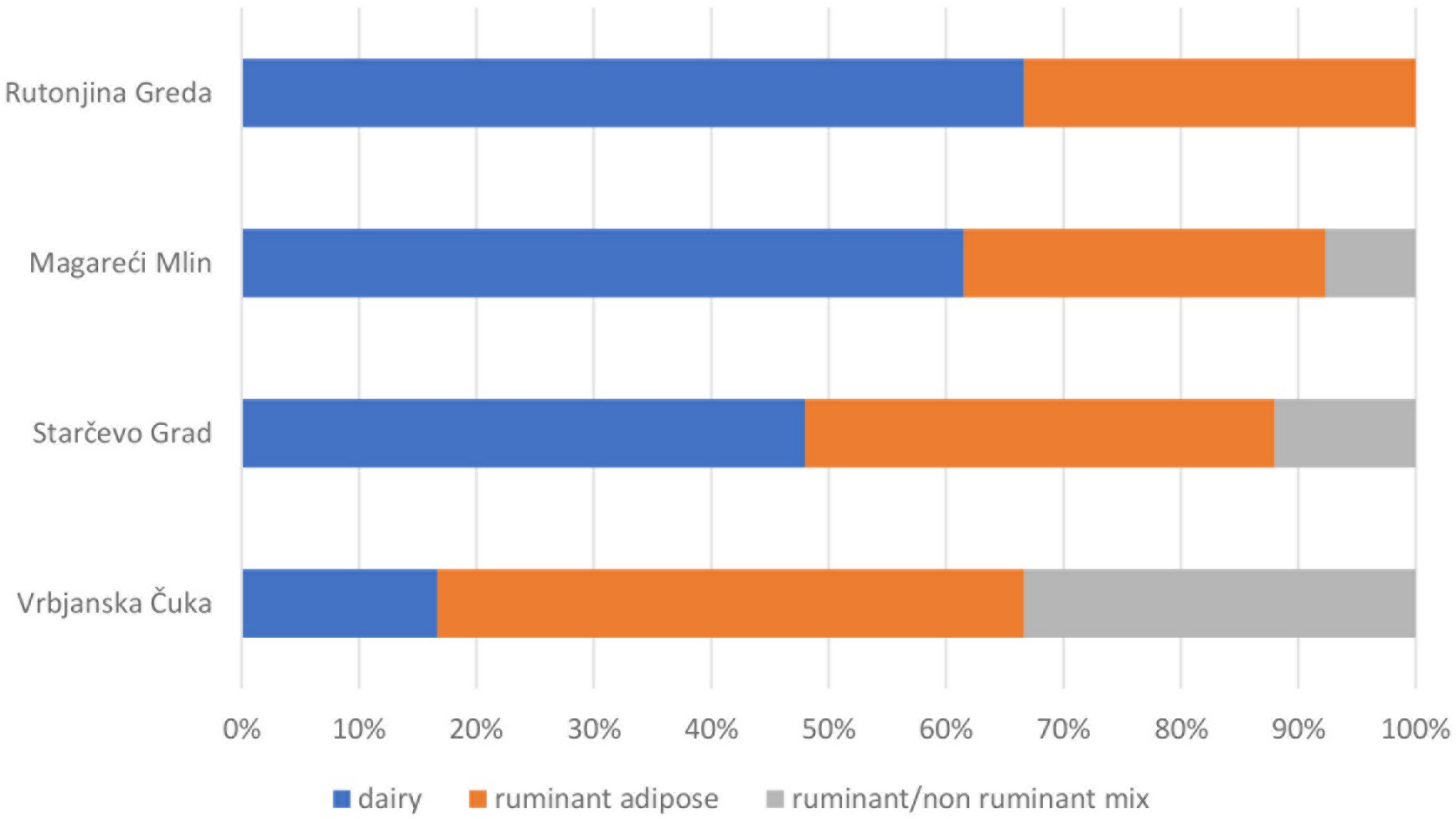
Relative abundances of different food categories of terrestrial animal origin, as seen through organic residue analyses of pottery vessels.

Within the Balkan Peninsula, it is clear that milk and dairying practices become more commonplace as farming spreads from South to North. On one hand, even though present, dairy fats are less frequent at the Neolithic sites of Greece [20,25], which correlates well with central and eastern Anatolia [25,93]. On the other, pottery containing dairy products are much more common at northern Balkan sites [16,18,38]. Our results fit well in this general pattern of the relative importance of dairying in the region. At the southernmost site of Vrbjanska Čuka, not far from the Macedonian-Greek border, only one of the six pottery vessels that contained animal fats was used for dairying, whereas the remaining five pots were used for animal carcass product processing. In the Pannonian plain, at the Starčevo-Grad site 12 (48%) of the lipid-yielding vessels contained dairy lipids, while at Magareći Mlin, 8 (62%) of the lipid-yielding vessels were used for dairy product processing. At Rutonjina Greda, two of the three lipid-bearing sherds yielded dairy lipids (Fig 10). The processing of milk clearly became an important activity within the subsistence base of the farming communities of the temperate Neolithic in the Balkans, although possibly varying in intensity between different communities. Dairying has already been suggested by Ethier *et al*. [16] (see also [17,18,25]) as a strategy of early farmers for exploiting an important resource rich in protein and fats, in a challenging continental environment where the Mediterranean suite of domesticates had to be modified. In addition, some traditional dairying practices significantly decrease the lactose content [94–96], and the short-lived milk is converted into a durable and storable product.

In a wider geographic perspective however, this pattern should be considered with caution, as there are significant regional exceptions. The most striking is the evidence from NW Anatolia (the Marmara region of Turkey), where the overwhelming majority of the pottery contained dairy fats and there was also evidence of prolonged heat processing of milk [25,97]. Interestingly, these sites also had higher proportions of cattle bones in the faunal assemblage than the surrounding regions. Not as pronounced, but significant percentages of dairy lipids were retrieved from the western Mediterranean as well [38,98]. At the same time, it seems that the first LBK (Linearbandkeramik) farmers of the continental regions of central Europe did not rely significantly on dairying [99,100]. Clearly, there are a number of different factors which contribute to shaping early farming economies. Through the studies so far, a highly varied picture emerges, suggesting a more geographically focused, regional approach is best suited for future research.

Returning to the importance of milk, the metaproteomic analyses of dental calculus samples from the same northern Balkan areas did not provide any evidence of consumption of fresh, unprocessed milk. ß-lactoglobulin, a protein found within the whey fraction of milk, is one of the most common dietary proteins observed within previous analyses of archaeological dental calculus [31,32,101,102]. Because it is exclusively found in milk it is a specific biomarker for this product, and it also appears to be a particularly robust protein and is more resistant to enzymatic degradation and microbial proteolysis than other milk proteins [103].

Neither ß-lactoglobulin nor any other milk proteins could be confidently detected within this dataset. Although skeleton SAJ19 displayed a single peptide matching to bovine ß-lactoglobulin, and skeleton LV17 displayed a single peptide matching to Alpha-S1-casein (S4.3 Table in S4 File), these identifications do not meet the criteria of having two or more distinct, and confidently identified peptides, criteria necessary to reduce the likelihood of misidentifications and false positive results [104–106]. Considering that ß-lactoglobulin is the dominant protein in the whey fraction of milk, the lack of ß-lactoglobulin in the dental calculus samples cannot be simply explained with the overall poor preservation of proteins. ß-lactoglobulin appears to be a relatively robust protein and has been identified in other Bronze Age and Neolithic samples, where total protein identifications were similar to those observed in this study. For example, even in relatively poorly preserved early Neolithic dental calculus samples from Britain (with total protein identifications ranging from 15 to 128 proteins), ß-lactoglobulin was detected in six of the 10 tested individuals [107].

The lack of direct evidence for milk consumption through our dental calculus protein analyses, however, should not be used in support of a straightforward claim that milk was not used in a raw, liquid form. Further studies are needed on a larger group of samples from the region, as well as an assessment of the degradation processes of the milk proteins in the specific post-depositional and post-excavation conditions. At present, we cannot reject the hypothesis that humans consumed fresh, unprocessed milk (or the whey fraction and related products), based solely on our dental calculus results. Animal milk could have served as a substitute for breastmilk in the diet of the youngest members of the community, as baby weaning food. The emergence of new foodstuffs (i.e. milk and ground cereals) would have provided Neolithic mothers (as well as other members of the community) with novel options for feeding infants and small children prior to introducing solids in their diet. Such baby gruels were possibly served by bone spoons made from cattle metapodial bones, artefacts ubiquitous in the Neolithic Anatolia and the Balkans, as a recent study of bone spoons from the site of Starčevo-Grad has confirmed [108]. These utensils bore numerous traces of use and damage which corresponded to milk teeth marks, likely produced by children during feeding and/or chewing.

### 3.2. Animal husbandry strategies, cattle weaning and possible implications for milk exploitation

Archaeozoological analyses of faunal samples from a number of Early Neolithic sites in the Balkans, including those from Vrbjanska Čuka, Starčevo-Grad and Magareći Mlin discussed in this paper, indicate that animal husbandry (and to a lesser degree hunting, fishing and shellfish collection) represented the main economic activities. Similar to the lipid residue evidence, there seem to have been pronounced regional differences in animal husbandry practices, mainly between the southern parts of the Balkan Peninsula, and its central and northern parts, bordering with the Great Pannonian plain. In the former, herding strategies were mainly oriented towards ovicaprids, whereas in the latter, while ovicaprids remain important, a significantly higher proportion of domestic cattle is documented [16,27]. The greater reliance on cattle in the temperate Balkans Neolithic corresponds to the increased presence of dairy fats in the northern parts of the peninsula, which suggests that dairying most likely involved cow’s milk (although milk from other ruminants should not be excluded), which resonates well with the evidence from the Marmara region [25]. Furthermore, cattle mortality profiles from Starčevo-Grad and Magareći Mlin are indicative of culling strategies oriented towards both meat and milk exploitation. Isotopic analyses of cattle dentine collagen indicate that two calf individuals from Magareći Mlin (from age classes 4–6 months and 1–2 years) were kept alive until the end of lactation. If a similar pattern is to be assumed for the herd as a whole, this implies that the herders at Magareći Mlin had to share milk production with the calves. On the other hand, at Starčevo-Grad, a single calf individual (from age class 4–6 months) was not weaned at the time of death. It remains difficult to interpret these discrepancies given the paucity of the sample, but they could be indicative of different culling strategies at the two sites, or perhaps of different treatments of individual animals and different circumstances related to slaughter events.

### 3.3. Subsistence diversity in early farming communities (processing of fish and cereals, and beeswax utilization)

Finding proof for dairying in the Balkans is important because milk is associated with early domestic animals, and therefore with the early farming economy. However, the subsistence base of the Neolithic people was far more diverse. At two of the sites (Vrbjanska Čuka and Starčevo-Grad), in five vessels, we were able to identify compounds indicative of cooking plants. Two of them did not contain traces of animal product lipids, which suggests that they were used exclusively for processing plant-based food.

Beeswax was identified in vessels at sites in the temperate northern zone, near the Pannonian plain (Starčevo-Grad and Magareći Mlin). Although beeswax could only be unambiguously identified in four vessels, it seems that four of the likely six pots displaying beeswax residue were mixed with animal products, likely dairy. The other two contained only beeswax. None of these six vessels contained carcass animal fats. It is possible that beeswax was used as a technical solution in the pottery making process, to reduce vessel permeability, but if that was the case, we would expect to identify beeswax in a much larger portion of the assemblage. It is more likely that we are detecting honey as part of the diet. The size of our sample prevents us from suggesting culinary practices or dietary preferences but combining data from a larger assemblage from a geographically constrained location could be an interesting line of investigation.

In our study, we could not detect a firm evidence for aquatic resources on a molecular level, despite the proximity of three of the four sites to the Danube. Thus, the lipid analyses suggest a diet based mainly on terrestrial resources, which corresponds well with the data gathered for the early Neolithic of the wider region [16,17,22]. So far, the only exception from this pattern in the Balkans are the well-known sites from the Iron Gates (less than 100 km SE from Starčevo-Grad), where aquatic products played dominant role in the human diet. This was confirmed not only by organic residue pottery analyses [22], but also by isotopic analyses on human remains [19,109–111] and archaeozoological studies [112–114]. Such a diversity in the human subsistence between the specialized fishing communities in the gorges on one side, and the farming villages in the plains on the other reflects the complex dynamics behind the neolithisation process in this specific area, where the interaction between immigrating farmers and local hunter-gatherer-fishers, and the availability of arable land played crucial roles [19,111,115]. The presence of hunter-gatherer-fishers outside of the gorges is still under investigation.

## 4. Conclusions

These combined results from bioarchaeological investigations of pottery and human and animal remains provided new information about the early Neolithic diet in the Balkans. Mortality profiles of domestic animals, established through taxonomic assessment of animal bones, as well as isotopic analyses of cattle teeth targeting the detection of weaning patterns, have suggested a mixed stock-herding strategy, i.e. exploitation of both meat and milk. The exploitation of milk was evidenced directly with the identification of dairy fat molecules preserved in the Neolithic pottery. The authenticity of the lipid residues was further confirmed by direct compound-specific radiocarbon dating of the dairy lipids, confirming their Neolithic origin. The presence of dairy residues in the pottery vessels suggests that dairying was a common practice at the onset of farming in the Balkans. It becomes obvious however, that dairying did not have the same importance for the people living in different climatic and environmental conditions. Even though practiced in the Sub-Mediterranean regions, dairying gained more importance in the colder Continental North. The heat treatment (in pottery vessels) during dairying facilitates the separation of the whey fraction (with the lactose and the soluble proteins) from the fatty fraction and insoluble proteins, such as caseins. The whey was probably not used in adult diet, while the fatty curd was transformed into durable dairy products and consumed. The proteomic analyses of dental calculus from humans could not confirm that raw milk (i.e. fresh, unprocessed, whole milk) was also part of the diet. Due to uncertainties surrounding the taphonomy of dental calculus proteins however, the absence of milk proteins in our samples is insufficient to reject the idea of milk consumption. Our residue analyses strongly suggest milk was part of human subsistence and could quite conceivably have been used during the weaning period of babies and young children.

The exploitation and inclusion of different kinds of domesticates in the diet, namely plants, was also confirmed by molecular traces in the pottery residue. This is not surprising, since other evidence, such as carbonized grains, wheat ear imprints, or sickle blades are common finds at Neolithic sites in the region. Nevertheless, this is further direct, unambiguous evidence that plants, including cereals, were cooked as food. Beeswax (honey) traces were also found preserved within the pottery matrix. Finding virtually no trace of aquatic resources in our residue analyses, we conclude that at least as far as cooking in pottery vessels are concerned, the subsistence at these four sites was firmly based on terrestrial resources.

## Supporting information

S1 File Organic residue results main table. (DOCX)

S2 File Graphic representation of pottery samples. (DOCX)

S3 File Dentine collagen results. (DOCX)

S4 File Dental calculus tables. (DOCX)

S5 File Materials and methods. (DOCX)

S6 File Information on proteomic files uploaded to the MaSSIVE repository. (XLSX)
