# Supplementary material for: Living off the land: Terrestrial-based diet and dairying in the farming communities of the Neolithic Balkans
Source: PLoS One. 2020 Aug 20;15(8):e0237608. doi: 10.1371/journal.pone.0237608 (PMC7444498; doi:10.1371/journal.pone.0237608)
Supplement: S1 File — (DOCX) [file pone.0237608.s001.docx]

The importance of stock herding and dairying in the Neolithic Balkans and the subsistence diversity

Supplementary Information 1: Organic residue results main table

Darko Stojanovski, Ivana Živaljević, Vesna Dimitrijević, Julie Dunne, Richard P. Evershed, Marie Balasse, Adam Dowle, Jessica Hendy, Krista McGrath, Roman Fischer, Camilla Speller, Jelena Jovanović, Emmanuelle Casanova, Timothy Knowles, Lidija Balj, Goce Naumov, Anđelka Putica, Andrej Starović, Sofija Stefanović

Table S1.1 Summary of the samples submitted for organic residue extraction and analyses, and the obtained results

| Sample | Site | Vessel form | Part sampled | Lipid  concentration  (μg/g) | δ^13^C  (‰) | | Δ^13^C (‰)  (δ^13^C_18:0 -_ δ^13^C_16:0_) | Animal fats | Additional assignment |
| --- | --- | --- | --- | --- | --- | --- | --- | --- | --- |
|  |  |  |  |  | C_16:0_ | C_18:0_ |  |  |  |
| VC01 | Vrbjanska Čuka | 310 | rim | 46 | -27.5 | -27.8 | -0.3 | ruminant adipose/ Mixture ruminant/non-ruminant | / |
| VC02 | Vrbjanska Čuka | 420 | rim | 12 | / | / | / | / | plant |
| VC03 | Vrbjanska Čuka | 420 | rim | / | / | / | / | / | / |
| VC04 | Vrbjanska Čuka | 440 | rim | / | / | / | / | / | / |
| VC05 | Vrbjanska Čuka | 310 | rim | / | / | / | / | / | / |
| VC06 | Vrbjanska Čuka | 430 | rim | / | / | / | / | / | / |
| VC07 | Vrbjanska Čuka | 330 | rim/body | / | / | / | / | / | / |
| VC08 | Vrbjanska Čuka | 220 | rim | / | / | / | / | / | / |
| VC09 | Vrbjanska Čuka | 320 | rim | 198 | -26.6 | -29.4 | -2.9 | ruminant adipose | plant |
| VC10 | Vrbjanska Čuka | 220 | rim/body | 23 | -26.1 | -28.4 | -2.3 | ruminant adipose | plant |
| VC11 | Vrbjanska Čuka | 210 | rim/body | / | / | / | / | / | / |
| VC12 | Vrbjanska Čuka | 440 | rim | / | / | / | / | / | / |
| VC13 | Vrbjanska Čuka | 330 | rim/neck | / | / | / | / | / | / |
| VC14 | Vrbjanska Čuka | N/A | base/body | / | / | / | / | / | / |
| VC15 | Vrbjanska Čuka | N/A | body | / | / | / | / | / | / |
| VC16 | Vrbjanska Čuka | 220 | rim | / | / | / | / | / | / |
| VC17 | Vrbjanska Čuka | 210 | rim | 8 | -26.5 | -26.8 | -0.3 | ruminant adipose/ Mixture ruminant/non-ruminant | / |
| VC18 | Vrbjanska Čuka | 320 | rim | / | / | / | / | / | / |
| VC19 | Vrbjanska Čuka | 440 | rim | 12 | -24.4 | -26.2 | -1.7 | ruminant adipose | / |
| VC20 | Vrbjanska Čuka | 430 | rim | / | / | / | / | / | / |
| VC21 | Vrbjanska Čuka | 220 | rim/body | / | / | / | / | / | / |
| VC22 | Vrbjanska Čuka | 330 | rim/body | / | / | / | / | / | / |
| VC23 | Vrbjanska Čuka | 210 | rim | / | / | / | / | / | / |
| VC24 | Vrbjanska Čuka | 210 | rim | 11 | / | / | / | / | / |
| VC25 | Vrbjanska Čuka | 400 | body/handle | / | / | / | / | / | / |
| VC26 | Vrbjanska Čuka | 330 | rim/body | 410 | -25.9 | -30.8 | -4.8 | dairy | / |
| VC27 | Vrbjanska Čuka | 330 | rim/body | / | / | / | / | / | / |
| ST28 | Starčevo Grad | N/A | body | / | / | / | / | / | / |
| ST29 | Starčevo Grad | N/A | body | 278 | -28 | -30.9 | -2.8 | ruminant adipose | / |
| ST30 | Starčevo Grad | 500 | leg/platform | / | / | / | / | / | / |
| ST31 | Starčevo Grad | N/A | body | not analysed | | | | | |
| ST32 | Starčevo Grad | 210 | rim | / | / | / | / | / | / |
| ST33 | Starčevo Grad | 430 | rim/body | / | / | / | / | / | / |
| ST34 | Starčevo Grad | 430 | rim/body | 10 | / | / | / | / | / |
| ST35 | Starčevo Grad | 220 | rim | 15 | -28 | -28.2 | -0.2 | ruminant adipose/ Mixture ruminant/non-ruminant | / |
| ST36 | Starčevo Grad | 400 | rim/body | 26 | / | / | / | / | / |
| ST37 | Starčevo Grad | 420 | rim | / | / | / | / | / | / |
| ST38 | Starčevo Grad | 210 | rim | / | / | / | / | / | / |
| ST39 | Starčevo Grad | 430 | rim | / | / | / | / | / | / |
| ST40 | Starčevo Grad | 200 | base/body | / | / | / | / | / | / |
| ST41 | Starčevo Grad | 430 | rim/body | 8 | -26.5 | -31.8 | -5.4 | dairy | / |
| ST42 | Starčevo Grad | 420 | rim/body | 5 | -27.8 | -31.7 | -3.9 | dairy | / |
| ST43 | Starčevo Grad | 220 | rim | 23 | -27 | -30.7 | -3.7 | dairy | / |
| ST44 | Starčevo Grad | 410 | rim/body | 58 | -27.7 | -32.8 | -5.1 | dairy | / |
| ST45 | Starčevo Grad | 210 | rim/body | / | / | / | / | / | / |
| ST46 | Starčevo Grad | 210 | rim/body | / | / | / | / | / | / |
| ST47 | Starčevo Grad | 430 | rim | / | / | / | / | / | / |
| ST48 | Starčevo Grad | 210 | rim | / | / | / | / | / | / |
| ST49 | Starčevo Grad | 430 | rim/body | / | / | / | / | / | / |
| ST50 | Starčevo Grad | 420 | rim/body | / | / | / | / | / | / |
| ST51 | Starčevo Grad | 210 | rim/body | / | / | / | / | / | / |
| ST52 | Starčevo Grad | 410 | rim | / | / | / | / | / | / |
| ST53 | Starčevo Grad | 220 | rim | / | / | / | / | / | / |
| ST54 | Starčevo Grad | 210 | rim | 138 | -27.6 | -31.3 | -3.7 | dairy* | beeswax |
| ST55 | Starčevo Grad | N/A | body | 120 | -25.9 | -31.2 | -5.4 | dairy* | beeswax |
| ST56 | Starčevo Grad | 320 | rim | 751 | -25.8 | -26.6 | -0.8 | ruminant adipose/ Mixture ruminant/non-ruminant | / |
| ST57 | Starčevo Grad | 430 | rim/body | 67 | -28.7 | -31.3 | -2.6 | ruminant adipose | / |
| ST58 | Starčevo Grad | 220 | rim | / | / | / | / | / | / |
| ST59 | Starčevo Grad | 410 | rim | / | / | / | / | / | / |
| ST60 | Starčevo Grad | N/A | body | 53 | -27.5 | -29.5 | -2 | ruminant adipose | / |
| ST61 | Starčevo Grad | 320 | rim | / | / | / | / | / | / |
| ST62 | Starčevo Grad | N/A | body | / | / | / | / | / | / |
| ST63 | Starčevo Grad | N/A | body | / | / | / | / | / | / |
| ST64 | Starčevo Grad | 420 | rim/body | / | / | / | / | / | / |
| ST65 | Starčevo Grad | 420 | rim/body | / | / | / | / | / | / |
| ST66 | Starčevo Grad | 220 | rim | / | / | / | / | / | / |
| ST67 | Starčevo Grad | 410 | rim | / | / | / | / | / | / |
| ST68 | Starčevo Grad | 220 | rim | / | / | / | / | / | / |
| ST69 | Starčevo Grad | 220 | rim | / | / | / | / | / | / |
| ST70 | Starčevo Grad | N/A | rim | 501 | -28.6 | -33.2 | -4.6 | dairy | / |
| ST71 | Starčevo Grad | 220 | rim | / | / | / | / | / | / |
| ST72 | Starčevo Grad | 310 | rim | / | / | / | / | / | / |
| ST73 | Starčevo Grad | 210 | rim/body | 14 | / | / | / | / | plant |
| ST74 | Starčevo Grad | 220 | rim | / | / | / | / | / | / |
| ST75 | Starčevo Grad | 430 | rim | 6 | -26.5 | -31.7 | -5.2 | dairy | / |
| ST76 | Starčevo Grad | 430 | rim/body | 92 | -28 | -32.7 | -4.7 | dairy | / |
| ST77 | Starčevo Grad | 420 | rim/body | 18 | -27.1 | -29.4 | -2.4 | ruminant adipose | / |
| ST78 | Starčevo Grad | N/A | body | 258 | -28.4 | -31.5 | -3.1 | ruminant adipose | / |
| ST79 | Starčevo Grad | N/A | base/body | / | / | / | / | / | / |
| ST80 | Starčevo Grad | N/A | body | / | / | / | / | / | / |
| ST81 | Starčevo Grad | 310 | rim | / | / | / | / | / | / |
| ST82 | Starčevo Grad | 330 | rim | 209 | -26.3 | -31.3 | -5 | dairy* | beeswax |
| ST83 | Starčevo Grad | 220 | rim | 10 | -27.5 | -31.6 | -4 | dairy | plant |
| ST84 | Starčevo Grad | 220 | rim | / | / | / | / | / | / |
| ST85 | Starčevo Grad | 430 | rim/body | / | / | / | / | / | / |
| ST86 | Starčevo Grad | 430 | rim/body | 8 | -27.6 | -33.3 | -5.6 | dairy | / |
| ST87 | Starčevo Grad | 210 | rim/body | 9 | -32.6 | -33 | -0.4 | ruminant adipose/ Mixture ruminant/non-ruminant | / |
| ST88 | Starčevo Grad | 420 | rim/body | 20 | -27.5 | -30.7 | -3.3 | dairy | / |
| ST89 | Starčevo Grad | 210 | rim/body | / | / | / | / | / | / |
| ST90 | Starčevo Grad | 320 | rim | 160 | / | / | / | / | beeswax |
| ST91 | Starčevo Grad | 430 | rim/body | / | / | / | / | / | / |
| ST92 | Starčevo Grad | 310 | rim | / | / | / | / | / | / |
| ST93 | Starčevo Grad | 210 | rim/body | / | / | / | / | / | / |
| ST94 | Starčevo Grad | 330 | rim/body | / | / | / | / | / | / |
| ST95 | Starčevo Grad | 310 | rim/body | / | / | / | / | / | / |
| ST96 | Starčevo Grad | 310 | rim/body | / | / | / | / | / | / |
| ST97 | Starčevo Grad | 220 | rim | / | / | / | / | / | / |
| ST98 | Starčevo Grad | 420 | rim/body | 171 | -29.9 | -31.5 | -1.7 | ruminant adipose | / |
| ST99 | Starčevo Grad | 440 | rim/body | 40 | -27.8 | -30.8 | -3 | ruminant adipose | / |
| ST100 | Starčevo Grad | 430 | rim/body | 115 | -28.9 | -31.5 | -2.7 | ruminant adipose | / |
| ST101 | Starčevo Grad | 220 | rim | / | / | / | / | / | / |
| ST102 | Starčevo Grad | N/A | refitted to ST93 | | | | | | |
| ST103 | Starčevo Grad | N/A | refitted to ST93 | | | | | | |
| ST104 | Starčevo Grad | N/A | body | / | / | / | / | / | / |
| ST105 | Starčevo Grad | 310 | rim | / | / | / | / | / | / |
| ST106 | Starčevo Grad | 420 | neck | / | / | / | / | / | / |
| ST107 | Starčevo Grad | 420 | rim/body | / | / | / | / | / | / |
| ST108 | Starčevo Grad | 420 | rim/body | / | / | / | / | / | / |
| ST109 | Starčevo Grad | 220 | rim/body | 14 | -27.8 | -32 | -4.1 | dairy | / |
| ST110 | Starčevo Grad | 430 | rim | / | / | / | / | / | / |
| ST111 | Starčevo Grad | 430 | rim/body | / | / | / | / | / | / |
| ST112 | Starčevo Grad | 320 | rim/body | / | / | / | / | / | / |
| ST113 | Starčevo Grad | 210 | rim | / | / | / | / | / | / |
| ST114 | Starčevo Grad | 220 | rim | / | / | / | / | / | / |
| ST115 | Starčevo Grad | 430 | rim | / | / | / | / | / | / |
| ST116 | Starčevo Grad | 430 | rim/body | / | / | / | / | / | / |
| ST117 | Starčevo Grad | 230 | rim/body | / | / | / | / | / | / |
| ST118 | Starčevo Grad | N/A | body | 1920 | -27.2 | -33 | -5.7 | dairy | / |
| ST119 | Starčevo Grad | 210 | rim/body | / | / | / | / | / | / |
| ST120 | Starčevo Grad | 420 | rim/body | 16 | -27 | -28.1 | -1.1 | ruminant adipose | / |
| ST121 | Starčevo Grad | 430 | rim/body | 348 | -27.1 | -28.5 | -1.4 | ruminant adipose | / |
| ST122 | Starčevo Grad | 210 | rim/body | / | / | / | / | / | / |
| ST123 | Starčevo Grad | N/A | body | / | / | / | / | / | / |
| MM124 | Magareći Mlin | 500 | rim | / | / | / | / | / | / |
| MM125 | Magareći Mlin | 500 | rim/leg | / | / | / | / | / | / |
| MM126 | Magareći Mlin | 500 | platform/leg | / | / | / | / | / | / |
| MM127 | Magareći Mlin | 440 | rim/neck | / | / | / | / | / | / |
| MM128 | Magareći Mlin | 430 | rim/neck | / | / | / | / | / | / |
| MM129 | Magareći Mlin | N/A | rim | / | / | / | / | / | / |
| MM130 | Magareći Mlin | 210 | rim/body | / | / | / | / | / | / |
| MM131 | Magareći Mlin | 220 | rim/body | / | / | / | / | / | / |
| MM132 | Magareći Mlin | 220 | rim/body | 78 | -27.9 | -32.7 | -4.8 | dairy | / |
| MM133 | Magareći Mlin | 310 | rim/body | 296 | -26.4 | -30.5 | -4.1 | dairy | / |
| MM134 | Magareći Mlin | 430 | rim/neck | 29 | -26.6 | -32.5 | -5.9 | dairy | / |
| MM135 | Magareći Mlin | N/A | body | / | / | / | / | / | / |
| MM136 | Magareći Mlin | N/A | body | / | / | / | / | / | / |
| MM137 | Magareći Mlin | 430 | rim | / | / | / | / | / | / |
| MM138 | Magareći Mlin | 210 | rim/body | / | / | / | / | / | / |
| MM139 | Magareći Mlin | 420 | rim/body | / | / | / | / | / | / |
| MM140 | Magareći Mlin | 310 | rim/body | 46 | -27.5 | -32.3 | -4.8 | dairy | / |
| MM141 | Magareći Mlin | 220 | rim/body | / | / | / | / | / | / |
| MM142 | Magareći Mlin | 210 | rim/body | 107 | -26.1 | -31.3 | -5.2 | dairy* | beeswax |
| MM143 | Magareći Mlin | 210 | rim/body | / | / | / | / | / | / |
| MM144 | Magareći Mlin | 220 | rim | / | / | / | / | / | / |
| MM145 | Magareći Mlin | 420 | rim/body | 31 | -28 | -30.7 | -2.7 | ruminant adipose | / |
| MM146 | Magareći Mlin | 310 | rim/body | / | / | / | / | / | / |
| MM147 | Magareći Mlin | 430 | rim/body | / | / | / | / | / | / |
| MM148 | Magareći Mlin | 220 | rim/body | / | / | / | / | / | / |
| MM149 | Magareći Mlin | 310 | rim/body | 112 | / | / | / | / | beeswax |
| MM150 | Magareći Mlin | 330 | rim/body | / | / | / | / | / | / |
| MM151 | Magareći Mlin | 420 | rim/body | / | / | / | / | / | / |
| MM152 | Magareći Mlin | N/A | body | / | / | / | / | / | / |
| MM153 | Magareći Mlin | 310 | rim | / | / | / | / | / | / |
| MM154 | Magareći Mlin | 210 | rim | / | / | / | / | / | / |
| MM155 | Magareći Mlin | 310 | rim | / | / | / | / | / | / |
| MM156 | Magareći Mlin | N/A | rim/body | / | / | / | / | / | / |
| MM157 | Magareći Mlin | 320 | rim | / | / | / | / | / | / |
| MM158 | Magareći Mlin | 420 | rim/body | / | / | / | / | / | / |
| MM159 | Magareći Mlin | 210 | rim/body | 26 | / | / | / | / | / |
| MM160 | Magareći Mlin | 220 | rim/body | 51 | -26.9 | -32.4 | -5.4 | dairy | / |
| MM161 | Magareći Mlin | 210 | rim/body | / | / | / | / | / | / |
| MM162 | Magareći Mlin | 420 | rim/body | / | / | / | / | / | / |
| MM163 | Magareći Mlin | 440 | rim/body | / | / | / | / | / | / |
| MM164 | Magareći Mlin | 210 | rim | / | / | / | / | / | / |
| MM165 | Magareći Mlin | 430 | rim/body | 53 | -25.8 | -28.1 | -2.3 | ruminant adipose | / |
| MM166 | Magareći Mlin | 430 | rim/body | / | / | / | / | / | / |
| MM167 | Magareći Mlin | 210 | rim/body | / | / | / | / | / | / |
| MM168 | Magareći Mlin | 210 | rim/body | / | / | / | / | / | / |
| MM169 | Magareći Mlin | N/A | body | / | / | / | / | / | / |
| MM170 | Magareći Mlin | 210 | rim/body | / | / | / | / | / | / |
| MM171 | Magareći Mlin | 210 | rim/body | / | / | / | / | / | / |
| MM172 | Magareći Mlin | 310 | rim | / | / | / | / | / | / |
| MM173 | Magareći Mlin | 330 | rim/body | / | / | / | / | / | / |
| MM174 | Magareći Mlin | N/A | base/body | / | / | / | / | / | / |
| MM175 | Magareći Mlin | 420 | rim/body | / | / | / | / | / | / |
| MM176 | Magareći Mlin | 310 | rim | / | / | / | / | / | / |
| MM177 | Magareći Mlin | 420 | rim/body | / | / | / | / | / | / |
| MM178 | Magareći Mlin | 210 | rim/body | / | / | / | / | / | / |
| MM179 | Magareći Mlin | 310 | rim/body | 56 | -28.1 | -30.7 | -2.6 | ruminant adipose | / |
| MM180 | Magareći Mlin | 330 | rim/body | / | / | / | / | / | / |
| MM181 | Magareći Mlin | 310 | rim/body | / | / | / | / | / | / |
| MM182 | Magareći Mlin | 320 | rim/body | 42 | -25.4 | -29.9 | -4.5 | dairy | / |
| MM183 | Magareći Mlin | 310 | rim/body | / | / | / | / | / | / |
| MM184 | Magareći Mlin | 420 | rim/neck | / | / | / | / | / | / |
| MM185 | Magareći Mlin | 210 | rim | 19 | -24.7 | -25 | -0.3 | ruminant adipose/ Mixture ruminant/non-ruminant | / |
| MM186 | Magareći Mlin | 420 | rim | / | / | / | / | / | / |
| MM187 | Magareći Mlin | 420 | rim/body | / | / | / | / | / | / |
| MM188 | Magareći Mlin | 430 | rim/neck | 84 | -27.3 | -33.7 | -6.5 | dairy | / |
| MM189 | Magareći Mlin | 310 | rim | / | / | / | / | / | / |
| MM190 | Magareći Mlin | 310 | rim | / | / | / | / | / | / |
| MM191 | Magareći Mlin | 430 | rim/neck | / | / | / | / | / | / |
| MM192 | Magareći Mlin | 430 | rim/body | / | / | / | / | / | / |
| MM193 | Magareći Mlin | 210 | rim | / | / | / | / | / | / |
| MM194 | Magareći Mlin | 430 | rim/body | / | / | / | / | / | / |
| MM195 | Magareći Mlin | 420 | rim/body | / | / | / | / | / | / |
| MM196 | Magareći Mlin | 330 | rim/body | / | / | / | / | / | / |
| MM197 | Magareći Mlin | N/A | body | / | / | / | / | / | / |
| MM198 | Magareći Mlin | 230 | rim/body | 157 | -28.7 | -30.8 | -2.1 | ruminant adipose | / |
| MM199 | Magareći Mlin | 210 | rim/body | 21 | -27.7 | -32.7 | -4.9 | dairy | / |
| RG200 | Rutonjina Greda | 210 | rim/body | / | / | / | / | / | / |
| RG201 | Rutonjina Greda | N/A | base/body | / | / | / | / | / | / |
| RG202 | Rutonjina Greda | 210 | rim/body | / | / | / | / | / | / |
| RG203 | Rutonjina Greda | 220 | rim/body | / | / | / | / | / | / |
| RG204 | Rutonjina Greda | 210 | rim/body | / | / | / | / | / | / |
| RG205 | Rutonjina Greda | 320 | rim/body | / | / | / | / | / | / |
| RG206 | Rutonjina Greda | 410 | rim/body | / | / | / | / | / | / |
| RG207 | Rutonjina Greda | 210 | rim/body | 22 | -26.9 | -30.3 | -3.5 | dairy | / |
| RG208 | Rutonjina Greda | 210 | rim | / | / | / | / | / | / |
| RG209 | Rutonjina Greda | 420 | rim | / | / | / | / | / | / |
| RG210 | Rutonjina Greda | 210 | rim | / | / | / | / | / | / |
| RG211 | Rutonjina Greda | 220 | rim/body | / | / | / | / | / | / |
| RG212 | Rutonjina Greda | 420 | rim/body | / | / | / | / | / | / |
| RG213 | Rutonjina Greda | 440 | rim/body | / | / | / | / | / | / |
| RG214 | Rutonjina Greda | 220 | rim/body | / | / | / | / | / | / |
| RG215 | Rutonjina Greda | 210 | rim/body | 138 | -26.8 | -31.4 | -4.6 | dairy | / |
| RG216 | Rutonjina Greda | 310 | rim | 28 | -29.9 | -32.6 | -2.7 | ruminant adipose | / |

* not included in Figure 3
