# Supplementary material for: Living off the land: Terrestrial-based diet and dairying in the farming communities of the Neolithic Balkans
Source: PLoS One. 2020 Aug 20;15(8):e0237608. doi: 10.1371/journal.pone.0237608 (PMC7444498; doi:10.1371/journal.pone.0237608)
Supplement: S2 File — (DOCX) [file pone.0237608.s002.docx]

The importance of stock herding and dairying in the Neolithic Balkans and the subsistence diversity

Supplement Information 2: Graphic representation of pottery samples

Darko Stojanovski, Ivana Živaljević, Vesna Dimitrijević, Julie Dunne, Richard P. Evershed, Marie Balasse, Adam Dowle, Jessica Hendy, Krista McGrath, Roman Fischer, Camilla Speller, Jelena Jovanović, Emmanuelle Casanova, Timothy Knowles, Lidija Balj, Goce Naumov, Anđelka Putica, Andrej Starović, Sofija Stefanović

Morpho-typology:

T210 = hemispherical dishes

T220 = conical dishes

T230 = dishes with everting rim

T310 = spherical bowls

T320 = necked bowls

T330 = bowls with everting rim

T410 = spherical jars (hole-mouth)

T420 = spherical jars with slightly protruding neck (pear-shaped)

T430 = necked jars

T440 = jars with everting rim (S-shaped)

T500 = altars

Assignment of detected lipids:

Yellow = dairy fats

Red = ruminant adipose fats

Pink = ruminant / non-ruminant adipose fat mixture

Blue = fats from aquatic resources (fish)

Green = plant fats and oils

Brown = beeswax / honey

The sample codes under graphics correspond with the codes in the tabular representation in Supplement 1.

Vrbjanska Čuka, Pelagonia, Macedonia, South-central Balkan

6000-5700 calBC


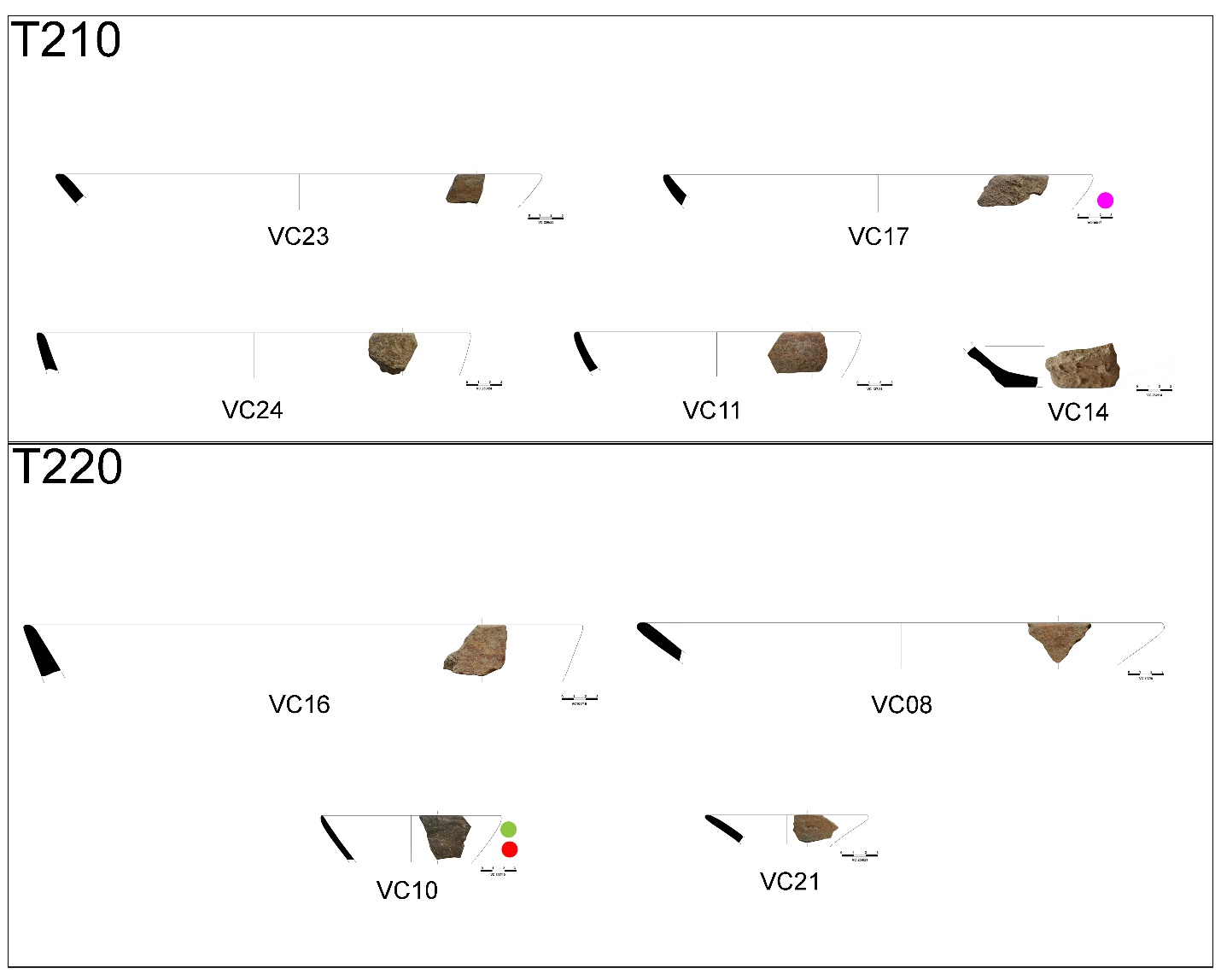


Fig. S2.1 Dish samples from Vrbjanska Čuka


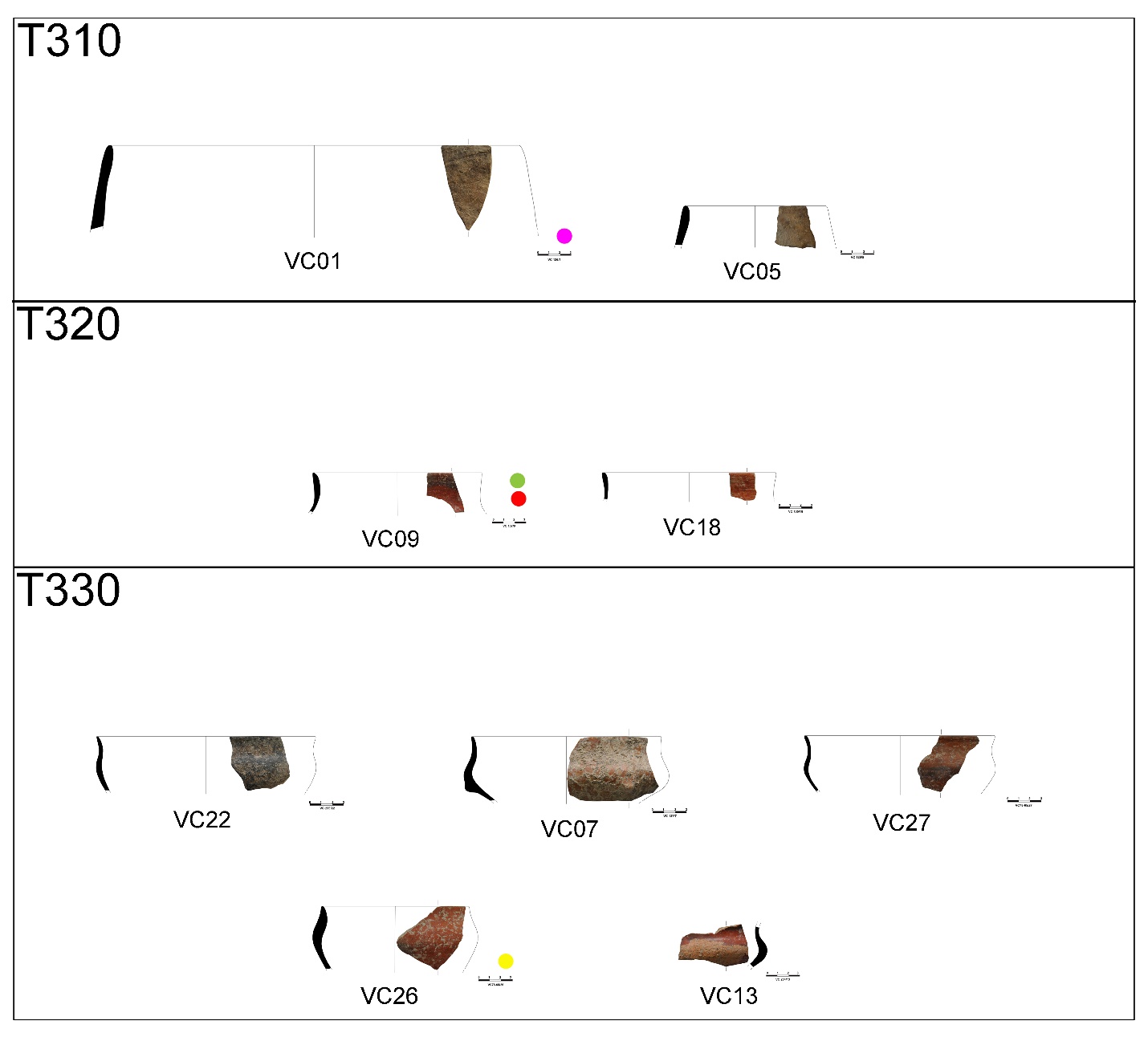


Fig. S2.2 Bowl samples from Vrbjanska Čuka


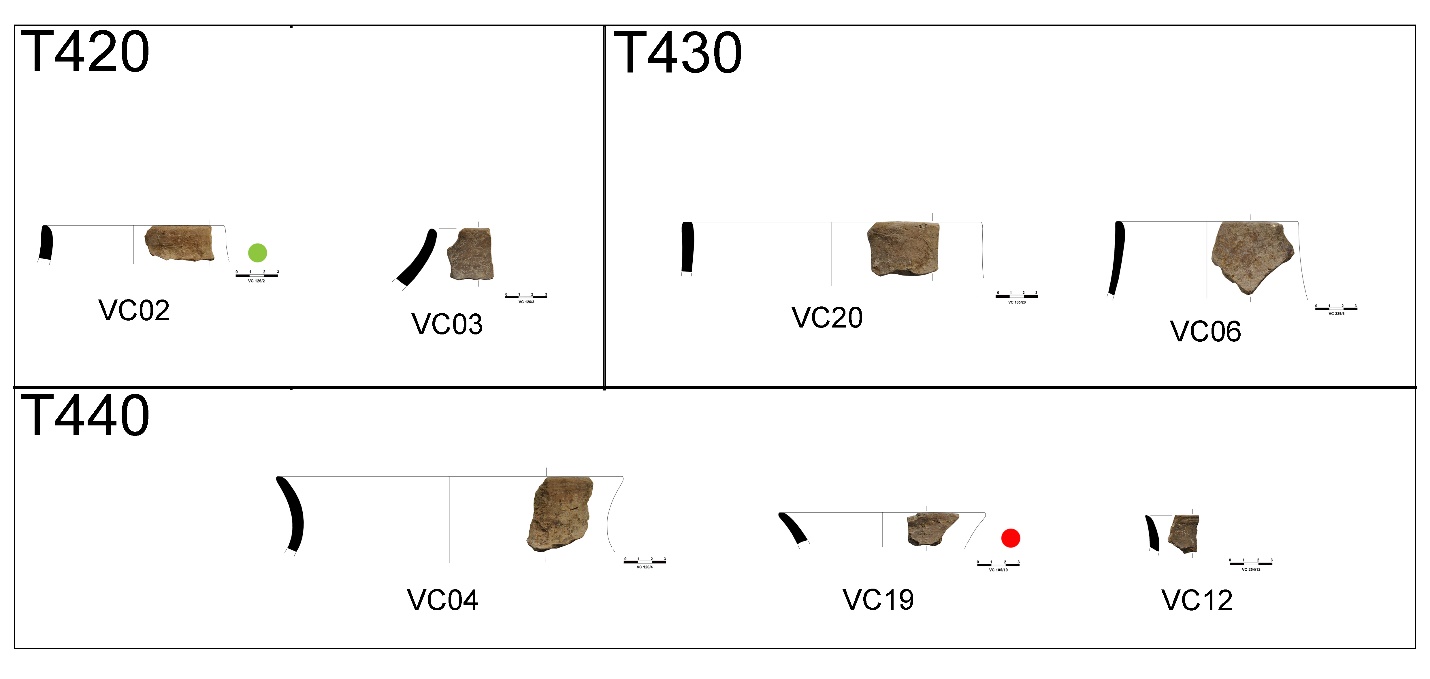


Fig. S2.3 Jar samples from Vrbjanska Čuka


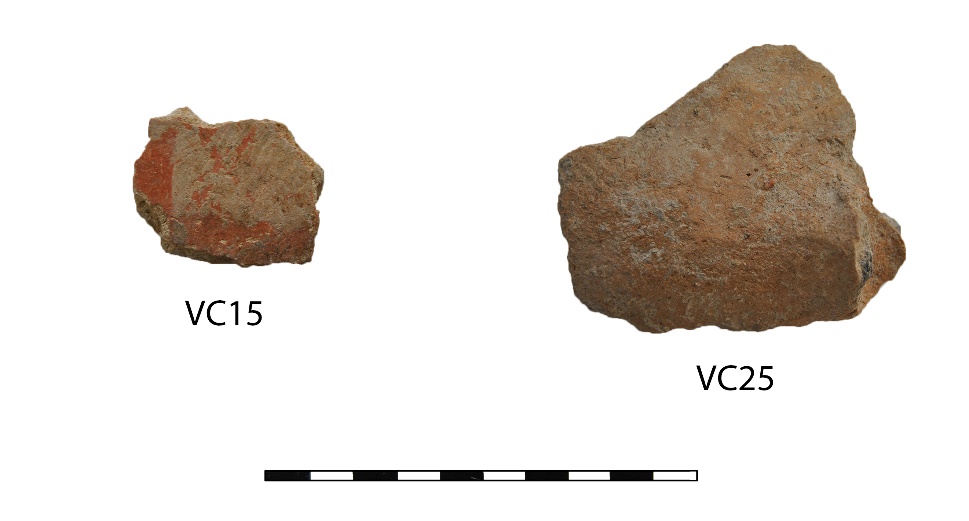


Fig. S2.4 Samples from Vrbjanska Čuka from vessels with uncertain morphology

Starčevo-Grad, South-eastern Vojvodina, Serbia, northern Balkan

first half of the 6^th^ millennium calBC


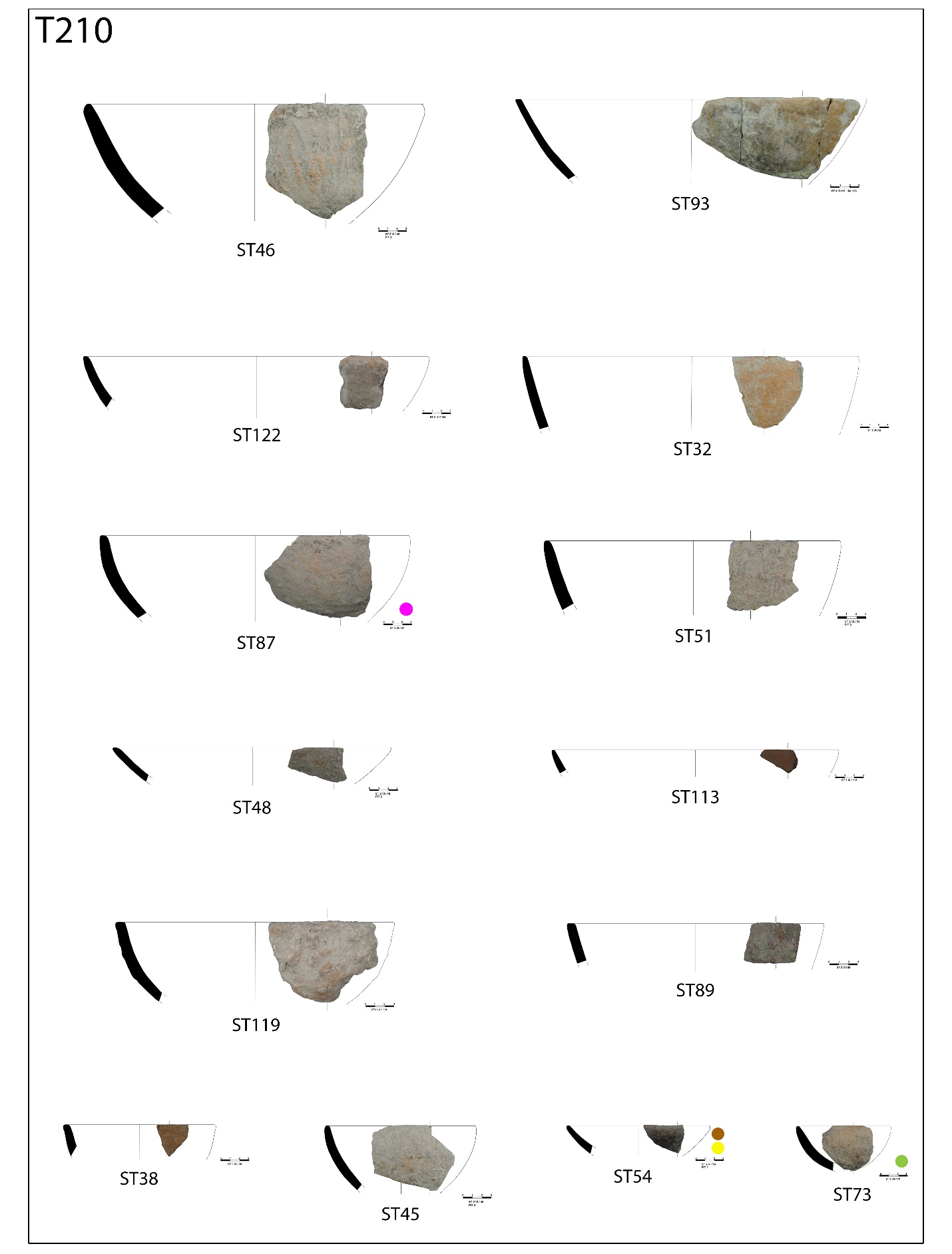


Fig. S2.5 Spherical shaped dish samples from Starčevo-Grad


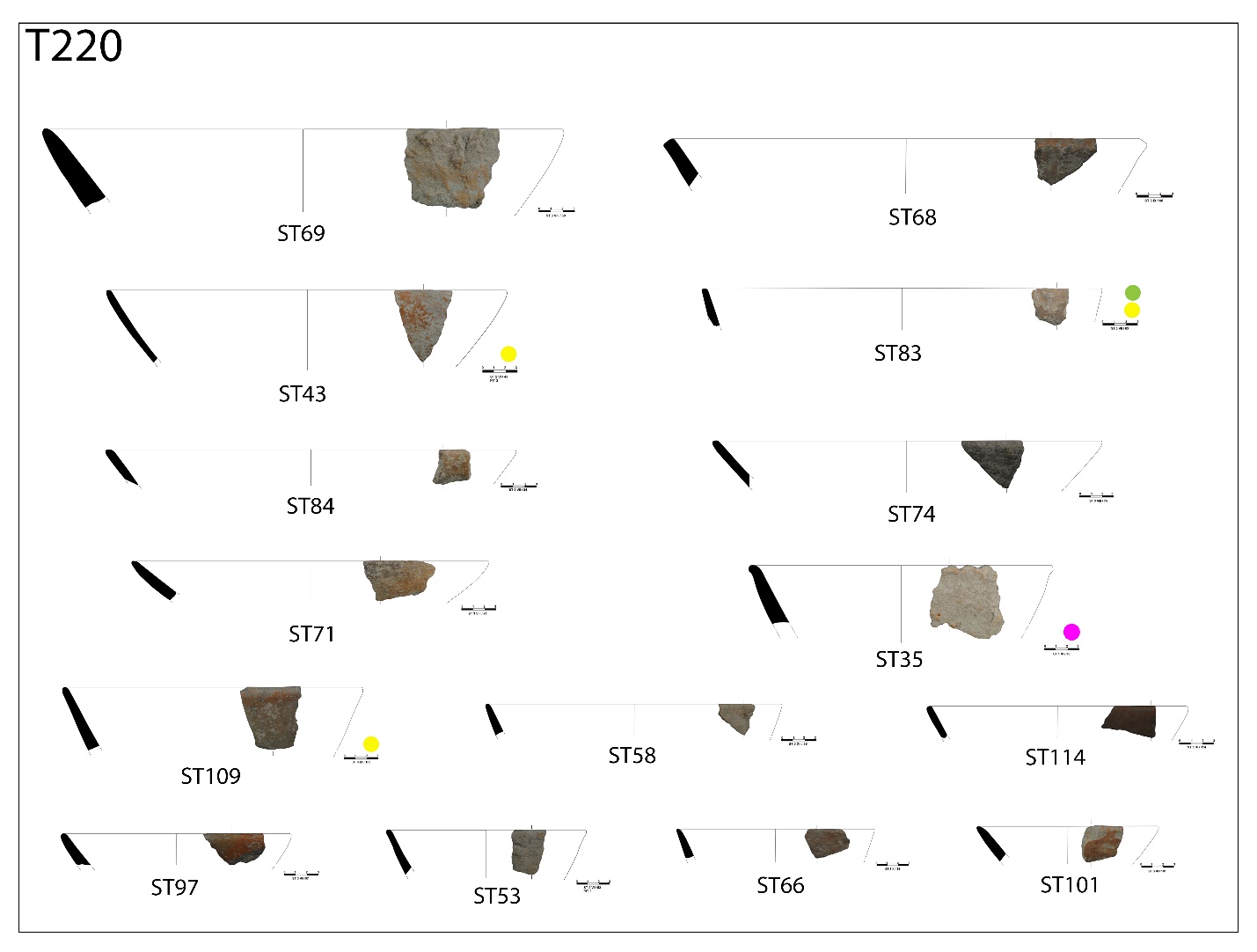


Fig. S2.6 Conical shaped dish samples from Starčevo-Grad


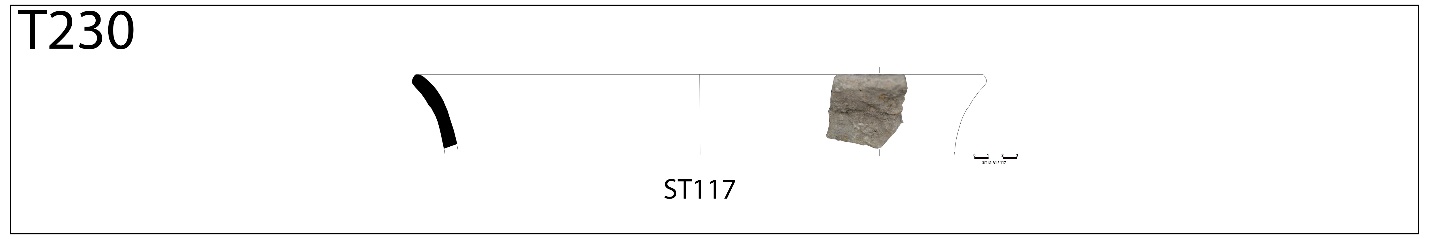


Fig. S2.7 Sample from dish with everted rim from Starčevo-Grad


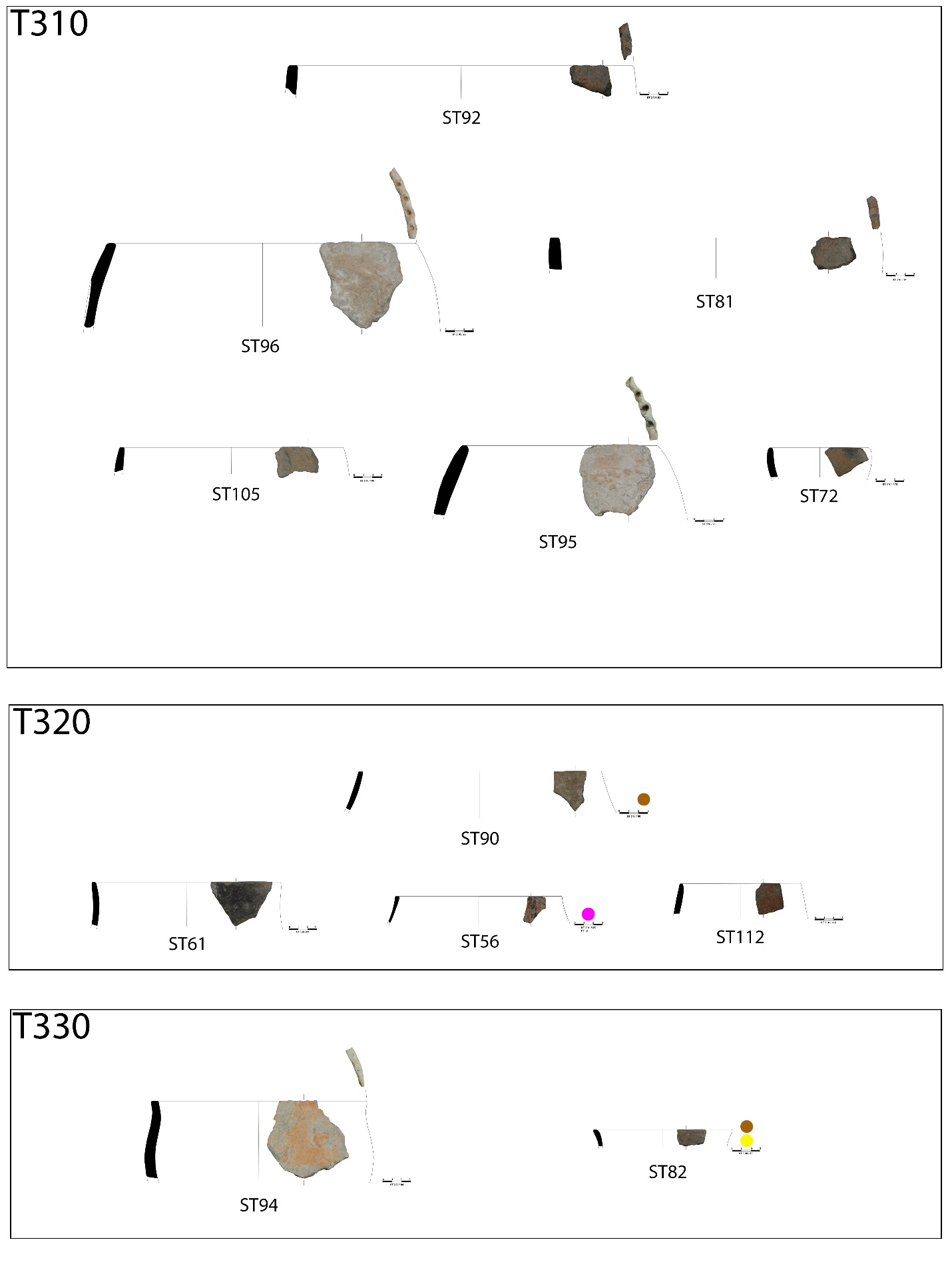


Fig. S2.8 Bowl samples from Starčevo-Grad


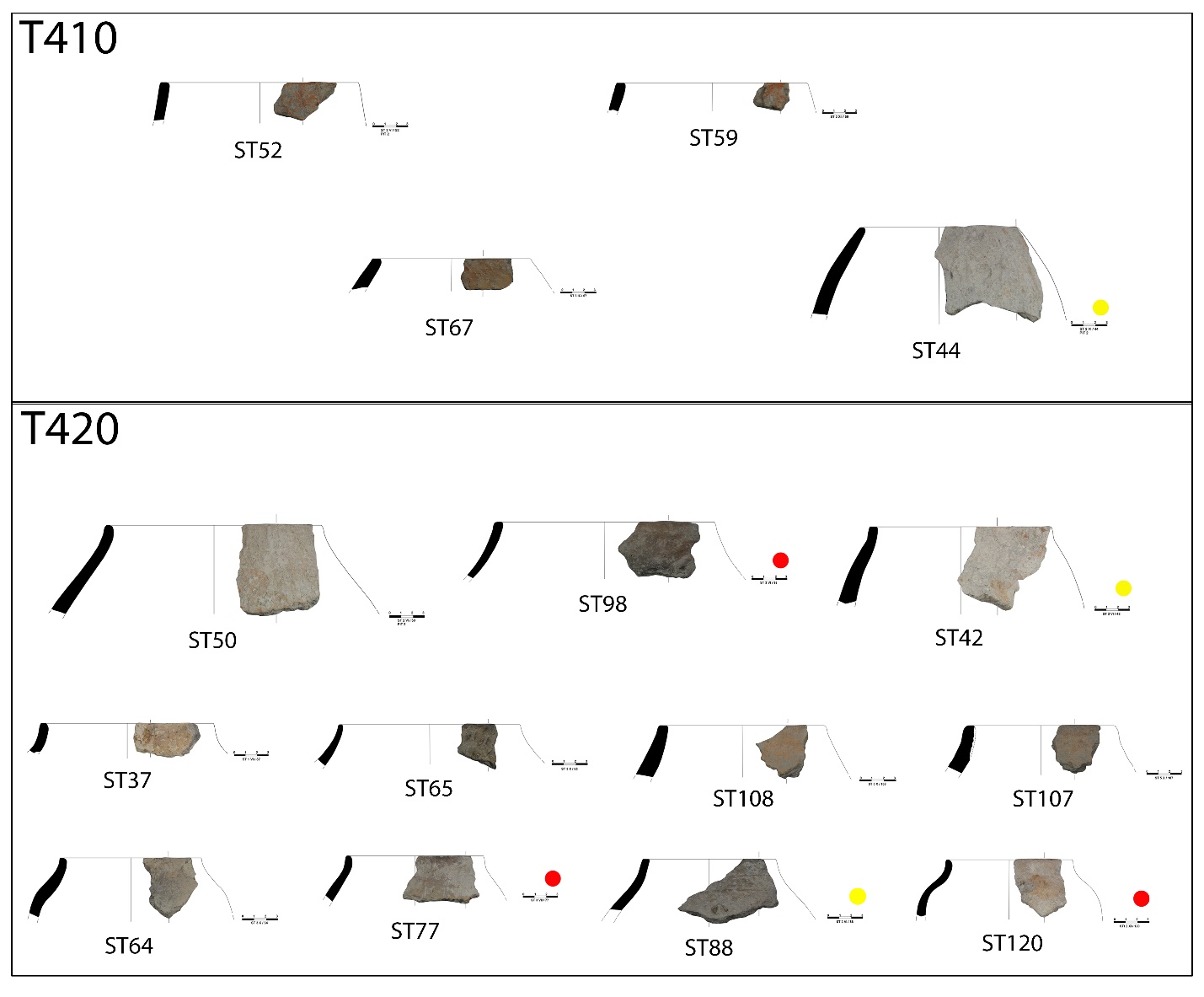


Fig. S2.9 Spherical and pear-shaped jar samples from Starčevo-Grad


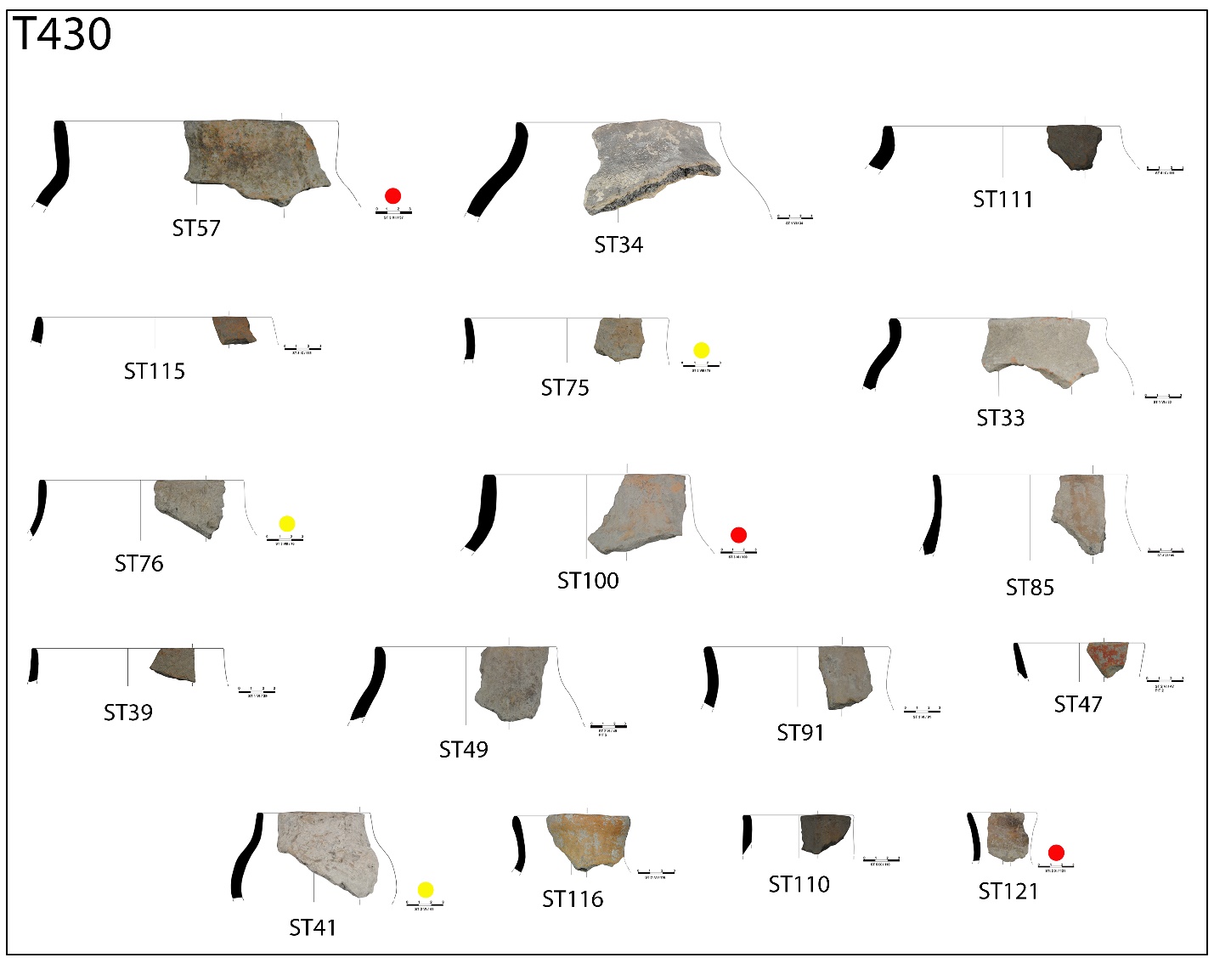


Fig. S2.10 Necked jar samples from Starčevo-Grad


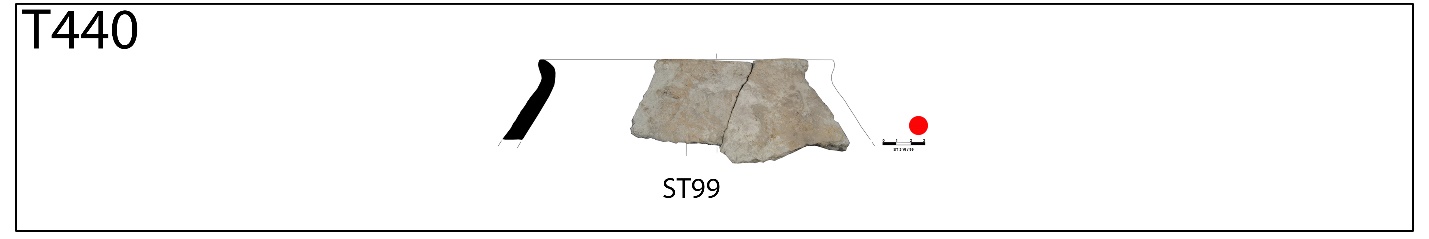


Fig. S2.11 Sample from jar with everting rim (S-shaped) from Starčevo-Grad


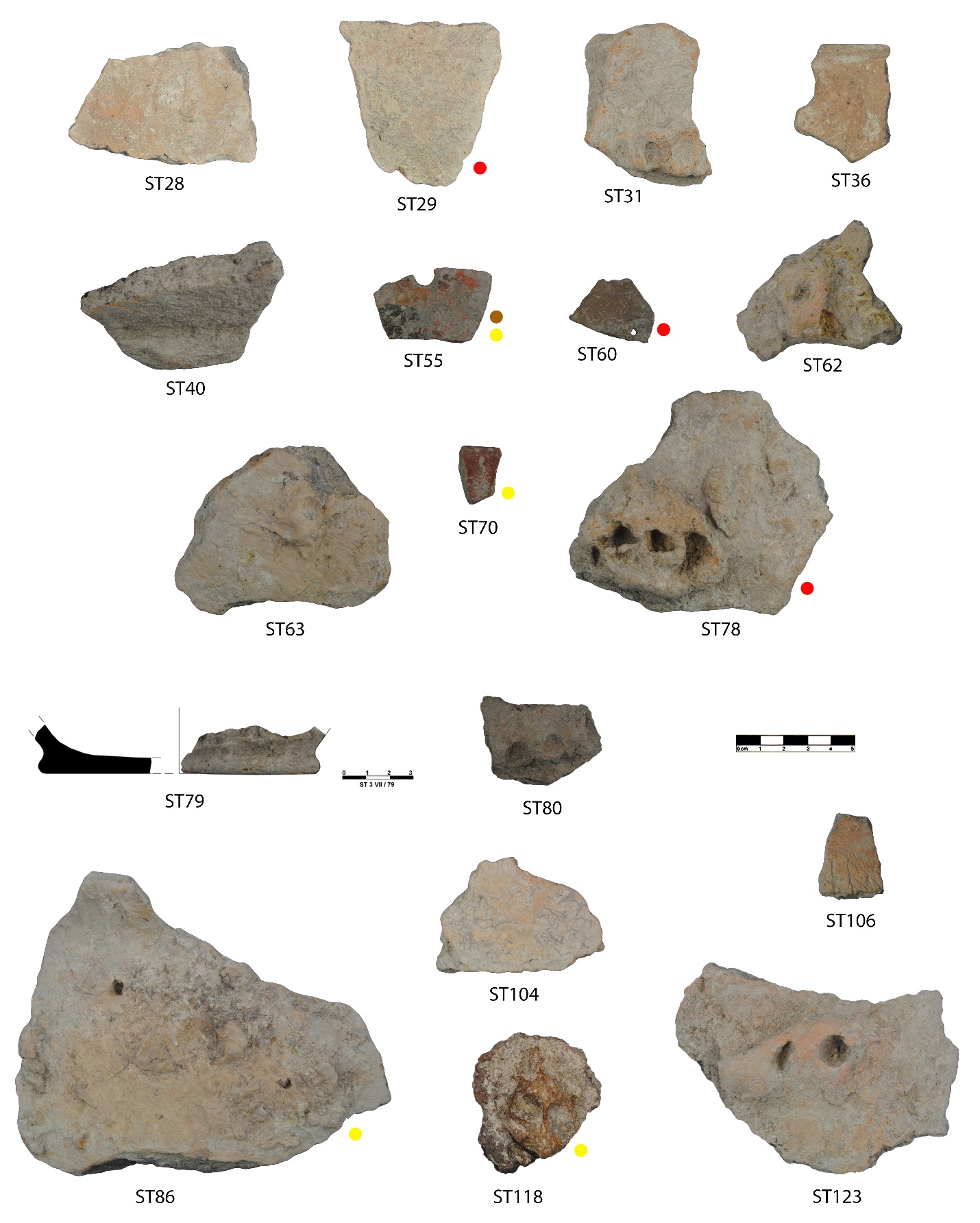


Fig. S2.12 Samples from Starčevo-Grad from vessels with uncertain morphology


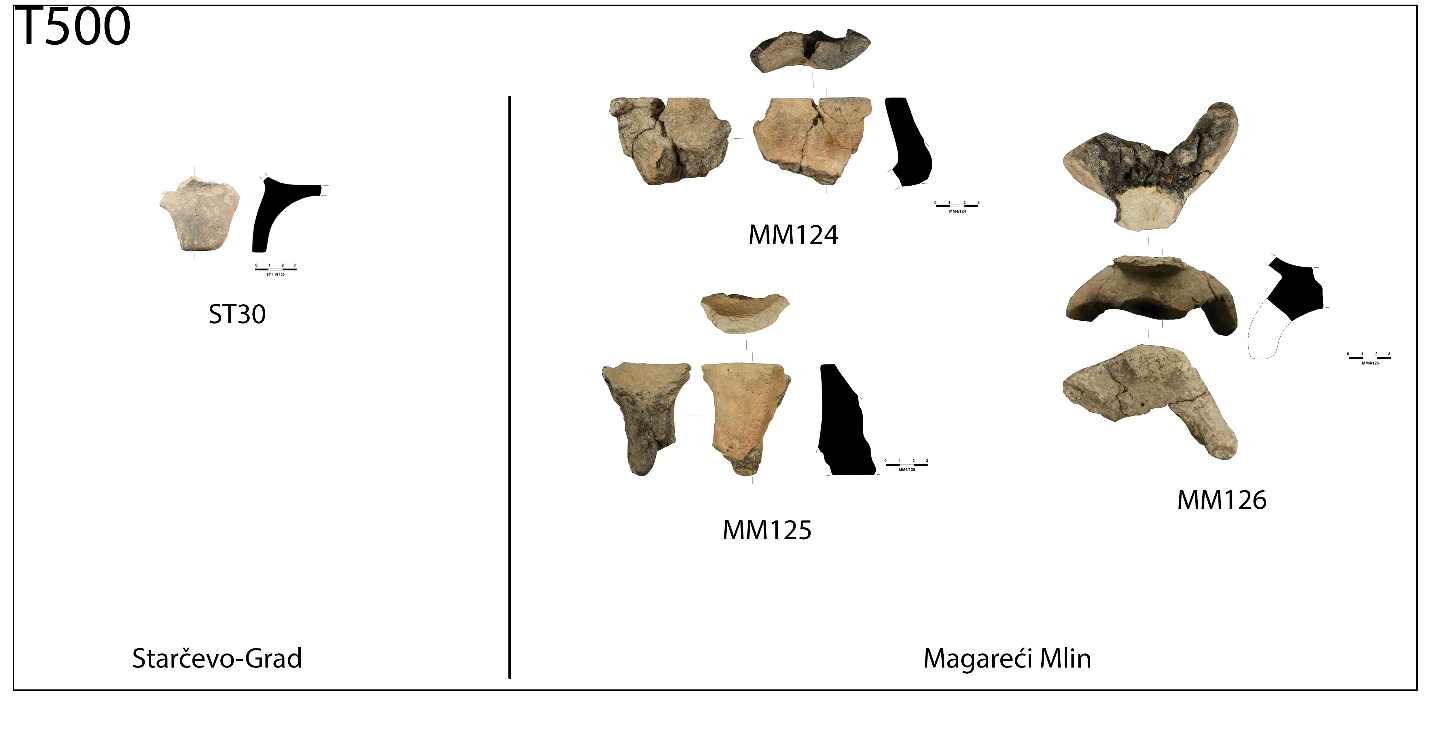


Fig. S2.13 Altar samples (legs and recipients) from Starčevo-Grad and Magareći Mlin

Magareći Mlin, western Vojvodina, Serbia, northern Balkan

Early 6^th^ millennium calBC


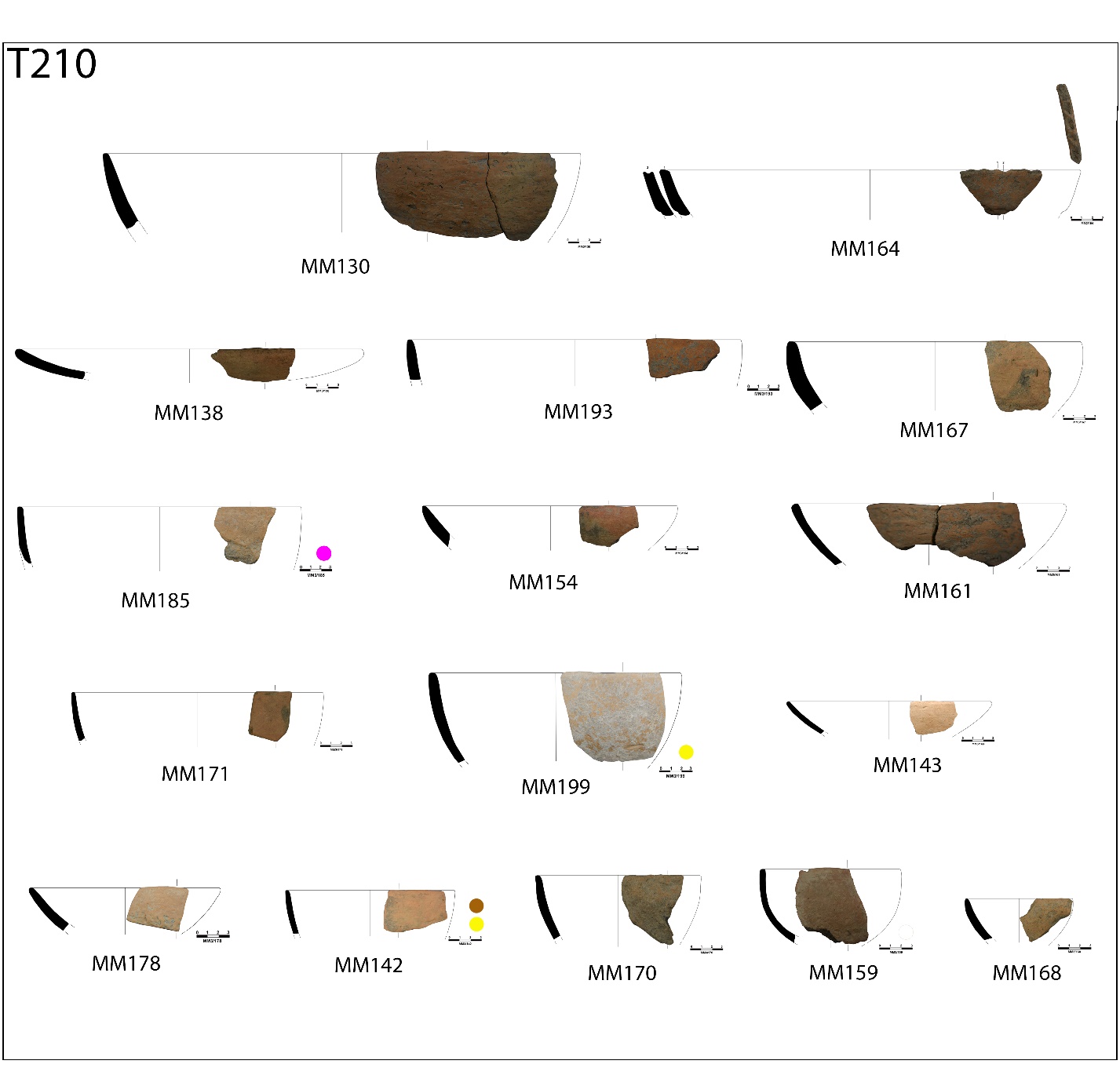


Fig. S2.14 Spherical dish samples from Magareći Mlin


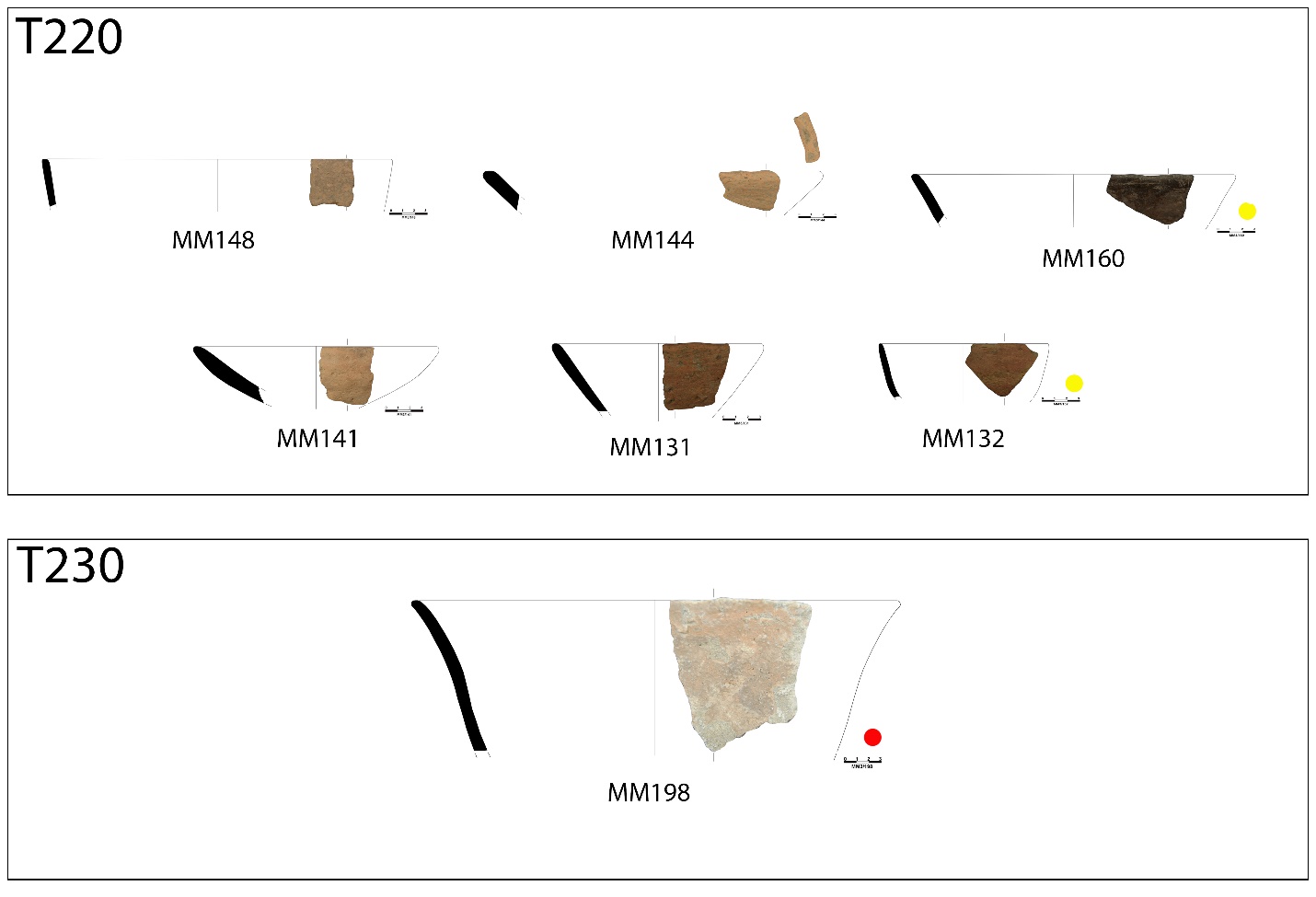


Fig. S2.15 Conical and everted-rim dish samples from Magareći Mlin


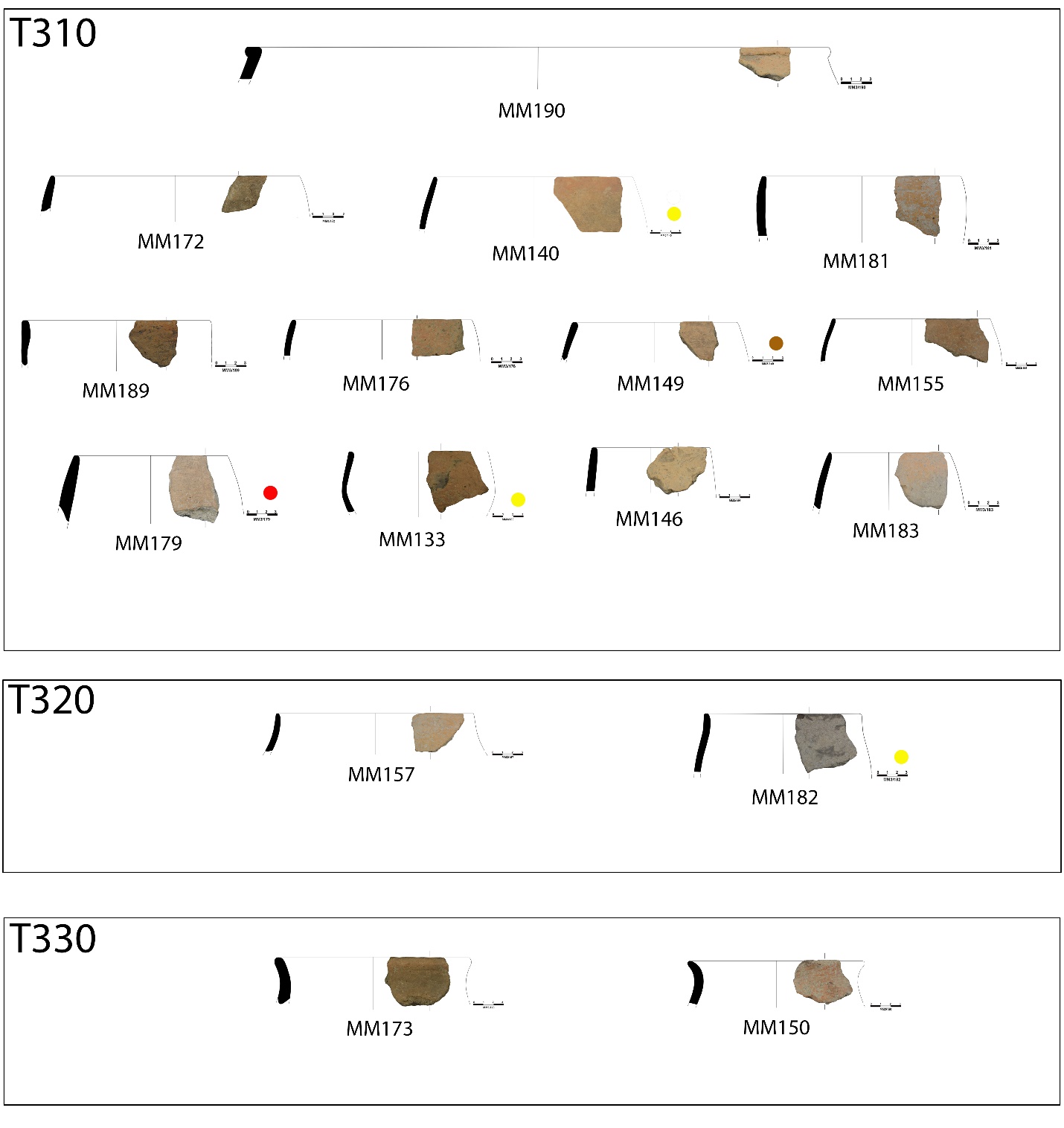


Fig. S2.16 Bowl samples from Magareći Mlin


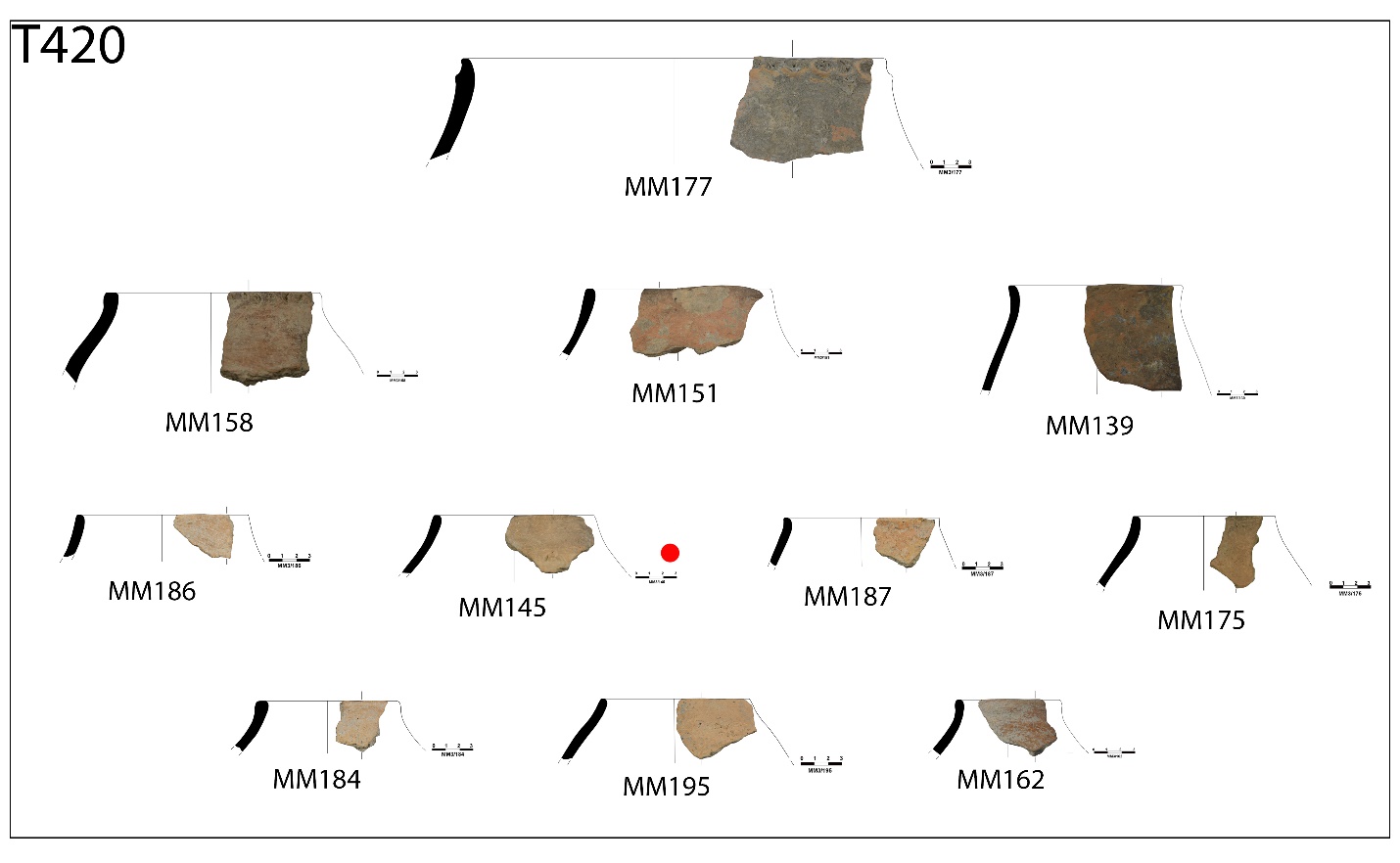


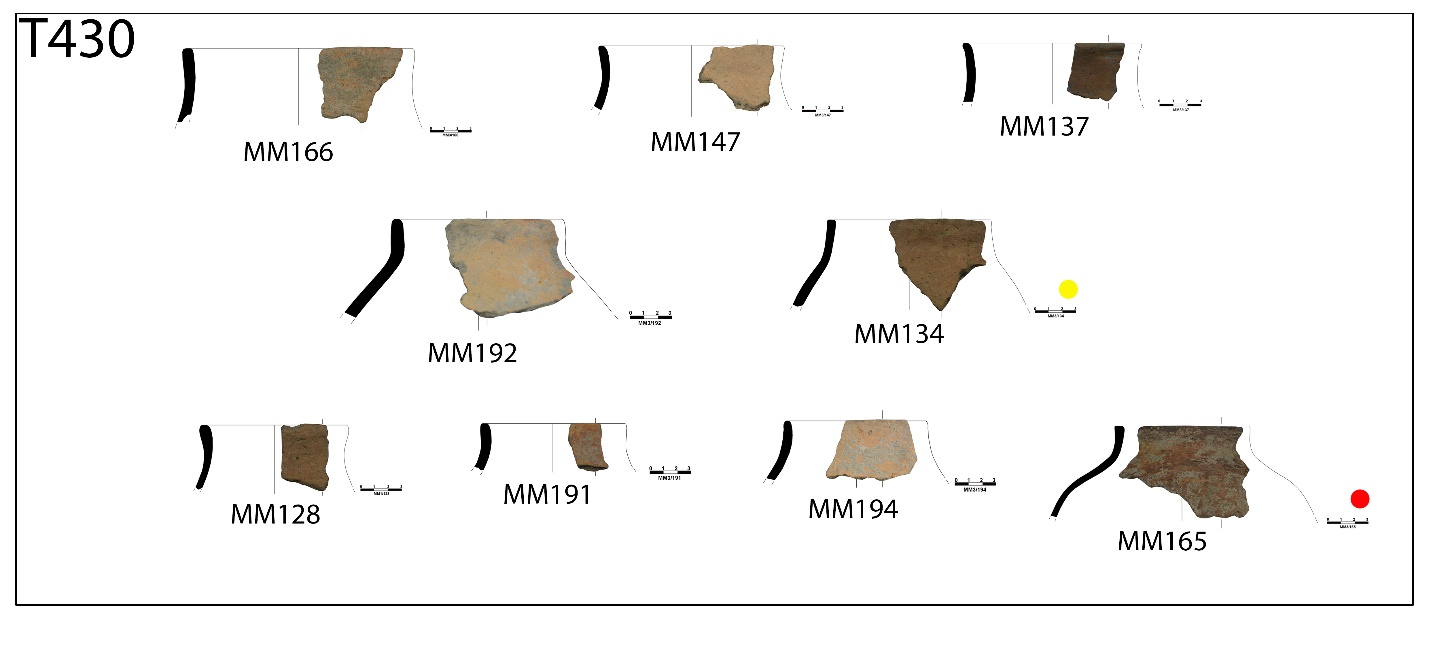


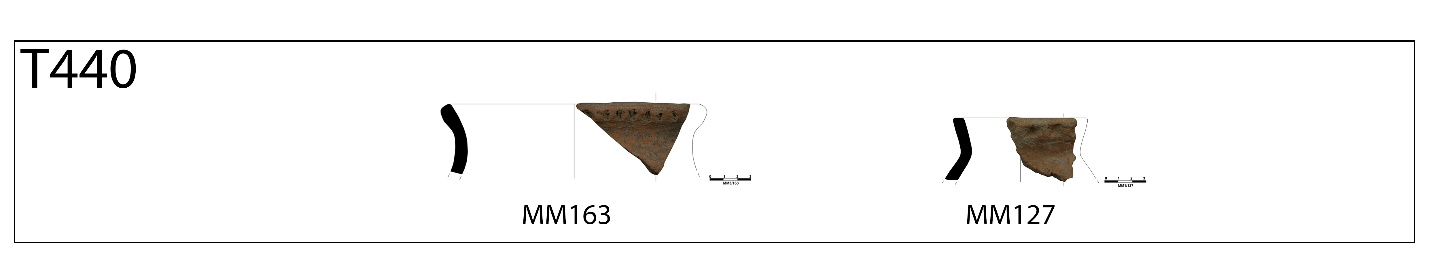


Fig. S2.17 Jar samples from Magareći Mlin


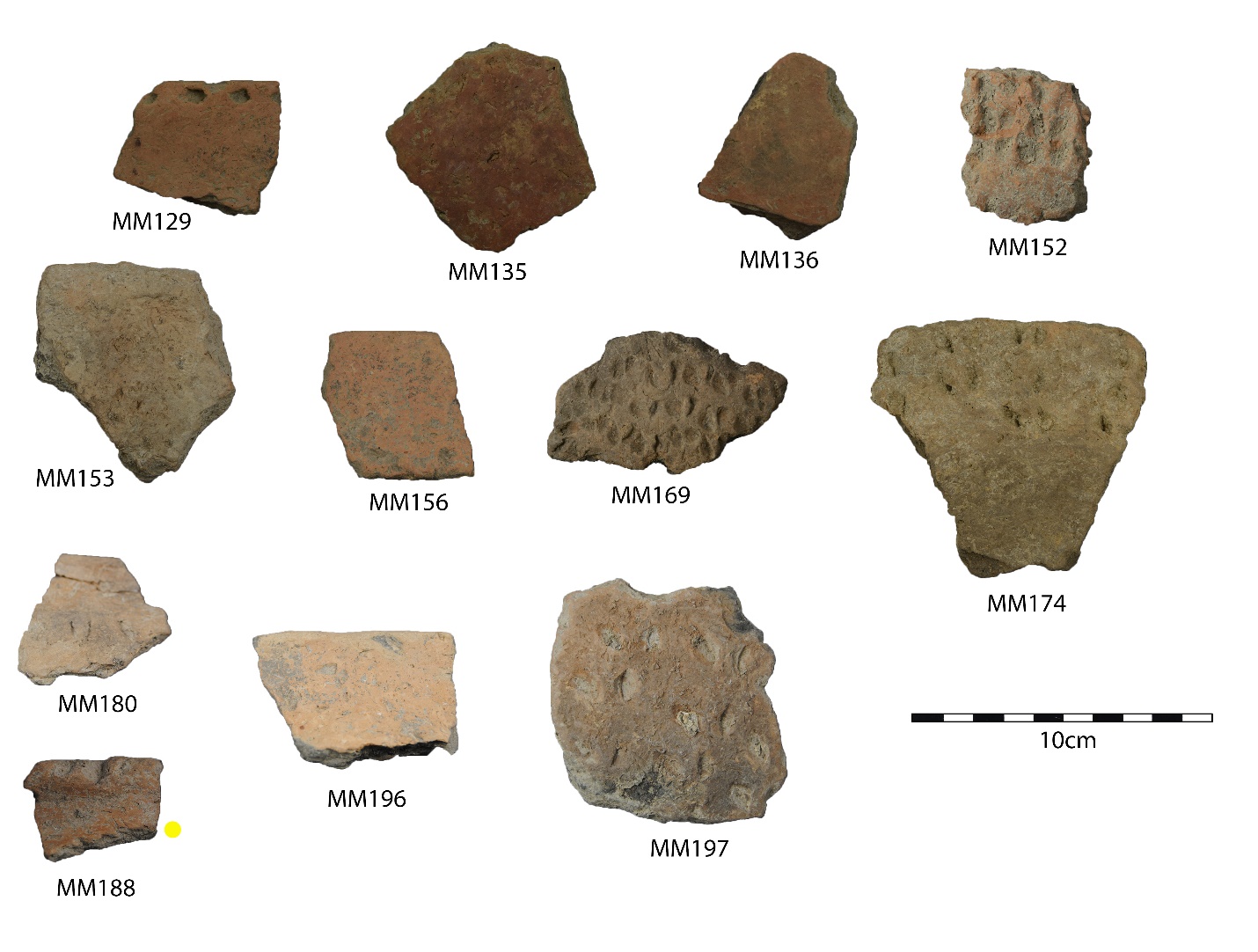


Fig. S2.18 Samples from Magareći Mlin from vessels with uncertain morphology

Rutonjina Greda, central Vojvodina, Serbia, northern Balkan

first half of the 6^th^ millennium calBC


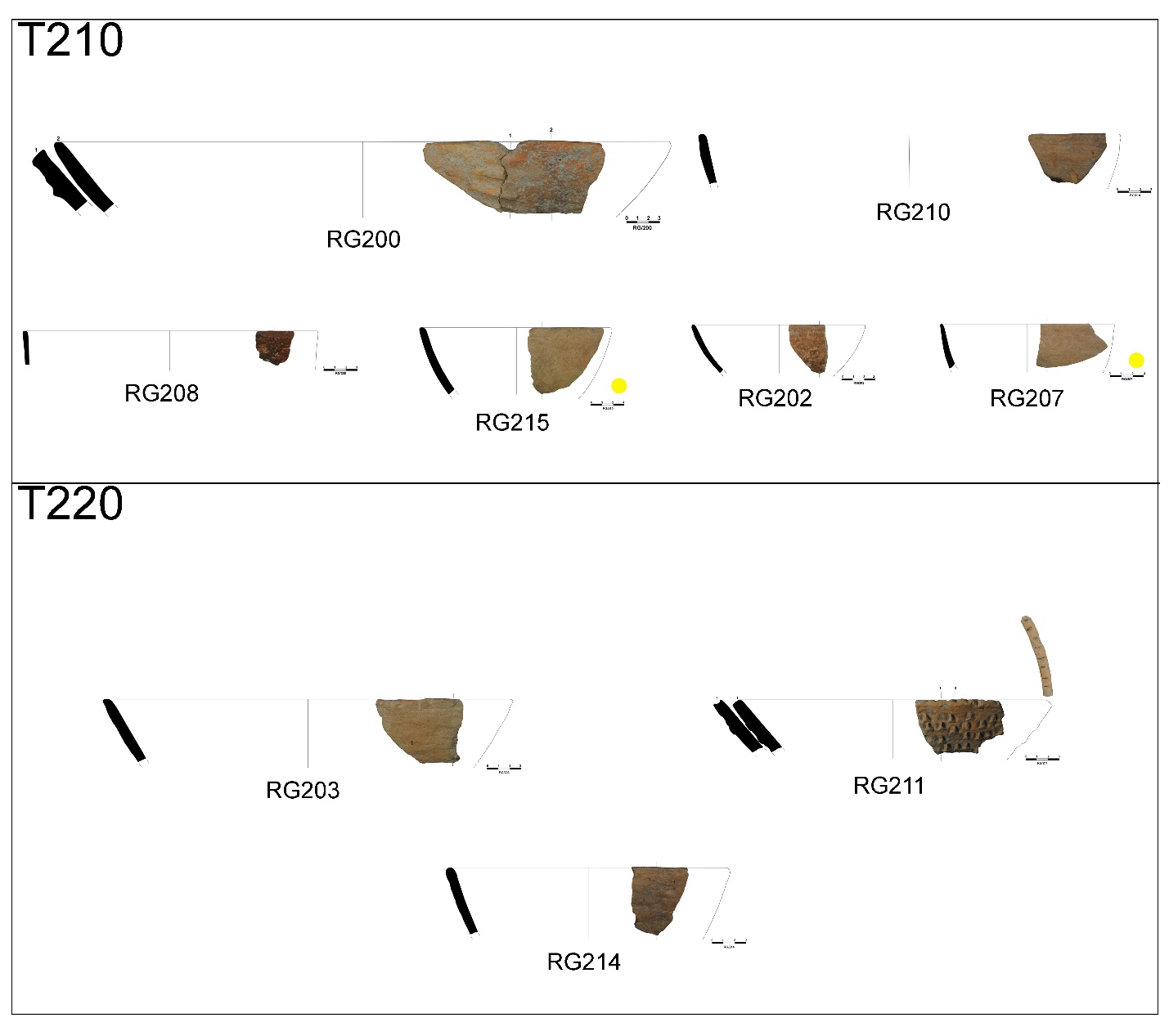


Fig. S2.19 Dish samples from Rutonjina Greda


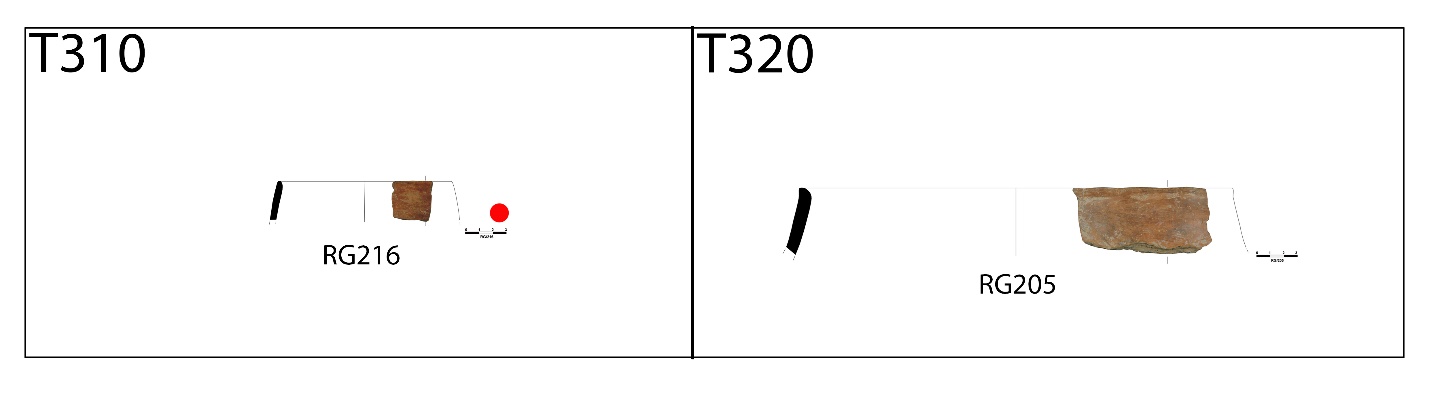


Fig. S2.20 Bowl samples from Rutonjina Greda


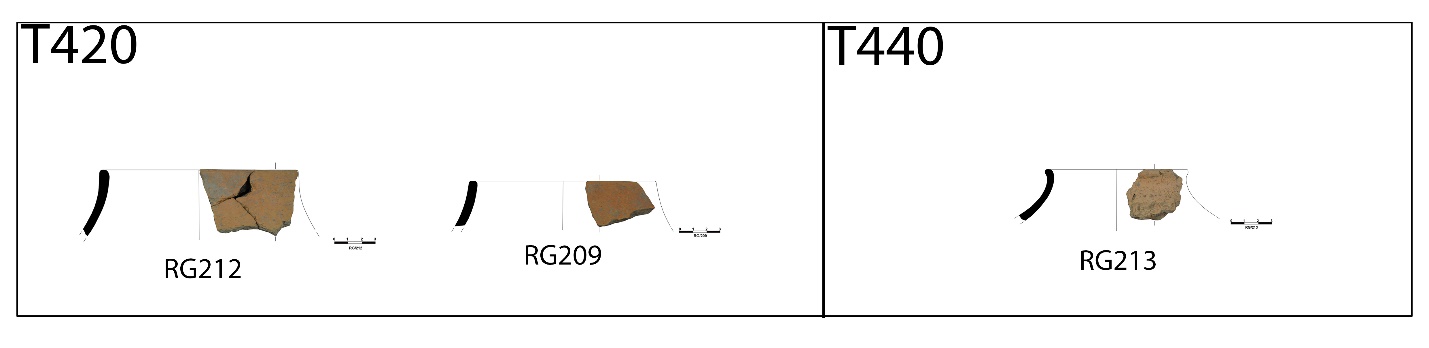


Fig. S2.21 Jar samples from Rutonjina Greda


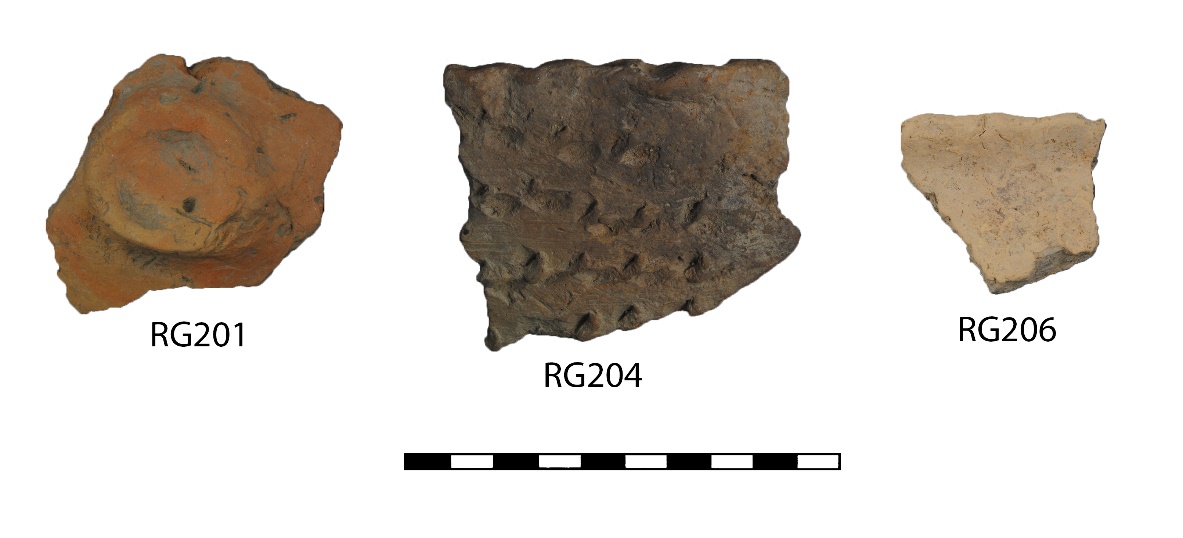


Fig. S2.22 Samples from Rutonjina Greda from vessels with uncertain morphology
