# Supplementary material for: Living off the land: Terrestrial-based diet and dairying in the farming communities of the Neolithic Balkans
Source: PLoS One. 2020 Aug 20;15(8):e0237608. doi: 10.1371/journal.pone.0237608 (PMC7444498; doi:10.1371/journal.pone.0237608)
Supplement: S3 File — (DOCX) [file pone.0237608.s003.docx]

The importance of stock herding and dairying in the Neolithic Balkans and the subsistence diversity

Supplementary Information 3: Dentine collagen results

Darko Stojanovski, Ivana Živaljević, Vesna Dimitrijević, Julie Dunne, Richard P. Evershed, Marie Balasse, Adam Dowle, Jessica Hendy, Krista McGrath, Roman Fischer, Camilla Speller, Jelena Jovanović, Emmanuelle Casanova, Timothy Knowles, Lidija Balj, Goce Naumov, Anđelka Putica, Andrej Starović, Sofija Stefanović

Quality of collagen extracts:

Following recommendations by DeNiro [80], Ambrose [81] and Van Klinken [82] collagen extracts should contain at least 30% carbon and 11% nitrogen. Among a total number of 75 samples, 63 meet this criterion (%N = 11.3 % - 15.9 % and %C = 30.5 % - 43.3 % Table). In the 12 remaining samples, N content varies from 7.7 % to 10.7 % and C content varies from 21.2 % to 29.5 %; these samples, which occur in isolation within sequences, yielded δ^15^N and δ^13^C values consistent with the remaining values in their respective sequences (Figure). All 75 collagen samples yielded C:N ratios of 3.1 or 3.2. The sampling performed on the anterior and posterior lobes of each molar shows great consistency in δ^15^N and δ^13^C values (Figure), suggesting a good preservation of the original stable isotope ratios.

Table S3.1: Carbon (%C) and nitrogen content (%N), atomic C and N ratio (C:N) and stable carbon (δ^13^C) and nitrogen (δ^15^N) isotope values in the collagen extracts from tooth dentine. Samples highlighted in red have a N content lower than 11% and a C content lower than 30%

| **LBMM002 M1** | **sample** | **mid-point (mm)** | **%C** | **%N** | **C/N** | **δ^15^N (‰)** | **δ^13^C (‰)** |
| --- | --- | --- | --- | --- | --- | --- | --- |
| anterior lobe | 1 | 43.3 | 36.9 | 13.6 | 3.2 | 6.57 | -21.42 |
|  | 2 | 38.0 | 37.5 | 13.7 | 3.2 | 6.69 | -21.07 |
|  | 3 | 34.8 | 24.5 | 9.0 | 3.2 | 6.79 | -21.06 |
|  | 4 | 32.7 | 40.2 | 14.6 | 3.2 | 7.00 | -20.96 |
|  | 5 | 29.5 | 29.0 | 10.6 | 3.2 | 7.22 | -20.91 |
|  | 6 | 25.9 | 30.5 | 11.3 | 3.2 | 7.11 | -20.83 |
|  | 7 | 22.6 | 28.5 | 10.6 | 3.1 | 7.56 | -20.99 |
|  | 8 | 18.7 | 35.4 | 13.2 | 3.1 | 7.84 | -20.92 |
|  | 9 | 14.4 | 36.5 | 13.6 | 3.1 | 8.39 | -21.14 |
|  | 10 | 10.1 | 33.2 | 12.2 | 3.2 | 8.57 | -21.32 |
|  | 11 | 6.0 | 34.8 | 12.9 | 3.1 | 8.47 | -21.67 |
|  | 12 | 2.2 | 36.3 | 13.3 | 3.2 | 8.49 | -21.99 |
| posterior lobe | 1 | 43.6 | 36.2 | 13.1 | 3.2 | 6.47 | -21.42 |
|  | 2 | 40.6 | 37.0 | 13.6 | 3.2 | 6.57 | -21.11 |
|  | 3 | 37.5 | 36.6 | 13.6 | 3.1 | 6.67 | -21.02 |
|  | 4 | 34.0 | 32.7 | 12.0 | 3.2 | 6.75 | -20.89 |
|  | 5 | 30.9 | 31.0 | 11.3 | 3.2 | 6.85 | -20.90 |
|  | 6 | 27.5 | 26.3 | 9.6 | 3.2 | 7.01 | -21.00 |
|  | 7 | 22.4 | 37.9 | 13.7 | 3.2 | 7.44 | -20.93 |
|  | 8 | 18.3 | 25.8 | 9.6 | 3.1 | 7.93 | -21.09 |
|  | 9 | 14.5 | 36.5 | 13.4 | 3.2 | 8.12 | -21.08 |
|  | 10 | 10.8 | 34.0 | 12.6 | 3.1 | 8.61 | -21.28 |
|  | 11 | 6.9 | 33.3 | 12.4 | 3.1 | 8.53 | -21.56 |
|  | 12 | 2.5 | 36.6 | 13.5 | 3.2 | 8.49 | -21.88 |

| **LBMM022 M2** | **sample** | **mid-point (mm)** | **%C** | **%N** | **C/N** | **δ ^15^N (‰)** | **δ ^13^C (‰)** |
| --- | --- | --- | --- | --- | --- | --- | --- |
| anterior lobe | 1 | 58.0 | 40.7 | 15.3 | 3.1 | 5.84 | -20.92 |
|  | 2 | 52.5 | 42.4 | 15.6 | 3.2 | 6.38 | -20.72 |
|  | 3 | 49.6 | 38.9 | 14.2 | 3.2 | 6.55 | -20.80 |
|  | 4 | 46.1 | 40.3 | 14.7 | 3.2 | 6.89 | -20.85 |
|  | 5 | 41.0 | 28.8 | 10.5 | 3.2 | 7.24 | -21.08 |
|  | 6 | 35.7 | 28.5 | 10.4 | 3.2 | 7.57 | -21.23 |
|  | 7 | 30.6 | 35.7 | 13.1 | 3.2 | 8.82 | -21.63 |
|  | 8 | 24.1 | 32.9 | 12.1 | 3.2 | 9.12 | -21.68 |
|  | 9 | 17.6 | 31.7 | 11.6 | 3.2 | 9.24 | -21.57 |
|  | 10 | 12.4 | 35.2 | 12.9 | 3.2 | 9.30 | -21.48 |
|  | 11 | 8.0 | 39.9 | 14.7 | 3.2 | 9.33 | -21.22 |
|  | 12 | 3.2 | 37.1 | 13.7 | 3.1 | 8.95 | -21.04 |
| posterior lobe | 1 | 62.6 | 41.6 | 15.1 | 3.2 | 6.37 | -20.91 |
|  | 2 | 59.1 | 42.7 | 15.5 | 3.2 | 6.41 | -20.82 |
|  | 3 | 54.5 | 43.3 | 15.9 | 3.2 | 6.36 | -20.78 |
|  | 4 | 50.6 | 40.5 | 14.9 | 3.2 | 6.48 | -20.92 |
|  | 5 | 47.6 | 39.1 | 14.3 | 3.2 | 6.48 | -21.00 |
|  | 6 | 44.3 | 38.3 | 14.1 | 3.2 | 6.62 | -20.90 |
|  | 7 | 39.0 | 29.5 | 10.7 | 3.2 | 7.49 | -21.28 |
|  | 8 | 34.2 | 29.1 | 10.6 | 3.2 | 8.04 | -21.48 |
|  | 9 | 29.9 | 39.7 | 14.7 | 3.2 | 8.05 | -21.34 |
|  | 10 | 26.3 | 40.5 | 15.0 | 3.2 | 8.92 | -21.70 |
|  | 11 | 22.5 | 38.7 | 14.3 | 3.2 | 8.93 | -21.69 |
|  | 12 | 17.5 | 37.9 | 14.1 | 3.1 | 9.14 | -21.65 |
|  | 13 | 13.0 | 37.6 | 13.9 | 3.2 | 9.39 | -21.62 |
|  | 14 | 9.0 | 36.4 | 13.4 | 3.2 | 8.90 | -21.46 |
|  | 15 | 3.5 | 35.7 | 13.3 | 3.1 | 8.89 | -21.09 |

| **LBSG060 M1** | **sample** | **mid-point (mm)** | **%C** | **%N** | **C/N** | **δ ^15^N (‰)** | **δ ^13^C (‰)** |
| --- | --- | --- | --- | --- | --- | --- | --- |
| anterior lobe | 1 | 44.5 | 42.1 | 15.5 | 3.2 | 8.15 | -21.07 |
|  | 2 | 39.4 | 42.6 | 15.6 | 3.2 | 7.87 | -20.90 |
|  | 3 | 35.9 | 41.9 | 15.4 | 3.2 | 7.68 | -20.90 |
|  | 4 | 32.6 | 42.0 | 15.3 | 3.2 | 7.34 | -21.13 |
|  | 5 | 26.6 | 41.7 | 15.2 | 3.2 | 7.47 | -21.30 |
|  | 6 | 20.4 | 38.4 | 13.9 | 3.2 | 7.09 | -21.13 |
|  | 7 | 16.8 | 36.9 | 13.3 | 3.2 | 7.26 | -21.23 |
|  | 8 | 13.5 | 27.5 | 10.1 | 3.2 | 7.02 | -21.36 |
|  | 9 | 10.7 | 35.3 | 13.1 | 3.1 | 7.07 | -21.33 |
|  | 10 | 8.0 | 41.4 | 15.4 | 3.1 | 6.88 | -21.20 |
|  | 11 | 4.6 | 42.8 | 15.8 | 3.2 | 6.84 | -21.13 |
|  | 12 | 1.5 | 40.1 | 14.9 | 3.1 | 6.36 | -21.06 |
| posterior lobe | 1 | 45.2 | 42.1 | 15.5 | 3.2 | 8.40 | -21.22 |
|  | 2 | 41.3 | 42.0 | 15.3 | 3.2 | 7.96 | -20.92 |
|  | 3 | 37.3 | 41.9 | 15.5 | 3.2 | 7.68 | -20.72 |
|  | 4 | 34.5 | 40.6 | 14.8 | 3.2 | 7.45 | -20.93 |
|  | 5 | 30.4 | 42.2 | 15.5 | 3.2 | 7.33 | -21.00 |
|  | 6 | 26.5 | 35.8 | 13.1 | 3.2 | 7.31 | -21.12 |
|  | 7 | 24.2 | 21.2 | 7.7 | 3.2 | 7.27 | -21.38 |
|  | 8 | 19.6 | 34.5 | 12.7 | 3.2 | 7.07 | -21.06 |
|  | 9 | 13.3 | 38.8 | 14.1 | 3.2 | 7.28 | -21.29 |
|  | 10 | 8.8 | 26.3 | 9.6 | 3.2 | 6.91 | -21.34 |
|  | 11 | 5.8 | 42.8 | 16.0 | 3.1 | 6.52 | -21.29 |
|  | 12 | 2.1 | 43.0 | 15.8 | 3.2 | 6.80 | -21.10 |


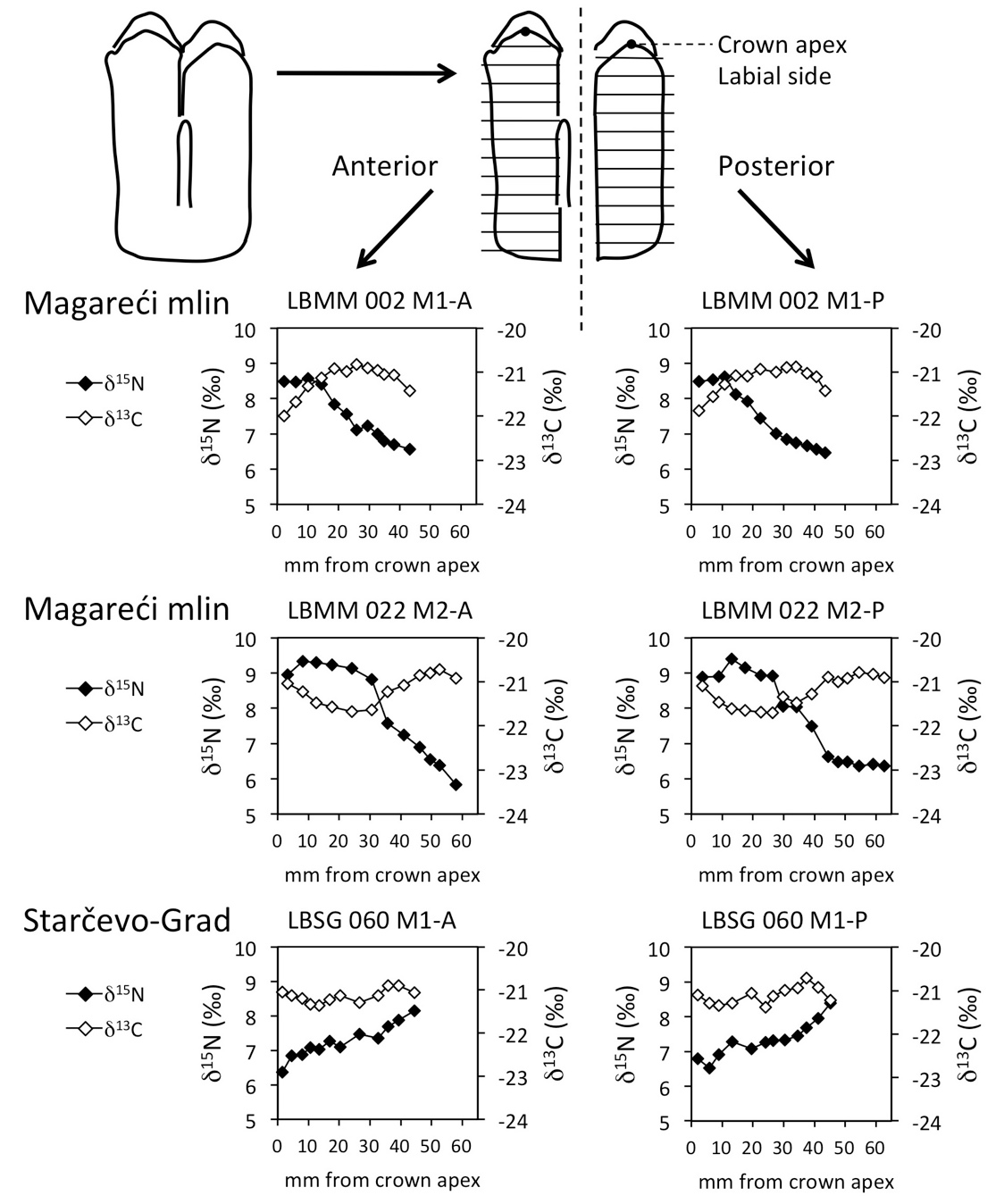


Figure S3.1: Results from sequential analysis of dentine collagen stable nitrogen (δ^15^N) and carbon (δ^13^C) isotope ratios in cattle first (m1) and second molars (m2) at Magareći Mlin and Starčevo-Grad.
