# Supplementary material for: Living off the land: Terrestrial-based diet and dairying in the farming communities of the Neolithic Balkans
Source: PLoS One. 2020 Aug 20;15(8):e0237608. doi: 10.1371/journal.pone.0237608 (PMC7444498; doi:10.1371/journal.pone.0237608)
Supplement: S4 File — (DOCX) [file pone.0237608.s004.docx]

The importance of stock herding and dairying in the Neolithic Balkans and the subsistence diversity

Supplementary Information 4: Dental calculus tables

Darko Stojanovski, Ivana Živaljević, Vesna Dimitrijević, Julie Dunne, Richard P. Evershed, Marie Balasse, Adam Dowle, Jessica Hendy, Krista McGrath, Roman Fischer, Camilla Speller, Jelena Jovanović, Emmanuelle Casanova, Timothy Knowles, Lidija Balj, Goce Naumov, Anđelka Putica, Andrej Starović, Sofija Stefanović

Table S4.1: Samples of dental calculus analysed in this study

| **Site name** | **ID** | **Tooth (FDI nomenclature)** | **Quantity of calculus used (mg)** | **Number of Identified Proteins (> 2 peptides)** |
| --- | --- | --- | --- | --- |
| Ajmana | AJ7 | T32 | 15.1/14.9 | 50* |
| Ajmana | AJ6 | T31 | 7.8/8 | 45* |
| Ajmana | AJ9 | T45 | 8.6 | 159 |
| Ajmana | AJ11 | T13 | 9.9 | 124 |
| Ajmana | AJ13 | T41 | 5.7 | 35 |
| Ajmana | AJ14* | T42 | 7.9 | 61 |
| Ajmana | AJ15* | T41 | <10 | 33 |
| Lepenski Vir | LV8 | T35 | 10.3 | 71 |
| Lepenski Vir | LV11 | T31 | 16.9 | 281 |
| Lepenski Vir | LV13 | T26 | 7.5 | 225 |
| Lepenski Vir | LV14 | T24 | 11.9 | 138 |
| Lepenski Vir | LV17 | T31 | 11.9 | 177 |
| Lepenski Vir | LV19 | T34  (possible glue) | 6.2 | 46 |
| Lepenski Vir | LV20 | T17 | 8 | 65 |
| Lepenski Vir | LV27a | T16 (possible sediment) | 5.8 | 82 |
| Lepenski Vir | LV32b | T35 | 7.8 | 80 |
| Lepenski Vir | LV54d | T17 | 11.6 | 139 |
| Lepenski Vir | LV56* | T17 | 4.8 | 57 |
| Lepenski Vir | LV61 | T21 (small fragments of tooth present) | 17.3 | 319 |
| Lepenski Vir | LV66 | T26 | 20.6 | 115 |
| Lepenski Vir | LV79ac | T17 | 7.6 | 78 |
| Lepenski Vir | LV83a | T21 (small fragments of tooth present) | 10.4 | 52 |
| Lepenski Vir | LV84* | T75, 85 | <10 | 30 |
| Lepenski Vir | LV88 | T45 | 10.7 | 96 |
| Sremski Karlovci | SK1 | T42 | 8.1 | 126 |
| Golokult Vizić | GV2/1984 | T13 | 17.6 | 102 |
| Golokult Vizić | GV3/1984 | T18 | 13.1 | 14 |
| Golokult Vizić | GV3/2003 | T31 | 28.3 | 19 |
| Sajlovo | SAJ19 | T34 (traces of calcification) | 14.4 | 98 |
| Gospođinci | GOSP O.37 | T35 | 11.5 | 56 |
| Pločnik | PL76-78 | T34 | 11.6 | 179 |
| Starčevo | STA1 | T16 | 7.6 | 55 |
| Starčevo | STV1* | T34 | 14.6 | 96 |
| Starčevo | STV2* | T84 | <10 | 64 |
| Hajdučka Vodenica | HV13 | T21 | 11.3 | 129 |
| Hajdučka Vodenica | HV14* | T32 | <10 | 28 |
| Rudnik Kosovski | RK1 | T42 | 10.2/10.5 | 23* |
| Padina | P5 | T32 | 9 | 57 |
| Padina | PD23* | T54,85 | <10 | 36 |
| Vinča | VIN IV | T16 | 8.9 | 72 |
| Vinča | VIN VI? | T14 | 9.1 | 118 |
| Vinča | VIN X | T26 | 8.1 | 21 |
| Bački Monoštor | BM1 | T35 | 10 | 112 |
| Bački Monoštor | BM3 | T23 | 8.6 | 32 |
| Gomolava | GOM7 | T13 | 7.7 | 36 |
| Gomolava | GOM21 | T16 | 9.2 | 110 |
| Gomolava | GOM22 | T31 | 11.5/11.4 | 35* |
| Gomolava | GOM25 | T14 | 7.8/8.6 | 53* |
| Gomolava | GOM18 | T48 | 13.2 | 122 |
| Popova Zemlja | PZ16 | T26 | 3.2 | 76 |
| Vlasac | VLC23* | T37 | <10 | 39 |

* indicates the first nine samples analysed as part of a pilot assessment of overall protein preservation.

Table S4.2: Summary of dental calculus total protein identifications and protein family identifications

| **Skeleton** | **Extraction Material*** | **Total # identified proteins  (>2 PSMs)** | **Contaminant protein family** | **Mammal protein family** | **Fish protein family** | **Plant protein family** | **Microbial protein family** |
| --- | --- | --- | --- | --- | --- | --- | --- |
| AJ7 | S | 51 | 6 | 4 | 0 | 0 | 28 |
| AJ7 | S&P | 40 |  |  |  |  |  |
| AJ6 | S | 19 | 9 | 7 | 0 | 0 | 28 |
| AJ6 | S&P | 59 |  |  |  |  |  |
| AJ9 | S&P | 159 | 14 | 11 | 0 | 0 | 83 |
| AJ11 | S&P | 124 | 14 | 7 | 0 | 0 | 65 |
| AJ13 | S&P | 35 | 9 | 3 | 0 | 0 | 14 |
| AJ14 | S&P | 61 | 14 | 10 | 0 | 0 | 11 |
| AJ15 | S&P | 33 | 13 | 1 | 0 | 0 | 3 |
| LV8 | S&P | 71 | 12 | 13 | 0 | 0 | 33 |
| LV11 | S&P | 281 | 15 | 16 | 1 | 1 | 180 |
| LV13 | S&P | 225 | 14 | 8 | 0 | 0 | 149 |
| LV14 | S&P | 138 | 16 | 13 | 0 | 0 | 79 |
| LV17 | S&P | 177 | 16 | 24 | 1 | 1 | 91 |
| LV19 | S&P | 46 | 16 | 4 | 0 | 0 | 16 |
| LV20 | S&P | 65 | 20 | 7 | 0 | 0 | 14 |
| LV27a | S&P | 82 | 20 | 9 | 0 | 0 | 36 |
| LV32b | S&P | 80 | 11 | 8 | 0 | 0 | 40 |
| LV54d | S&P | 139 | 10 | 6 | 0 | 0 | 88 |
| LV56 | S&P | 57 | 17 | 10 | 0 | 0 | 9 |
| LV61 | S&P | 319 | 16 | 11 | 0 | 0 | 197 |
| LV66 | S&P | 115 | 16 | 6 | 0 | 0 | 68 |
| LV79ac | S&P | 78 | 13 | 6 | 0 | 0 | 41 |
| LV83a | S&P | 52 | 14 | 5 | 0 | 0 | 25 |
| LV84 | S&P | 30 | 11 | 0 | 0 | 0 | 5 |
| LV88 | S&P | 96 | 12 | 7 | 0 | 0 | 51 |
| SK1 | S&P | 126 | 15 | 8 | 0 | 0 | 75 |
| GV2/1984 | S&P | 102 | 15 | 9 | 0 | 0 | 52 |
| GV3/1984 | S&P | 14 | 9 | 3 | 0 | 0 | 0 |
| GV3/2003 | S&P | 19 | 10 | 2 | 0 | 0 | 0 |
| SAJ19 | S&P | 98 | 18 | 9 | 0 | 0 | 46 |
| GOSP O.37 | S&P | 56 | 13 | 9 | 0 | 0 | 20 |
| PL76-78 | S&P | 179 | 23 | 28 | 0 | 2 | 78 |
| STA1 | S&P | 55 | 21 | 6 | 0 | 0 | 1 |
| STV1 | S&P | 96 | 8 | 11 | 0 | 3 | 47 |
| STV2 | S&P | 64 | 13 | 19 | 0 | 0 | 19 |
| HV13 | S&P | 129 | 14 | 17 | 0 | 0 | 67 |
| HV14 | S&P | 28 | 9 | 2 | 0 | 0 | 3 |
| RK1 | S | 32 | 11 | 2 | 0 | 1 | 1 |
| RK1 | S&P | 4 |  |  |  |  |  |
| P5 | S&P | 8 | 21 | 4 | 0 | 0 | 25 |
| PD23 | S&P | 36 | 15 | 0 | 0 | 0 | 4 |
| VIN IV | S&P | 72 | 14 | 10 | 0 | 0 | 27 |
| VIN VI? | S&P | 118 | 9 | 12 | 0 | 0 | 62 |
| VIN X | S&P | 21 | 8 | 2 | 0 | 0 | 4 |
| BM1 | S&P | 112 | 21 | 16 | 0 | 1 | 39 |
| BM3 | S&P | 32 | 13 | 3 | 0 | 0 | 7 |
| GOM7 | S&P | 36 | 10 | 5 | 0 | 1 | 16 |
| GOM21 | S&P | 110 | 25 | 14 | 0 | 0 | 38 |
| GOM22 | S | 8 | 10 | 3 | 0 | 0 | 11 |
| GOM22 | S&P | 29 |  |  |  |  |  |
| GOM25 | S | 24 | 8 | 3 | 0 | 0 | 33 |
| GOM25 | S&P | 44 |  |  |  |  |  |
| GOM18 | S&P | 122 | 12 | 7 | 0 | 0 | 73 |
| PZ16 | S&P | 76 | 18 | 6 | 0 | 0 | 36 |
| VLC23 | S&P | 39 | 13 | 5 | 0 | 0 | 4 |
|  |  |  |  |  |  |  |  |
| eBK1+2 | Blank | 31 | 14 | 4 | 0 | 0 | 5 |
| eBK3 | Blank | 49 | 20 | 0 | 0 | 0 | 9 |
| eBK5 | Blank | 63 | 25 | 0 | 0 | 0 | 16 |
| eBK6 | Blank | 53 | 19 | 0 | 0 | 0 | 12 |

* S indicates ‘Supernatant only’ while S&P indicates ‘Supernatant & Pellet’ used for protein extraction.

Table S4.3: List of putative dietary proteins identified with the dental calculus samples

| **Skeleton (% contaminant protein families)** | **Protein** | **Peptide sequence** | **Mascot Ion Score** | **Expect Score** | **Peptide Taxonomic Assignment** |
| --- | --- | --- | --- | --- | --- |
| GOM7 (31%) | Alpha-amylase/trypsin inhibitor CM3 | K.LYCCQELAEISQQCR.C | 36 | 0.0014 | *Triticum* |
|  |  | R.LLVAPGQCNLATIHNVR.Y | 32 | 0.0015 | *Triticeae* |
|  | Beta-amylase | R.NIEYLTLGVDNQPLFHGR.S + Deamidated (NQ) | 73 | 7.70E-07 | *Hordeum* |
|  | Gamma-gliadin | R.APFASIVAGIGGQ.- | 60 | 0.001 | *Triticeae* |
|  | Glutenin, high molecular weight subunit | R.CCQQLRDVSAK.C | 51 | 0.00077 | *Triticeae* |
| BM1  (27%) | Glutelin type-A 2 | R.GLSLLQPYASLQEQEQGQMQSR.E + Oxidation (M) | 50 | 0.00026 | *Oryza* |
|  |  | R.ALPTDVLANAYR.I | 49 | 0.0055 | *Oryzeae* |
| LV11  (7%) | Vitellogenin | K.LLPVFGTAAAALPLR.V | 56 | 9.60E-06 | *Salmoninae* |
|  |  | R.VQADAVLALR.N | 51 | 0.0061 | *Salmoninae* |
|  |  | R.VQADAVLALR.N + Deamidated (NQ) | 78 | 2.10E-05 | *Salmoninae* |
|  |  | R.IGAAASAFYINDAATLFPR.T + Deamidated (NQ) | 25 | 0.0084 | *Oncorhynchus* |
|  |  | R.TYFAGAAADVLEVGVR.T | 45 | 0.00071 | *Salmoninae* |
| LV17 (12%) | Vitellogenin | R.VQADAVLALR.N | 52 | 0.0055 | *Salmoninae* |
|  |  | R.TYFAGAAADVLEVGVR.T | 43 | 0.0017 | *Salmoninae* |
|  | Allergen Ara h 1, clone P17 | R.DQSSYLQGFSR.N | 36 | 0.0048 | *Arachis* |
|  |  | R.VLLEENAGGEQEER.G | 34 | 0.002 | *Arachis* |
|  | Alpha-S1-casein | R.FFVAPFPEVFGKEK.V | 35 | 0.0016 | *Bovinae* |
| STA1 | Patatin-like protein 7 | K.RIDEQSNFEK.L + Propionamide (K) | 33 | 0.046 | *Brassicaceae* |
|  |  | K.RIDEQSNFEK.L + Deamidated (NQ); Propionamide (K) | 30 | 0.069 |  |
|  |  | R.IDEQSNFEK.L + Propionamide (K) | 44 | 0.0032 |  |
|  |  | R.IDEQSNFEK.L + Deamidated (NQ); Propionamide (K) | 26 | 0.034 |  |
|  |  | R.IDEQSNFEK.L + 2 Deamidated (NQ); Propionamide (K) | 30 | 0.077 |  |
| PL76_78 (17%) | Beta-amylase | R.NIEYLTLGVDNQPLFHGR.S + Deamidated (NQ) | 32 | 0.0027 | *Hordeum* |
|  |  | R.YDPTAYNTILR.N | 45 | 0.0051 | *Pooideae* |
|  | Gamma-gliadin (Fragment) | R.ILPTMCSVNVPLYR.T | 46 | 7.80E-05 | *Triticeae* |
|  |  | R.ILPTMCSVNVPLYR.T + Oxidation (M) | 26 | 0.004 | *Triticeae* |
|  | Glutenin, high molecular weight subunit 12 | R.ELQESSLEACR.Q | 53 | 0.00016 | *Triticeae* |
|  | Serine/threonine-protein phosphatase BSL1 | R.YLVTVTGNDGKR.A + Deamidated (NQ); Propionamide (K); Propionamide (N-term) | 39 | 0.0037 | *Pentapetalae* |
| RK1* (73%) | Glutelin type-B 2 | R.VIQPQGLLVPR.Y | 41 | 0.003 | *Oryza* |
|  |  | R.ALPVDVVANAYR.I | 38 | 0.0027 | *Oryzeae* |
|  | Glutelin type-A 3 | R.ALPDDVVANAYR.I | 40 | 0.0027 | *Oryzeae* |
|  | Glutelin type-A 2 | R.ALPTDVLANAYR.I | 57 | 0.00015 | *Oryza* |
| SAJ19 (25%) | Beta-lactoglobulin | R.LSFNPTQLEEQCHI.- | 78 | 9.70E-08 | *Bovinae* |

*Represents protein identifications from merged supernatant and supernatant & pellet datasets. Shaded cells represent proteins detected with only a single peptide support.

Note: Protein MS/MS identification is highly dependent on reference databases, and for many plants (and non-commercially important animals) UniProt is grossly under-characterized, with entries being dominated by commercially relevant species. Even when ‘dietary’ taxa are represented in protein reference databases, they may be biased towards particular classes of proteins or biological pathways, for example photosynthetic genes and proteins, or allergenic proteins. As many proteins and peptides are conserved across related taxa, spectra are likely to be matched to the taxonomically closest species in the database and may not necessarily reflect the exact source species. For example, peptides identified to the rice genus (Oryza) may represent conserved proteins within *Poaceae* more broadly; likewise, peptides identified as Arachis may represent conserved proteins within *Faboideae*. Nevertheless, considering the relatively high proportion of proteins derived from human skin contaminants, and the high proportion of contaminant protein families overall, the possibility that food derived proteins may have been transferred as contaminants to the dental calculus samples as a result of conservation treatments (e.g., glues, consolidants) and/or previously handling cannot be discounted.
