# Supplementary material for: Living off the land: Terrestrial-based diet and dairying in the farming communities of the Neolithic Balkans
Source: PLoS One. 2020 Aug 20;15(8):e0237608. doi: 10.1371/journal.pone.0237608 (PMC7444498; doi:10.1371/journal.pone.0237608)
Supplement: S5 File — (DOCX) [file pone.0237608.s005.docx]

The importance of stock herding and dairying in the Neolithic Balkans and the subsistence diversity

Supplementary Information 5: Materials and Methods

Darko Stojanovski, Ivana Živaljević, Vesna Dimitrijević, Julie Dunne, Richard P. Evershed, Marie Balasse, Adam Dowle, Jessica Hendy, Krista McGrath, Roman Fischer, Camilla Speller, Jelena Jovanović, Emmanuelle Casanova, Timothy Knowles, Lidija Balj, Goce Naumov, Anđelka Putica, Andrej Starović, Sofija Stefanović

1. Organic residue analyses and CSRA

The pottery comes from the Neolithic layers of four different sites (Fig. 1: sites 1, 9, 11 and 16). Three of the sites, Magareći Mlin, Starčevo-Grad and Rutonjina Greda, are in Vojvodina (North Serbia). This area represents the southern flanks of the great Pannonian (Hungarian) Plain, with a pronounced continental climate. The Vrbjanska Čuka site is in the Pelagonia Plain in Macedonia. This isolated valley in the south-central part of the Balkan Peninsula has some influences from the Mediterranean coast with hot and dry summers, but also experiences continental conditions with cold and snowy winters. Radiocarbon dates are available for three of the sites, 13 from previous publications and five obtained for the purposes of this project (Fig. S5.1). Overall, the three dated sites are contained within the first half of the 6^th^ millennium calBC, with Starčevo-Grad following in time after Magareći Mlin and Vrbjanska Čuka, with significant overlap.

A total of 213 potsherds from the four sites were submitted for organic residue analysis (Supplementary Information 1 (from here on, all references to the Supplementary Information will be abbreviated as SI); Table S5.1). All but 23 sherds contained morphological features which allowed them to be classified into vessel types (SI 2). Three general types of pottery vessels were recognized throughout the Neolithic collections from the four sites: dishes (T200), bowls (T300) and jars (T400) (SI1 and 2). The dishes comprise an open type of vessel, bowls are semi-open with an easily accessible interior, and the jars have a constricted opening. The general narrative in archaeology (rarely justified) is that dishes are used for serving and consumption of food, bowls are cooking vessels, and the jars are storage containers. Within each of these groups, based on certain features and attributes, several sub-categories are identified (Fig. S5.2; SI 2). In addition to the described pottery vessels, a fourth group of ceramic objects, the so-called altars (T500; SI 2, Fig. S2.13), was also sampled.


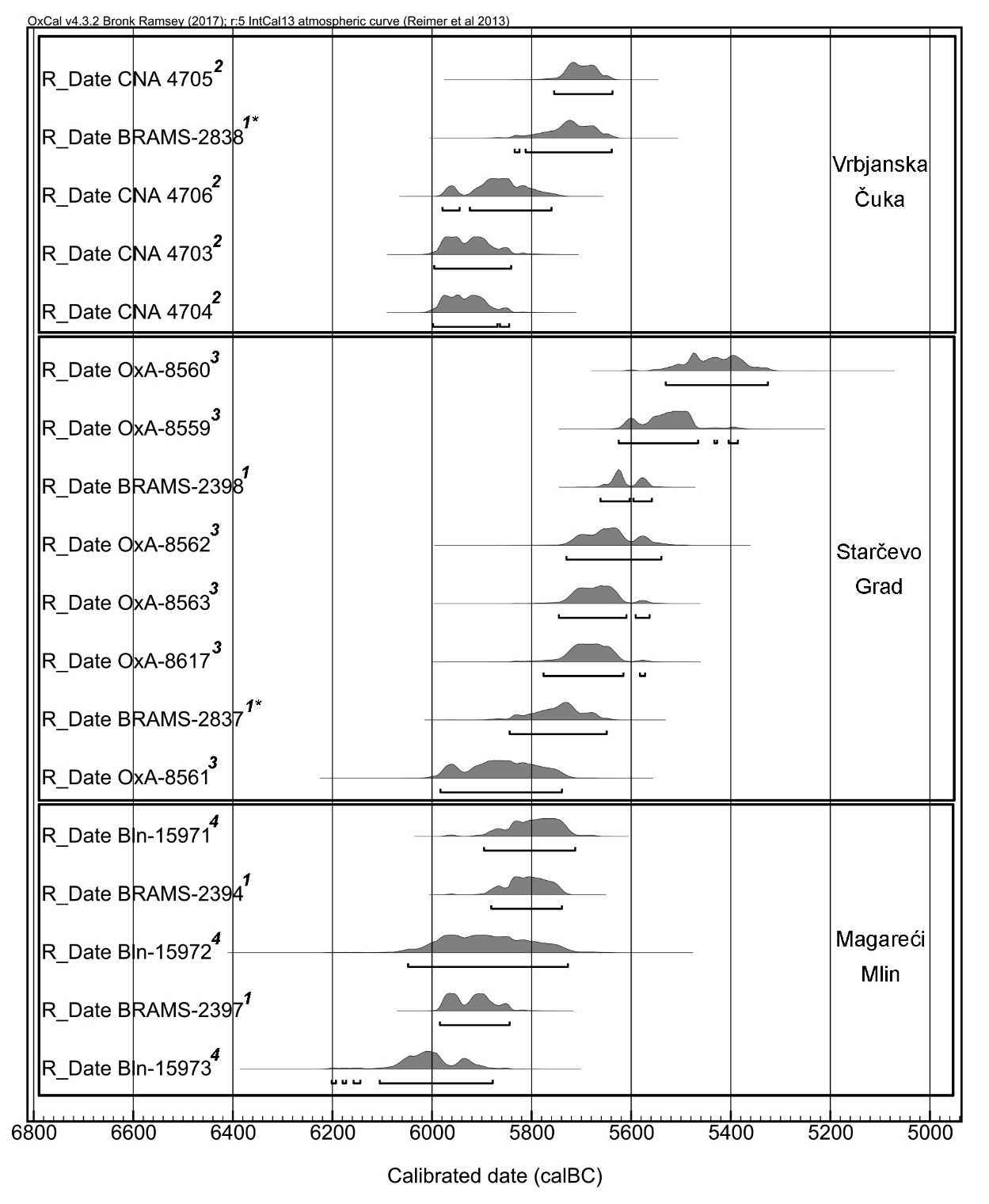


Figure S5.1 Calibration multiple plot of radiocarbon dates available for the sites of Vrbjanska Čuka, Starčevo Grad and Magareći Mlin (1-this study, cattle mandible; 1*-this study, compound specific date on organic residue; 2-[1]; 3-[2]; 4-[3]


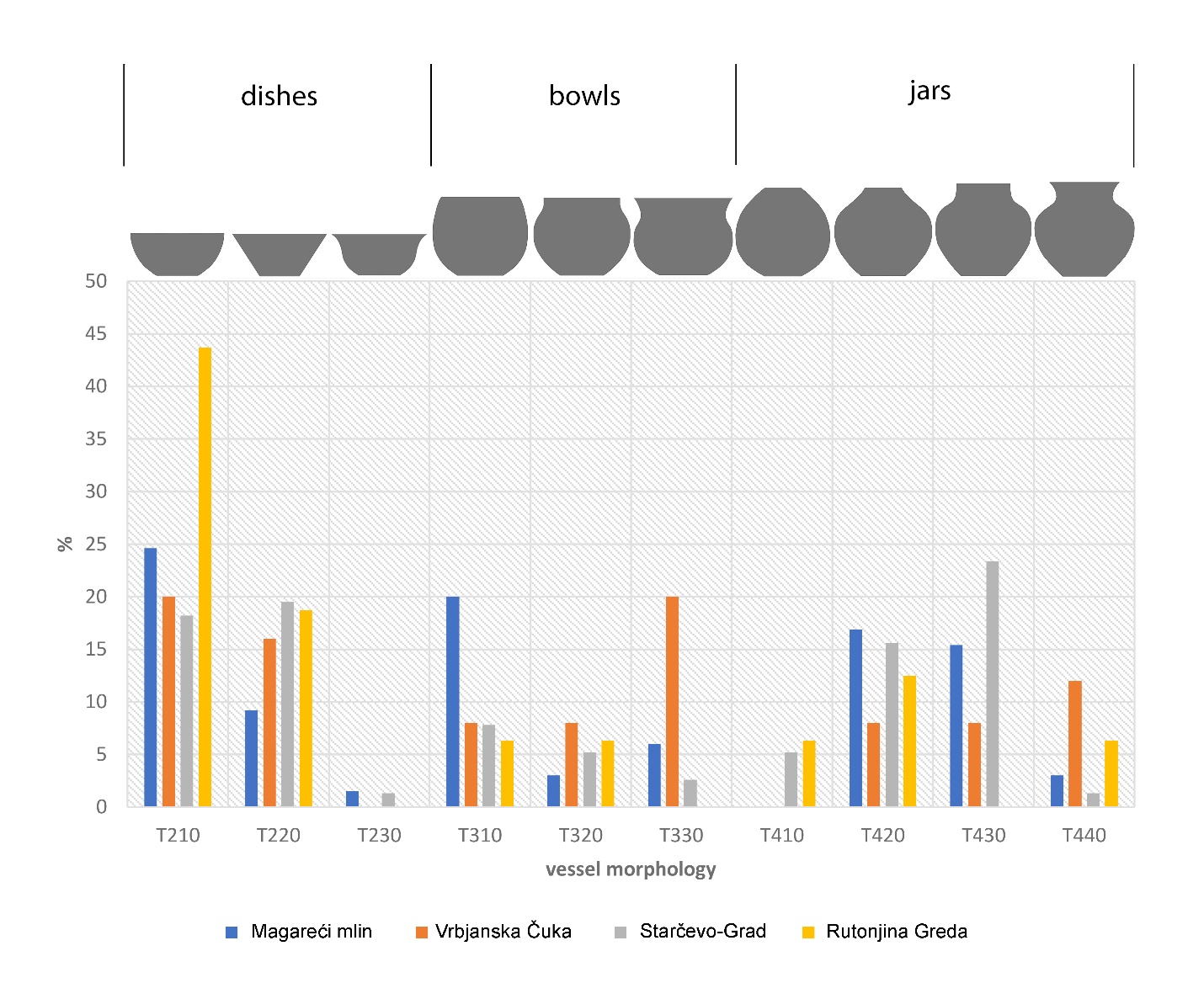


Figure S5.2 Relative distribution of different pottery shapes within each of the four sample collections

At three of the sites (Magareći Mlin, Starčevo-Grad and Rutonjina Greda), more than 60 % of the rims (i.e. fragments with identifiable morphology) from the entire excavated assemblage were sampled, thus in terms of general typology our results are thought to reflect overall trends in the entire Neolithic pottery assemblage. However, until a complete typological assessment is carried out, especially in terms of statistical representation, we must stress that these results reflect only the trends in our sample group.

Table S5.1 Summary table of organic residue pottery samples and results

| Site | Region | Chronology | Type | Samples (n) | Samples containing archaeological organic residue | | Terrestrial animal fats (n) | Beeswax  (n) | Plant oils and fats  (n) |
| --- | --- | --- | --- | --- | --- | --- | --- | --- | --- |
|  |  |  |  |  | n | % |  |  |  |
| Vrbjanska Čuka | Pelagonia, Macedonia, South-central Balkan | 5900-5500 calBC | dishes | 9 | 2 | 22 | 2 | 0 | 1 |
|  |  |  | bowls | 9 | 3 | 33 | 3 | 0 | 1 |
|  |  |  | jars | 8 | 2 | 25 | 1 | 0 | 1 |
|  |  |  | altars | 0 | 0 | 0 | 0 | 0 | 0 |
|  |  |  | unknown | 1 | 0 | 0 | 0 | 0 | 0 |
|  |  |  | **total** | **27** | **7** | **26** | **6** | **0** | **3** |
| Starčevo-Grad | Vojvodina, Serbia, northern Balkan | 5700-5500 calBC | dishes | 31 | 5 | 16 | 6 | 1 | 2 |
|  |  |  | bowls | 12 | 2 | 17 | 2 | 2 | 0 |
|  |  |  | jars | 36 | 14 | 39 | 14 | 0 | 0 |
|  |  |  | altars | 1 | 0 | 0 | 0 | 0 | 0 |
|  |  |  | unknown | 13 | 6 | 46 | 6 | 1 | 0 |
|  |  |  | **total** | **93** | **30** | **32** | **28** | **4** | **2** |
| Magareći Mlin | Vojvodina, Serbia, northern Balkan | 6100-5700 calBC | dishes | 23 | 7 | 26 | 6 | 1 | 0 |
|  |  |  | bowls | 19 | 5 | 21 | 4 | 1 | 0 |
|  |  |  | jars | 23 | 4 | 17 | 4 | 0 | 0 |
|  |  |  | altars | 3 | 0 | 0 | 0 | 0 | 0 |
|  |  |  | unknown | 8 | 0 | 0 | 0 | 0 | 0 |
|  |  |  | **total** | **76** | **15** | **21** | **14** | **2** | **0** |
| Rutonjina Greda | Vojvodina, Serbia, northern Balkan | 5900-5500 calBC | dishes | 10 | 2 | 20 | 2 | 0 | 0 |
|  |  |  | bowls | 2 | 1 | 50 | 1 | 0 | 0 |
|  |  |  | jars | 4 | 0 | 0 | 0 | 0 | 0 |
|  |  |  | altars | 0 | 0 | 0 | 0 | 0 | 0 |
|  |  |  | unknown | 1 | 0 | 0 | 0 | 0 | 0 |
|  |  |  | **total** | **17** | **3** | **18** | **3** | **0** | **0** |
| Total | | | | 213 | 55 | 26 | 51 | 5 | 5 |

Lipid analysis and interpretations were performed using established protocols described in detail in earlier publications [4]. Briefly, ~2 g of potsherd was sampled, and surfaces cleaned with a modelling drill to remove exogenous lipids. The cleaned powder was crushed in a solvent-washed mortar and pestle and weighed into a furnaced culture tube (I). All solvents used were HPLC grade (Rathburn) and the reagents were analytical grade (typically > 98% of purity). An internal standard, typically 20 µg, was added to enable quantification of the lipid extract (*n*-tetratriacontane; Sigma Aldrich Company Ltd). Following the addition of 5 mL of H_2_SO_4_/MeOH 2 - 4% (δ^13^C measured), the culture tubes were placed on a heating block for 1 h at 70 °C, mixing every 10 min. Once cooled, the methanolic acid was transferred to test tubes and centrifuged at 2500 rpm for 10 min. The supernatant was then decanted into another furnaced culture tube (II) and 2 mL of dichloromethane extracted double distilled water was added. In order to recover any lipids not fully solubilised by the methanol solution, 2 x 3 mL of *n*-hexane was added to the extracted potsherds contained in the original culture tubes, mixed well and transferred to culture tube II. The extraction was transferred to a clean, furnaced 3.5 mL vial and blown down to dryness. Following this, 2 x 2 mL *n*-hexane was added directly to the H_2_SO_4_/ MeOH solution in culture tube II and whirl-mixed to extract the remaining residues. This was transferred to the 3.5 mL vials and blown down under a gentle stream of nitrogen until a full vial of *n*-hexane remained. Aliquots of the extracts (containing fatty acid methyl esters, FAME’s) were derivatised using N,O-bis(trimethylsilyl)trifluoroacetamide (BSTFA) containing 1 % *v/v* trimethylchlorosilane (TMCS; Sigma Aldrich Company Ltd.; 20 μL; 70°C, 1 h). Excess BSTFA was removed under nitrogen and the extract was dissolved in *n*-hexane for analysis by gas chromatography (GC), GC–mass spectrometry (GC–MS) and GC–combustion–isotope ratio MS (GC–C–IRMS).

Further analysis was carried out using the solvent extraction method. An internal standard was added to the sherd powder and the extracts underwent ultrasonication (chloroform/methanol 2:1 *v/v*, 30 min, 2 x 10ml). The solvent was then evaporated under a gentle stream of nitrogen to obtain the total lipid extract (TLE). Aliquots of the TLE were trimethylsilylated using BSTFA (20 µL, 70° C, 1 h), diluted with *n*-hexane and submitted to analysis by high-temperature-GC (HTGC) and HTGC-MS.

All FAMEs initially underwent GC analysis using a gas chromatograph (GC) fitted with a HT non-polar column (DB1-HT; 100% dimethylpolysiloxane, 15 m x 0.32 mm i.d., 0.1 μm film thickness). The carrier gas was helium and the temperature programme comprised a 50°C isothermal hold followed by an increase to 350°C at a rate of 10°C min−1 followed by a 10 min isothermal hold. A procedural blank (no sample) was prepared and analysed alongside every batch of samples. Further compound identification was accomplished using GC-MS. FAMEs were then introduced by autosampler onto a GC-MS fitted with a non-polar column (100% dimethyl polysiloxane stationary phase; 60 m x 0.25 mm i.d., 0·1 μm film thickness). The instrument was a ThermoFinnigan single quadrupole TraceMS run in EI mode (electron energy 70 eV, scan time of 0.6 s). Samples were run in full scan mode (m/z 50–650) and the temperature programme comprised an isothermal hold at 50°C for 2 min, ramping to 300°C at 10° min-1, followed by an isothermal hold at 300°C (15 min). Data acquisition and processing were carried out using the HP Chemstation software (Rev. C.01.07 (27), Agilent Technologies) and Xcalibur software (version 3.0). Peaks were identified on the basis of their mass spectra and GC retention times, by comparison with the NIST mass spectral library (version 2.0).

Carbon isotope analyses by GC-C-IRMS were also carried out using a GC Agilent Technologies 7890A coupled to an Isoprime 100 (EI, 70eV, three Faraday cup collectors m/z 44, 45 and 46) via an Isoprime GC5 combustion interface with a CuO and silver wool reactor maintained at 850°C. Instrument accuracy was determined using an external FAME standard mixture (C11, C13, C16, C21 and C23) of known isotopic composition. Samples were run in duplicate and an average taken. The δ13C values are the ratios 13C/12C and expressed relative to the Vienna Pee Dee Belemnite, calibrated against a CO2 reference gas of known isotopic composition. Instrument error was ± 0.3‰. Data processing was carried out using Ion Vantage software (version 1.6.1.0, IsoPrime).

Compound-specific radiocarbon analyses (CSRA) of fatty acids were performed following published procedures [5,6]. Briefly 2-20g of sherds were sampled and extracted with a methanolic/sulphuric acid solution (4 % *v/v*, 3x8 mL, 70^o^C, 3x1h). The solution was centrifuged (2500 rpm, 10 min) and extracted with *n*-hexane (4 x 5 mL). The extracted FAMEs were dissolved in ca. 180 µl to obtain a solution at 5 µg.µl^-1^ of C before injection in a preparative capillary gas chromatography instrument (pcGC). The GC is equipped with a 100% poly(dimethyl siloxane) stationary phase column (Rxi-1ms, 30 m x 0.53 mm i.d., 1.5 μm film thickness, Thames Restek, UK). The GC oven was held at 50 °C for 2 min, and then increased to 200 °C at a rate of 40 °C min^-1^, then increased again to 270 °C at a rate of 10 °C min^-1^ and finally to 300 °C at a rate of 20 °C min^-1^, where it was held for 8.75 min with the use of He as carrier gas at a constant pressure of 10 psi. The C_16:0_ and C_18:0_ FAMEs (99%) were transferred to a preparative fraction collector heated at 320 °C through a transfer line (320°C) and isolated in individual traps using a solventless trapping system (Casanova et al 2018). Isolated FAMEs were transferred to an Al capsule and combusted in an elemental analyser (EA) linked to an Automated Graphitisation System (AGE3). The CO_2_ generated by combustion was reduced to graphite on activated iron catalyst (580°C, 2h). Radiocarbon measurements were performed at the Bristol Accelerator Mass Spectrometry (BRAMS) facility. Data analyses and reduction was performed on BATs software.

1. Archaeozoological analyses and mortality profiles

Faunal assemblages analysed and discussed in this study originate from Early Neolithic contexts at the sites of Vrbjanska Čuka, Starčevo-Grad and Magareći Mlin, i.e. from sites which were also tested for the presence of dairy fats in ceramic vessels. Animal remains from Vrbjanska Čuka were collected by hand and flotation during 2016‒2018 excavations of the site [7,8], and those from Magareći Mlin were collected manually during 1986‒1989 excavations campaigns [9]. Both assemblages were analysed by two of the authors of this study (IŽ and VD). In case of Starčevo-Grad, the information on the taxonomic composition was taken from Clason’s [10] study of the faunal sample hand-collected in 1932, whereas our study of cattle mortality profiles and isotopic analyses of cattle teeth were undertaken on a sample hand-collected during 2003‒2004 and 2007 [11,12]. Although the faunal assemblages from Magareći Mlin and Starčevo-Grad were collected exclusively by hand and are therefore biased, their taxonomic composition was relatively similar to the Vrbjanska Čuka sample (collected by hand and flotation) in terms of the prevalence of domestic ruminants. Cattle mortality profiles were constructed using lower teeth and mandibles from Starčevo (MNI = 14) and Magareći Mlin (MNI = 15). All specimens included in the mortality profile construction originated from different individuals (determined on the basis of tooth age stage and laterality), i.e. represented the minimal number of individuals. The number of mandible and teeth remains from Vrbjanska Čuka (MNI = 4) was insufficient for mortality profile construction. Age classes are defined following Gillis, 2012 (table 4.12; Fig. 4.25), and the age estimation is based on criteria for dental development and wear stages proposed by Brown *et al*. [13], Ducos [14], Grant [15] and Gillis [16].

1. Isotopic analyses of cattle dentine collagen

On the basis of the eruption stage and quality of preservation, teeth from two cattle mandibles from Magareći Mlin (m1 from left mandible LBMM002 and m2 from right mandible LBMM022) and m1 from right mandible LBSG060 from Starčevo-Grad were selected for the analyses of nitrogen (ẟ^15^N) and carbon (ẟ^13^C) isotope ratios of dentine collagen, in order to reconstruct weaning stages. Both mandibles from Magareći Mlin originated from Dwelling 3 and were dated to the first half of the 6^th^ millennium calBC (Fig. S5.1: BRAMS-2394, BRAMS-2397 andBRAMS-2398).

The left mandible LBMM002 from Magareći Mlin contained the complete milk teeth row and the erupting first permanent molar (selected for isotopic analyses). The milk teeth were well preserved and moderately worn (tooth wear stage of d4 “j”, *cf*. [15]). The crown of the first permanent molar was fully formed, and in the exo-gingival stage of eruption (*cf*. [16], Fig. 4.22). Consequently, it could be determined that the age of this animal was less than 6 months (*cf*. [16], Fig. 4.25), i.e., it was attributed to the age group of 4‒6 months (*cf*. [16], Table 4.12).

The other specimen from Magareći Mlin was a fragmented right mandible LBMM022, with a p3 germ, fragmented d4, m1 in wear and m2 half erupted (selected for isotopic analyses). The crown of p3 was complete and probably in the stage V (visible in the crypt) (*cf*. [15]), however this could not be observed directly due to the damaged alveolar bone. The anterior lobe of d4 was broken and the middle and posterior lobes were preserved only partially, therefore it was only possible to determine that its attrition corresponds at least to the wear stage „f“(*cf*. [15]). According to the eruption stage of the second molar, the age of this animal was less than 15 months (*cf*. [16], Fig. 4.25) and the age group 1‒2 years (*cf*. [16], Table 4.12). This is in accordance with the developmental stage of the third premolar. The crown of this tooth is fully formed between the 1^st^ and 2^nd^ year, and it emerges in the oral cavity only after the 2^nd^ year of age [13].

The right mandible LBSG060 from Starčevo-Grad contained the complete milk teeth row and the erupting first permanent molar (selected for isotopic analyses). All teeth were well preserved. The last milk molar was in wear (tooth wear stage „j“, *cf*. [15]), whereas the first permanent molar was in the exo-gingival stage of eruption (*cf*. [16], Fig. 4.22). Consequently, the mandible belonged to an animal less than 6 months old (*cf*. [16], Fig. 4.25), i.e. to the age group of 4‒6 months (*cf*. [16], Table 4.12).

Teeth were extracted from the mandibular bone and decalcified in EDTA (0.5 M, pH=7.4) at room temperature with solution renewed every two or three days (when effervescence had stopped). Sequential sampling of 3-4 mm sections of the collagen isomorph was performed using a scalpel. The sampling proceeded from the latest formed part of the crown/root to the apex of the crown. Each sample is located in the tooth using the distance from its distal margin to the apex of tooth crown on the labial side. A double sampling was performed on the anterior and the posterior lobes of each molar in order to test for the preservation of the biogenic isotopic signals. The sampled pieces were soaked individually in distilled water and rinsed daily to remove EDTA. After two weeks, the samples were homogenized by solubilization (HCl 10^‒2^ M, 100°C, 17 h), filtered and freeze-dried.

Collagen aliquots weighing around 300-350 μm were analysed in an elemental analyser (Thermo FlashEA 2000) interfaced with an isotope ratio mass spectrometer (Delta V Advantage). The analytical precision in each run, estimated from repeated analysis of an alanine standard (7 to 15 per run), ranges from 0.05 ‰ to 0.09 ‰ for δ^15^N values and from 0.03 ‰ to 0.1 ‰ for δ^13^C values. Over the course of these dentine collagen analyses, the alanine standard (calibrated against the IAEA 600 and IAEA NO3 international standards) gave a mean δ^15^N value of 0.79 ± 0.06‰ and a mean δ^13^C value of -21.94 ± 0.09‰ (N = 31; expected values 0.59‰ and -22.16‰ respectively).

1. Human dental calculus

A total number of 52 samples of dental calculus from 51 individuals recovered from 16 different Mesolithic/Neolithic archaeological sites in the Balkans were selected for analyses (Table S4.1). We sampled only supragingival calculus, since this type of calculus is mostly connected to diet. Before any sampling, we photo-documented the calculus deposits. The sampling was carried out with a clean dental curette while holding the sample over a creased weighing paper underlain by aluminium foil, ideally prised off as chunks, rather than scraped off as powder in so far as possible. In order to prevent any contamination, the sampling was done in the clean room facilities (class ISO 5) of the BioSense Institute in Novi Sad, Serbia. Kimtech Science purple nitrile gloves were used during sampling. After sampling, we weighed each sample and we photographed the impact of sampling on the surviving calculus deposits. The sampled fragments of dental calculus were analysed within the Ancient Proteomics Laboratory in the BioArCh Centre at the University of York.

Up to 28 mg of dental calculus was used for protein extraction (Table S4.1). To remove proteins from the outermost layer of the deposit, which are more likely to derive from contaminants, dental calculus was rotated for 5 minutes in 500 uL of 0.5M EDTA. The samples were centrifuged, and the supernatant removed and set aside. The dental calculus was then rotated at room temperature in a solution of EDTA and SDS (0.25-0.5M EDTA, 2-4% SDS) for 5-6 days until completely decalcified.

Proteins were extracted using a Gel-Aided Sample Preparation (GASP) protocol based on Fischer and Kessler [17], modified for ancient mineralized samples; for all extraction batches, blank extractions were conducted alongside the ancient samples to monitor for laboratory and reagent contamination. First, we conducted an initial pilot assessment of protein preservation on nine dental calculus samples (Table S4.1). Protein extraction for these samples followed the extraction protocol developed in Hendy et al. (2018), whereby proteins were extracted from the decalcified pellet and only 50 of the 1000 μL of supernatant.

Following this pilot assessment, we attempted to optimize protein recovery by increasing the quantity of supernatant included in protein extraction. Two different extraction approaches were initially tested on five samples: RK1, GOM22, GOM25, AJ6 and AJ7. The first approach extracted proteins solely from the complete supernatant portion of the demineralised calculus, while the second approach extracted proteins from both the pellet and the complete supernatant portion.

For the supernatant extractions, 100 μL 1M DTT was added to the demineralised calculus samples, followed by incubation at 70°C for 20 min. The Eppendorf tubes were centrifuged at 13,000 RPM for 2 minutes and the 1 mL of supernatant transferred to an Amicon Ultra-4centrifugal filter unit (10 kDa NMWL) and centrifuged until concentrated to ~150 μL. The retentate was transferred to a new 2 mL Eppendorf tube for protein extraction using GASP. In the second preparation method, 100 μL of 1M DTT was added directly to the supernatant & pellet, followed by incubation at 70°C for 20 min. The supernatant was then transferred to an AmiconUltra-4 centrifugal filter unit with 10 kDaNMWL, centrifuged until concentrated to ~150 μL, and returned to the 2 mL Eppendorf tube with the pellet. Subsequent protein extractions for both approaches followed the GASP method used on the nine pilot samples and outlined in Hendy *et al.*[18]. Briefly, for all samples,150 uL of Proto-Gel was added to the supernatant and pellet fractions, and gently resuspended to mix. The gel was polymerized by adding 8 μL of TEMED, followed by 8 μL 10% Ammonium Persulfate (APS), and the polymerized gel was then shredded to increase the surface area. The gel was fixed with a methanol/water/acetic acid solution (50/40/10). The solution was centrifuged, the supernatant discarded, and 1 mL of acetonitrile added to dehydrate the gel pieces. A series of washing and drying cycles were performed using 6 M urea (twice) and 0.05M ammonium bicarbonate (once), followed in each case by acetonitrile to dehydrate the gel and facilitate the removal of the supernatant. Samples were incubated overnight at 37°C in 300 μL of ammonium bicarbonate (0.05 M) and 2.5 μg trypsin.

Digested peptides were extracted by dehydrating the gel pieces in 300 μL acetonitrile, followed by 300 μL of 5% formic acid solution to extract acidic peptides, then by a further 600 μL of acetonitrile. Extracted peptides were dried down in a centrifugal evaporator and desalted using Millipore Zip-Tips prior to nano liquid-chromatography tandem mass-spectrometry (nLCMS/MS) analysis.

Following nLC-MS/MS and data analysis, initial comparisons suggested that both extraction methods yielded comparable numbers of proteins, with on average, slightly more proteins recovered using the supernatant & pellet approach. This latter method was adopted for all remaining samples, with slight modification in the volume of EDTA for sample demineralisation to eliminate the need for protein concentration using the Amicon ultra-filter.

Mass spectrometry was conducted at two different laboratories: the nine pilot samples were analyzed at the Target Discovery Institute, Nuffield Department of Medicine, University of Oxford (TDI); while the remaining samples were analyzed at the Centre for Excellence in Mass Spectrometry at the University of York (CoEMS). At the TDI, MS data was acquired on an LC-MS/MS platform consisting of Q-Exactive mass spectrometer and Dionex Ultimate 3000 UPLC (both Thermo Scientific). Samples were separated with a 60 minute long linear gradient of 2% to 35% Acetonitrile in 5% DMSO/0.1% FA, using an Easyspray column (75um x 500mm) and a flowrate of 250nl/s. MS1 resolution was set to 70,000 with an AGD target of 3E6 between 380 and 1800 m/z. Up to 15 precursor ions were selected for MS2 acquisition at a resolution of 17,500 for up to 128ms and an isolation window of 1.6m/z. Collision energy was set to 28% and selected precursors were excluded from re-selection for 27 seconds.

At the CoEMS tryptic peptides were analysed using a n-LC interfaced with an Orbitrap Fusion hybrid mass spectrometer. Samples were loaded onto an UltiMate 3000 RSLCnano HPLC system (Thermo) equipped with a PepMap 100 Å C18, 5 μm trap column (300 μm x 5 mm Thermo) and a PepMap, 2 μm, 100 Å,C18 EasyNano nanocapillary column (75 μm x 550 mm, Thermo).

Raw spectral data for all samples were converted to Mascot generic format (MGF) using Proteowizard MS Convert (version 3.0.4743). Database searching of tandem mass spectra was performed using Mascot (Matrix ScienceTM, version 2.4.01), against UniProt and the Human Oral Microbiome database previously published in Warinner et al. [19]. Searches were performed against a decoy database to generate empirical protein false discovery rates, which were adjusted to less than 5%. Propionamide (C) was set as a fixed modification and acetylation (protein N-terminus), deamidation (NQ), oxidation (M), propionamide (K) and propionamide (N-terminus) were set as variable modifications. Peptide tolerance was 3 ppm, and MS/MS ion tolerance was 0.5 Da. For those five samples, where multiple extractions were performed on supernatant and pellets fractions, raw data from both extractions were combined before searching through Mascot. Tryptic peptides were searched with up to 1 missed cleavage. Protein results were filtered to a false discovery rate of less than 5% and a significance threshold p< 0.05; only proteins containing a minimum of two peptides were considered. Following the approach in Hendy *et al.* [18], we assigned Mascot protein family identifications to the following classes: contaminants, mammalian, plants, fish, and microbes. We took a conservative approach and assigned any protein identified in our blank controls or injection blanks to the ‘contaminant’ category. Initially, all non-human animal and plant proteins were considered as potential dietary proteins and were further interrogated using BLAST (NCBI). Any non-human animal or plant peptides that also matched identically to human or microbial proteins were not considered as possible dietary proteins. Likewise, any non-human animal or plant peptides deriving from proteins identified within the ‘contaminant’ dataset were also eliminated as potential dietary proteins. Single peptide matches are presented for dietary protein in Table S4.3; the raw data and Mascot search results are available in the public database on the MassIVE repository (ID: MSV000083853; http://massive.ucsd.edu) (file description and run order presented in Supplementary Information 6).

References

1. Naumov G, Mitkoski A, Talevski H, Antolin F, Gibaja JF, Stojanovski D, et al. Research of Vrbjanska Chuka 2016 - 2019. (in Prep.

2. Whittle A, Bartosiewicz L, Borić D, Pettitt P, Richards MP. In the beginning: new radiocarbon dates for the Early Neolithic in northern Serbia and south-east Hungary. Antaeus. 2002;25:63–118.

3. Tasić N. Few new radiocarbon dates from Donja Branjevina and Magareći Mlin sites. Glas Srp Arheol Društva. 1993;9:99–102.

4. Correa-Ascencio M, Evershed RP. High throughput screening of organic residues in archaeological potsherds using direct acidified methanol extraction. Anal Methods. 2014;6(5):1330–40.

5. Casanova E, Knowles TDJ, Williams C, Crump MP, Evershed RP. Practical Considerations in High-Precision Compound-Specific Radiocarbon Analyses: Eliminating the Effects of Solvent and Sample Cross-Contamination on Accuracy and Precision. Anal Chem. 2018;90:11025–32.

6. Casanova E, Knowles TDJ, Bayliss A, Dunne J, Baranski MZ, Denaire A, et al. Accurate compound-specific radiocarbon dating of archaeological pottery vessels.

7. Naumov G, Mitkoski A, Murgoski A, Benes J, Przybyla M, Komarkova V, et al. Research on Vrbjanska Čuka site at Slavej - 2016. Patrimonium. 2016;9(14):13–42.

8. Naumov G, Mitkoski A, Talevski H, Murgoski A, Dumurđanov N, Benes J, et al. Research of Vrbjanska Čuka in 2017. Balcanoslavica. 2018;47(1):253–85.

9. Leković V. Magareći Mlin - Apatin. In: Srejović D, editor. The Neolithic of Serbia Archaeological Research 1948-1988. Belgrade: Centre for Archaeological Research, Faculty of Philosophy, University of Belgrade; 1989. p. 79–80.

10. Clason AT. Padina and Starčevo: game, fish and cattle. Palaeohistoria. 1980;22:141–73.

11. Živković M. Zaštitna istraživanja arheološkog nalazišta Grad–Starčevo 2003. i 2004. godine. Arheol Pregl. 2008;2/3:11.

12. Živković M, Vukadinović M, Antonović D. Geofizička i arheološka istraživanja u Starčevu: novi pristup zaštiti neolitskog naselja. Rad Muzeja Vojv. 2011;53:7–22.

13. Brown WAB, Christofferson P V., Massler M, Weiss MB. Postnatal tooth development in cattle. Am J Vet Res. 1960;21(80):7–34.

14. Ducos P. Origine des animaux domestiques in Palestine. Mémoire no. Publications de l’Institut de Préhistoire de l’Université de Bordeaux. Bordeaux: Delmas; 1968. 191 p.

15. Grant A. The use of tooth wear as a guide to the age of domestic animals. In: Wilson B, Grigson C, Payne S, editors. Ageing and sexing animal bones from archaeological sites. British Se. Oxford: British Archaeological Reports; 1982. p. 91–108.

16. Gillis R. Osteological and isotopic contributions to the study of dairy husbandry during the European Neolithic. PAris: L’école doctorale Sciences de la Nature et de l’Homme, CNRS - Muséum national d’Histoire naturelle; 2012.

17. Fischer R, Kessler BM. Gel-aided sample preparation (GASP)-A simplified method for gel-assisted proteomic sample generation from protein extracts and intact cells. Proteomics. 2015;15(7):1224–9.

18. Hendy J, Warinner C, Bouwman A, Collins MJ, Fiddyment S, Fischer R, et al. Proteomic evidence of dietary sources in ancient dental calculus. Proc R Soc B Biol Sci. 2018;285(1883):20180977.

19. Warinner C, Rodrigues JFM, Vyas R, Trachsel C, Shved N, Grossmann J, et al. Pathogens and host immunity in the ancient human oral cavity. Nat Genet. 2014;46(4):336–44.
